# Supplementary material for: Effectiveness of spatially targeted interventions for control of HIV, tuberculosis, leprosy and malaria: a systematic review
Source: BMJ Open. 2021 Jul 13;11(7):e044715. doi: 10.1136/bmjopen-2020-044715 (PMC8278879; doi:10.1136/bmjopen-2020-044715)
Supplement: Supplementary data [file bmjopen-2020-044715supp001.pdf]

Appendix 1

Figure S1: Research Overview

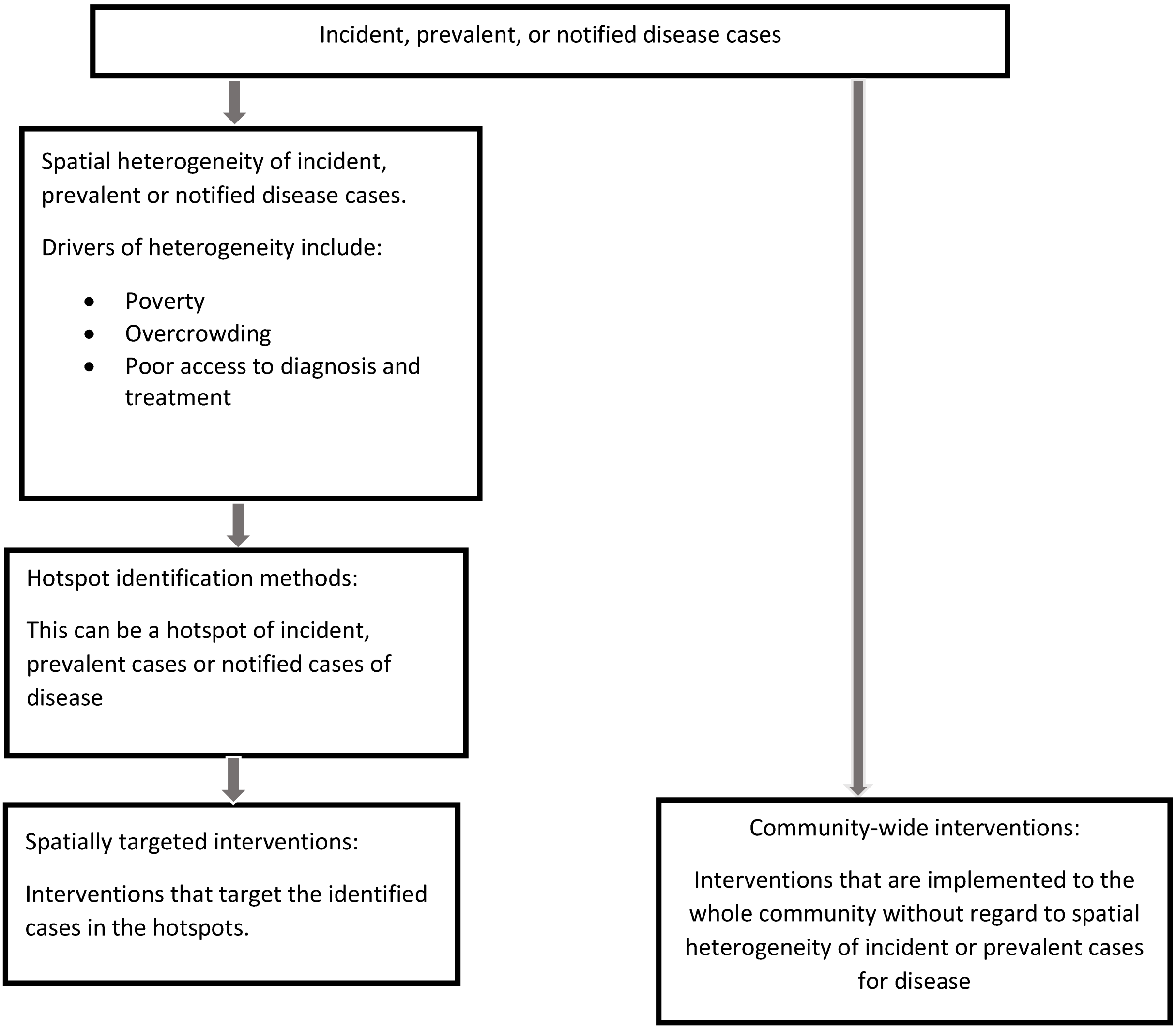

Figure S2: PRISMA flow diagram for the process of selecting included studies

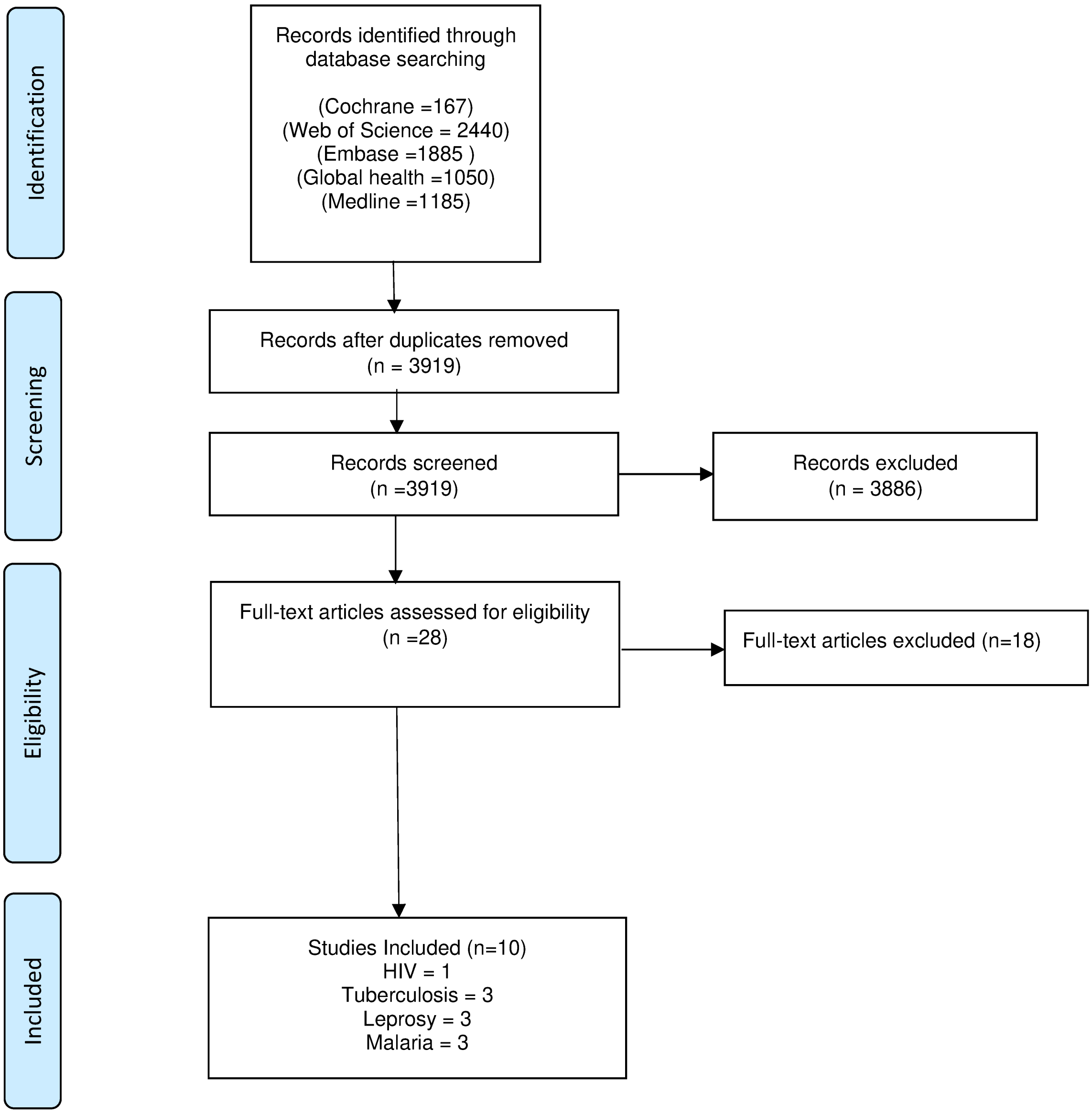

Table S1: Search Strategy

1	(HIV or human immunodeficiency virus or HIV-1 or HIV-2).mp.
2	exp HIV/
3	exp HIV Infections/
4	(aids or acquired immune deficiency syndrome).mp.
5	exp Acquired Immunodeficiency Syndrome/
6	(tuberculosis or TB).mp.
7	exp Tuberculosis/
8	exp Mycobacterium tuberculosis/
9	(malaria* or Plasmodium).mp.
10	exp Malaria/
11	exp Plasmodium/
12	(Leprosy or lepra or lepromatosis or hanseniase).mp.
13	exp Leprosy/
14	exp Mycobacterium leprae/
15	(spatial adj3 (analys?s or regression or temporal or autocorrelation* or auto-correlation* or statistics or epidemiology)).mp.
16	exp spatial analysis/
17	(geographic* adj3 (analys?s or regression or temporal or autocorrelation* or auto-correlation* or system* or epidemiology)).mp.
18	(GPS or global position* system* or GIS or global information system* or space?time or geospatial or hotspot* or hot-spot*).mp.
19	exp Geographic Information Systems/
20	(communit* or neighbo?rhood*).mp.
21	exp Residence Characteristics/
22	(intervention* or target*).mp.
23	((high-burden or highburden) adj3 (area* or region* or tract* or setting*)).mp.
24	(high-incidence adj3 (area* or region* or tract* or setting*)).mp.
25	(high-prevalence adj3 (area* or region* or tract* or setting*)).mp.
26	((hot-spot* or hotspot*) adj3 (area* or region* or tract*)).mp
27	1 or 2 or 3 or 4 or 5 or 6 or 7 or 8 or 9 or 10 or 11 or 12 or 13 or 14
28	15 or 16 or 17 or 18 or 19
29	20 or 21 or 22 or 23 or 24 or 25 or 26
30	27 and 28 and 29
31	limit 30 to english language
32	limit 31 to yr="1993 -Current"

BMJ Publishing Group Limited (BMJ) disclaims all liability and responsibility arising from any reliance placed on this supplemental material which has been supplied by the author(s)

Table S2: Reasons for exclusion of studies

**Reference, year**	**Reason for excluding**
**HIV (three studies)**	
Odek 2014 (1)	HIV hotspots not based on geolocated cases
Ikpeazu 2014 (2)	HIV hotspots not based on geolocated cases
Mburu 2017 (3)	HIV hotspots not based on geolocated cases
**Tuberculosis (four studies)**	
Clark 1999 (4)	Review
Wilkinson 1999 (5)	No hotspots based on geolocated TB cases.
Fatima 2016 (6)	Contact tracing study
Vo 2020 (7)	Hotspots were not based on TB cases
**Malaria (eleven studies)**	
Mnzava 2001 (8)	Cluster randomised trial with no standard of care arm.
Ghosh 2007 (9)	Review
Dongus 2007 (10)	Hotspot not based on geolocated malaria cases
Cook 2007 (11)	No spatially targeted intervention was evaluated
Shiff 2012 (12)	No spatially targeted intervention was evaluated
Marston 2014 (13)	Health economics study
Cook 2014 (14)	Hotspot not based on geolocated malaria cases
Hsiang 2019 (15)	Defining optimal strategies for contact tracing strategies
Ousmane 2019 (16)	No clear definition of hotspot and study evaluation not based on hotspots.
Ngo 2019 (17)	Feasibility study of acceptability of geographic information technology
Bhondoekhan 2020 (18)	Contact tracing study

Table S3: Full description of the characteristics of spatially targeted interventions of HIV, TB, Leprosy and Malaria

Reference, year	Setting	Study type	Intervention	Control
**HIV (one study)**				
Goswami2012(19)	Wake County, North Carolina USA	Implementation study	Community screening of HIV at sites in the hotspot areas by community nurses and disease intervention specialists from the HIV, syphilis and TB clinics at the county health department.	HIV screening of patients going to STD clinic outside of hotspot areas in the same county.
**Tuberculosis (three studies)**				
Moonan, 2006(20)	Tarrant County, north central Texas, USA	Implementation study	Community tuberculin skin testing and active TB screening by community organisations in hotspots (period 2002-2004)	No control or comparison group.
Goswami2012(19)	Wake County, North Carolina USA	Implementation study	Community screening of latent TB at sites in the hotspot areas by community nurses and disease intervention specialists from the HIV, Syphilis and TB clinics at the county health department.	Screening latent TB in patients going to a TB clinic outside of hotspot areas in the same county.
Cegielski2013 (21)	Smith County, Texas USA	Implementation study	Community screening of tuberculin skin testing and treatment of diagnosed in the hotspots (1996)	No control or comparison group
**Leprosy (three studies)**				

DE Souza Dias2007(22)	Municipality of Mossoro, Rio Grande do Norte, Brazil	Implementation study	Study team members moved door to door in the hotspots to identify people with symptoms of leprosy and referred them to the nearest primary health clinic for diagnosis (period 2005)	No control group
Jim2010(23)	state of Pohnpei in the Federated State of Micronesia.	Implementation study	Door to door community Screening of Leprosy cases in the hotspots (2007 to 2009)	No control group
Barreto2015(24)	Municipalities of Castanhal and Oriximinal in the state of Para, Brazil	Implementation study	Community leprosy case screening in two public schools located in the hotspot	Prevalence of leprosy in children from randomly selected schools not from the hotspot areas.
**Malaria (three studies)**				
Srivastava2009(25)	central Indian state of Madhya Predesh	Implementation study	Not specifically listed. (2007)	No control

BMJ Publishing Group Limited (BMJ) disclaims all liability and responsibility arising from any reliance placed on this supplemental material which has been supplied by the author(s)

Herdiana2013(26)	Island district of Sabang in Indonesia	Implementation study	Twice a month visit households. At each visit screen for malaria from household members with fever or recent history of fever. Treat all confirmed malaria cases.(May 2010 onwards)	Once a month to households in the hotspots. At each visit screen for malaria from household members with fever or recent history of fever. Treat all confirmed malaria cases. (May 2010 onwards)
Bousema2016(27)	Rachuonyo, a western district in Kenya	Cluster randomised trial	5 intervention cluster. Weekly larviciding of stagnant water bodies, long lasting treated nets, Indoor residual spraying and mass drug administration to households with confirmed malaria case.(2012)	5 cluster control areas. Annual residual spraying, distribution of long lasting treated nets at antenatal clinics. No drug mass administration

Abbreviations: HIV Human Immunodeficiency Virus, STD Sexually Transmitted Disease,TB Tuberculosis.

Reference

1. Odek WO, Githuka GN, Avery L, Njoroge PK, Kasonde L, Gorgens M, et al. Estimating the size of the female sex worker population in Kenya to inform HIV prevention programming. PLoS One. 2014 Mar 3;9(3).

2. Ikpeazu A, Momah-Haruna A, Madu Mari B, Thompson LH, Ogungbemi K, Daniel U, et al. An appraisal of female sex work in Nigeria--implications for designing and scaling up HIV prevention programmes. PLoS One [Internet]. 2014;9(8):e103619. Available from: http://ovidsp.ovid.com/ovidweb.cgi?T=JS&PAGE=reference&D=med10&NEWS=N&AN=25118691

3. Mburu G, Ngin C, Tuot S, Chhoun P, Pal K, Yi S. Patterns of HIV testing, drug use, and sexual behaviors in people who use drugs: findings from a community-based outreach program in Phnom Penh, Cambodia. Addict Sci Clin Pract [Internet]. 2017 Dec 19 [cited 2020 Mar 1];12(1):27. Available from: http://ovidsp.ovid.com/ovidweb.cgi?T=JS&PAGE=reference&D=med13&NEWS=N&AN=29202872

4. Clark PA, Cegielski JP, Hassell W. TB or not TB? Increasing door-to-door response to screening. Public Health Nurs [Internet]. 1997;14(5):268–71. Available from: http://ovidsp.ovid.com/ovidweb.cgi?T=JS&PAGE=reference&D=med4&NEWS=N&AN=9342917

5. Wilkinson D, Tanser F, D. W, F. T. GIS/GPS to document increased access to community-based treatment for tuberculosis in Africa. Lancet [Internet]. 1999;354(9176):394–5. Available from: http://ovidsp.ovid.com/ovidweb.cgi?T=JS&PAGE=reference&D=emed6&NEWS=N&AN=29355762

6. Fatima R, Qadeer E, Yaqoob A, Haq MU, Majumdar SS, Shewade HD, et al. Extending “Contact Tracing” into the Community within a 50-Metre Radius of an Index Tuberculosis Patient Using Xpert MTB/RIF in Urban, Pakistan: Did It Increase Case Detection?. PLoS One [Internet]. 2016;11(11):e0165813. Available from: http://ovidsp.ovid.com/ovidweb.cgi?T=JS&PAGE=reference&D=med12&NEWS=N&AN=27898665

7. Vo LNQ, Codlin AJ, Forse RJ, Nguyen NT, Vu TN, Le GT, et al. Evaluating the yield of systematic screening for tuberculosis among three priority groups in Ho Chi Minh City, Viet Nam. Infect Dis Poverty [Internet]. 2020 Dec 1 [cited 2021 Apr 2];9(1):166. Available from: https://idpjournal.biomedcentral.com/articles/10.1186/s40249-020-00766-4

8. Mnzava AE, Sharp BL, Mthembu DJ, le Sueur D, Dlamini SS, Gumede JK, et al. Malaria control–two years’ use of insecticide-treated bednets compared with insecticide house spraying in KwaZulu-Natal. South African Med J [Internet]. 2001;91(11):978–83. Available from: https://www.cochranelibrary.com/central/doi/10.1002/central/CN-00377979/full

9. Ghosh SK, Dash AP. Larvivorous fish against malaria vectors: a new outlook. Trans R Soc Trop Med Hyg [Internet]. 2007;101(11):1063–4. Available from: http://ovidsp.ovid.com/ovidweb.cgi?T=JS&PAGE=reference&D=med5&NEWS=N&AN=17804029

10. Dongus S, Nyika D, Kannady K, Mtasiwa D, Mshinda H, Fillinger U, et al. Participatory mapping of target areas to enable operational larval source management to suppress malaria vector mosquitoes in Dar es Salaam, Tanzania. Int J Health Geogr [Internet]. 2007 Sep 4 [cited 2020 Mar 2];6:37. Available from: http://www.ncbi.nlm.nih.gov/pubmed/17784963

11. Cook J, Aydin-Schmidt B, Gonzalez IJ, Bell D, Edlund E, Nassor MH, et al. Loop-mediated isothermal amplification (LAMP) for point-of-care detection of asymptomatic low-density malaria parasite carriers in Zanzibar. Malar J [Internet]. 2015;14:43. Available from: http://ovidsp.ovid.com/ovidweb.cgi?T=JS&PAGE=reference&D=med11&NEWS=N&AN=25627037

12. Shiff C. Utilising malariometric data in Real Time: a strategy to roll back malaria and sustain local elimination. Malar J. 2012 Oct 15;11(S1):1–1.

13. Marston L, Kelly GC, Hale E, Clements ACA, Hodge A, Jimenez-Soto E. Cost analysis of the development and implementation of a spatial decision support system for malaria elimination in Solomon Islands. Malar J [Internet]. 2014;13(325). Available from: http://www.malariajournal.com/content/pdf/1475-2875-13-325.pdf

14. Cook J, Xu W, Msellem M, Vonk M, Bergstrom B, Gosling R, et al. Mass screening and treatment on the basis of results of a Plasmodium falciparum-specific rapid diagnostic test did not reduce malaria incidence in Zanzibar. J Infect Dis [Internet]. 2015;211(9):1476–83. Available from: http://ovidsp.ovid.com/ovidweb.cgi?T=JS&PAGE=reference&D=med11&NEWS=N&AN=25429102

15. Hsiang MS, Ntshalintshali N, Kang Dufour M-S, Dlamini N, Nhlabathi N, Vilakati S, et al. Active case-finding for malaria: A three-year national evaluation of optimal approaches to detect infections and hotspots through reactive case detection in the low transmission setting of Eswatini. Clin Infect Dis [Internet]. 2019; Available from: http://ovidsp.ovid.com/ovidweb.cgi?T=JS&PAGE=reference&D=medp&NEWS=N&AN=31095677

16. Sy O, Niang EHA, Diallo A, Ndiaye A, Konate L, Ba EHCC, et al. Evaluation of the effectiveness of a targeted community-based IRS approach for malaria elimination in an area of low malaria transmission of the central-western Senegal. Parasite Epidemiol Control [Internet]. 2019;6:e00109. Available from: http://ovidsp.ovid.com/ovidweb.cgi?T=JS&PAGE=reference&D=prem&NEWS=N&AN=31193475

17. Ngo TD, Canavati SE, Dinh HS, Ngo TD, Tran DT, Martin NJ, et al. Addressing operational challenges of combatting malaria in a remote forest area of Vietnam using spatial decision support system approaches. Geospat Health [Internet]. 2019 Nov 6 [cited 2021 Apr 2];14(2):195–203. Available from: https://geospatialhealth.net/index.php/gh/article/view/770

18. Bhondoekhan FRP, Searle KM, Hamapumbu H, Lubinda M, Matoba J, Musonda M, et al. Improving the efficiency of reactive case detection for malaria elimination in southern Zambia: A cross-sectional study. Malar J [Internet]. 2020 May 7 [cited 2021 Apr 2];19(1):175. Available from: https://malariajournal.biomedcentral.com/articles/10.1186/s12936-020-03245-1

19. Goswami ND, Hecker EJ, Vickery C, Ahearn MA, Cox GM, Holland DP, et al. Geographic information system-based screening for TB, HIV, and syphilis (GIS-THIS): a cross-sectional study. Pai M, editor. PLoS One [Internet]. 2012 Oct 2 [cited 2019 Mar 29];7(10):e46029. Available from: http://ovidsp.ovid.com/ovidweb.cgi?T=JS&PAGE=reference&D=med8&NEWS=N&AN=23056227

20. Moonan PK, Oppong J, Sahbazian B, Singh KP, Sandhu R, Drewyer G, et al. What is the

BMJ Publishing Group Limited (BMJ) disclaims all liability and responsibility arising from any reliance placed on this supplemental material which has been supplied by the author(s)

outcome of targeted tuberculosis screening based on universal genotyping and location? Am J Respir Crit Care Med [Internet]. 2006;174(5):599–604. Available from: http://ovidsp.ovid.com/ovidweb.cgi?T=JS&PAGE=reference&D=med5&NEWS=N&AN=16728707

21. Cegielski JP, Griffith DE, McGaha PK, Wolfgang M, Robinson CB, Clark PA, et al. Eliminating tuberculosis one neighborhood at a time. Am J Public Health [Internet]. 2013 Jul [cited 2019 Feb 5];103(7):1292–300. Available from: http://www.ncbi.nlm.nih.gov/pubmed/23078465

22. De Souza Dias MCF, Dias GH, Nobre ML, M.C.F. DSD, G.H. D, M.L. N. The use of Geographical Information System (GIS) to improve active leprosy case finding campaigns in the Municipality of Mossoro, Rio Grande do Norte State, Brazil. Lepr Rev [Internet]. 2007;78(3):261–9. Available from: http://www.lepra.org.uk/lr/Sept07/Lep261-269.pdf

23. Jim R, Johnson E, Pavlin BI. Role of GIS technology during leprosy elimination efforts in Pohnpei. Pac Health Dialog [Internet]. 2010;16(1):109–14. Available from: http://ovidsp.ovid.com/ovidweb.cgi?T=JS&PAGE=reference&D=med7&NEWS=N&AN=20968242

24. Barreto JG, Bisanzio D, Frade MAC, Moraes TMP, Gobbo AR, Guimarães L de S, et al. Spatial epidemiology and serologic cohorts increase the early detection of leprosy. BMC Infect Dis [Internet]. 2015 Nov 16 [cited 2019 Mar 12];15(1):1–9. Available from: http://www.ncbi.nlm.nih.gov/pubmed/26573912

25. Srivastava A, Nagpal BN, Joshi PL, Paliwal JC, Dash AP, A. S, et al. Identification of malaria hot spots for focused intervention in tribal state of India: a GIS based approach. Int J Health Geogr [Internet]. 2009;8(101152198):30. Available from: http://ovidsp.ovid.com/ovidweb.cgi?T=JS&PAGE=reference&D=med6&NEWS=N&AN=19457227

26. Herdiana H, Fuad A, Asih PB, Zubaedah S, Arisanti RR, Syafruddin D, et al. Progress toward malaria elimination in sabang, aceh, indonesia. Am J Trop Med Hyg [Internet]. 2012;87(5 SUPPL. 1):47. Available from: http://www.ajtmh.org/content/87/5_Suppl_1/1.1.full.pdf+html

27. Bousema T, Stresman G, Baidjoe AY, Bradley J, Knight P, Stone W, et al. The Impact of Hotspot-Targeted Interventions on Malaria Transmission in Rachuonyo South District in the Western Kenyan Highlands: A Cluster-Randomized Controlled Trial. PLoS Med. 2016;13(4):e1001993–e1001993.
