# Supplementary material for: Effectiveness of spatially targeted interventions for control of HIV, tuberculosis, leprosy and malaria: a systematic review
Source: BMJ Open. 2021 Jul 13;11(7):e044715. doi: 10.1136/bmjopen-2020-044715 (PMC8278879; doi:10.1136/bmjopen-2020-044715)
Supplement: Author's manuscript [file bmjopen-2020-044715.draft_revisions.pdf]

BMJ Open is committed to open peer review. As part of this commitment we make the peer review history of every article we publish publicly available.

When an article is published we post the peer reviewers' comments and the authors' responses online. We also post the versions of the paper that were used during peer review. These are the versions that the peer review comments apply to.

The versions of the paper that follow are the versions that were submitted during the peer review process. They are not the versions of record or the final published versions. They should not be cited or distributed as the published version of this manuscript.

BMJ Open is an open access journal and the full, final, typeset and author-corrected version of record of the manuscript is available on our site with no access controls, subscription charges or pay-per-view fees (http://bmjopen.bmj.com).

If you have any questions on BMJ Open's open peer review process please email info.bmjopen@bmj.com

BMJ Open

EFFECTIVENESS OF SPATIALLY-TARGETED INTERVENTIONS FOR CONTROL OF HIV, TUBERCULOSIS, LEPROSY AND MALARIA: A SYSTEMATIC REVIEW

Journal:	*BMJ Open*
Manuscript ID	bmjopen-2020-044715
Article Type:	Original research
Date Submitted by the Author:	16-Sep-2020

Complete List of Authors:	Khundi, McEwen; London School of Hygiene & Tropical Medicine, Medical Statistics; Malawi-Liverpool-Wellcome Trust Clinical Research Programme, Public Health<br>Carpenter, James; London School of Hygiene and Tropical Medicine, Medical Statistics; University College London, MRC Clinical Trials Unit<br>Nliwasa, Marriott; University of Malawi College of Medicine, Helse Nord Tuberculosis Initiative<br>Cohen, Ted; Yale University, School of Public Health<br>Corbett, Liz; London School of Hygiene & Tropical Medicine, Department of Clinical Research; Malawi-Liverpool-Wellcome Trust Clinical Research Programme<br>MacPherson, Peter; Liverpool School of Tropical Medicine, Department of Clinical Sciences; Malawi-Liverpool-Wellcome Trust Clinical Research Programme

Keywords:	Tuberculosis < INFECTIOUS DISEASES, Infection control < INFECTIOUS DISEASES, HIV & AIDS < INFECTIOUS DISEASES, Public health < INFECTIOUS DISEASES, Epidemiology < INFECTIOUS DISEASES, INFECTIOUS DISEASES

SCHOLARONE™
Manuscripts

I, the Submitting Author has the right to grant and does grant on behalf of all authors of the Work (as defined in the below author licence), an exclusive licence and/or a non-exclusive licence for contributions from authors who are: i) UK Crown employees; ii) where BMJ has agreed a CC-BY licence shall apply, and/or iii) in accordance with the terms applicable for US Federal Government officers or employees acting as part of their official duties; on a worldwide, perpetual, irrevocable, royalty-free basis to BMJ Publishing Group Ltd ("BMJ") its licensees and where the relevant Journal is co-owned by BMJ to the co-owners of the Journal, to publish the Work in this journal and any other BMJ products and to exploit all rights, as set out in our licence.

The Submitting Author accepts and understands that any supply made under these terms is made by BMJ to the Submitting Author unless you are acting as an employee on behalf of your employer or a postgraduate student of an affiliated institution which is paying any applicable article publishing charge ("APC") for Open Access articles. Where the Submitting Author wishes to make the Work available on an Open Access basis (and intends to pay the relevant APC), the terms of reuse of such Open Access shall be governed by a Creative Commons licence – details of these licences and which Creative Commons licence will apply to this Work are set out in our licence referred to above.

Other than as permitted in any relevant BMJ Author's Self Archiving Policies, I confirm this Work has not been accepted for publication elsewhere, is not being considered for publication elsewhere and does not duplicate material already published. I confirm all authors consent to publication of this Work and authorise the granting of this licence.

For peer review only

EFFECTIVENESS OF SPATIALLY-TARGETED INTERVENTIONS FOR CONTROL OF HIV, TUBERCULOSIS, LEPROSY AND MALARIA: A SYSTEMATIC REVIEW

Corresponding author:
McEwen Khundi
Malawi Liverpool Welcome Trust
P.0 Box 30096
Malawi,
mcewenkhundi@gmail.com
Tel: +265 1811628
Fax: +265 1875774

James Carpenter
Department of medical statistics
London School of Hygiene and Tropical Medicine
London
United Kingdom

Marriot Nliwasa
Department of Pathology
College of Medicine, Malawi
Blantyre
Malawi

Ted Cohen
School of Public Health
Yale University
Connectut, New Haven
USA

Elizabeth L Corbett
Department of Clinical Research
London School of Hygiene and Tropical Medicine
London
United Kingdom

Peter MacPherson
Department of Clinical Sciences
Liverpool School of Tropical Medicine
Liverpool
United Kingdom

Keywords: infectious diseases, community interventions, spatial hotspot, spatially targeted, systematic review.

Word Count:
Abstract: 283
Main text: 4685

For peer review only

Abstract (283 words)

Background: As infectious diseases approach global elimination targets, spatial targeting is increasingly important to identify community hotspots of transmission and effectively target interventions. We aimed to synthesise relevant evidence to define best practice approaches and identify policy and research gaps.

Objective: To systematically-appraise evidence for the effectiveness of spatially-targeted community public health interventions for HIV, Tuberculosis, Leprosy and Malaria.

Design: Systematic review.

Data sources: We searched Medline, Embase, Global Health, Web of Science and Cochrane Database of Systematic Reviews between 01 January 1993 to 22 July 2019.

Study selection: The studies had to include HIV or TB or leprosy or malaria and spatial hotspot definition, and community interventions.

Data extraction and synthesis: A data extraction tool was used. For each study, we summarised approaches to identifying hotpots, intervention design and effectiveness of the intervention.

Results: Thirteen studies, including 2 cluster randomised trials and 11 with alternative designs (before-after, comparator area), satisfied our inclusion criteria. Spatially targeted interventions for HIV (1 USA study), TB (3 USA, 1 Pakistan) and leprosy (two Brazil, one Federated States of Micronesia) each used household location and disease density to define hotspots followed by community-based screening. Malaria studies (one each from India, Indonesian, Eswatini and Kenya) used household location and disease density for hotspot identification followed by complex interventions typically combining community screening, larviciding of stagnant water bodies, indoor residual spraying and mass drug administration. Evidence of effect was mixed.

Conclusions: Studies investigating spatially targeted interventions were few in number, and mostly underpowered or otherwise limited methodologically, affecting interpretation of intervention impact. Applying advanced epidemiological methodologies supporting more robust hotspot identification and larger or more intensive interventions would strengthen the evidence-base for this increasingly important approach.

PROSPERO registration: CRD42019130133

For peer review only

For peer review only - http://bmjopen.bmj.com/site/about/guidelines.xhtml

Strengths and limitations of the study

- This was a thoroughly conducted systematic review which only included published literature.
- We developed the search strategy with input from infectious disease experts, statisticians and librarians, and we only included studies that met our objective assessment criteria.
- But we acknowledge that some studies might still have been missed even with the systematic approach that we used.
- The effect of publication bias cannot be ruled out as it is possible that studies that had negative results might not have been published; thereby, they would not have been captured by our search strategy.
- The studies had different interventions and outcomes as such a meta-analysis was not done contrary to the initial plans.

For peer review only

Introduction

The world has made tremendous strides in controlling HIV, tuberculosis (TB), leprosy and malaria epidemics. Despite impressive achievements in reductions of new cases, considerable efforts are required to meet and maintain elimination targets (1–4). Much of the current progress in disease control is attributable to the combination of facility based routine health services, supplemented by community based interventions (1–4).

The success of disease control strategies provided through facility-based services relies on prompt recognition of symptoms by the patient, early health seeking by the patient, and correct recognition and management by health providers to provide early diagnosis and effective medical care (1–4). However, health-seeking delays can be prolonged, and this strategy will also have no effect on transmission during subclinical illness (2,5–7). In low-income settings, lack of faith in the quality of services, high opportunity and indirect costs, and non-adherence to syndromic management protocols can combine to cause substantial health-seeking and diagnostic delays, undermining the effectiveness of facility-based strategies based on early diagnosis and treatment (2,4,5,8–12). Community interventions can be complementary to facility-based services but are more resource intensive, logistically challenging and are generally only justifiable when targeted at groups of people at risk or in a geographically defined area with high prevalence or incidence of disease (1–4).

In communities, HIV, TB, leprosy and malaria cluster geographically into *hotspots* (13–19). Hotspots are defined as areas of high incidence or prevalence compared to neighbouring geographical areas (20). For malaria, which is vector-borne, environmental characteristics conducive to replication of anopheles mosquitos are a key consideration (18). For HIV, TB and leprosy, each diseases that are transmitted exclusively person-to-person, hotspots tend to be characterised by poverty, poor access to health services, overcrowding, concentrations of migrant populations, and poor housing (13–18). Since hotspots are likely to be areas of relatively high transmission, targeting interventions at hotspots may prevent many more infections and cases of disease than similar efforts in low-transmission settings or untargeted efforts (**Figure S1**) (21). Public health interventions amenable to spatially-defined hotspot targeting include: screening, case-finding, prevention (including vaccination), and improvement in access to services for diagnosis and treatment (15,20).

Community health and prevention interventions frequently target people in slums or informal urban settlements (22). In low and middle income countries (LMIC), many millions of people live in informal urban settlements (23), and disease control programmes would benefit from more precise identification of high priority areas within these to allow interventions to be delivered to communities where the impact is likely to be greatest (24,25). At the moment targeting tends to use relatively crude

For peer review only - http://bmjopen.bmj.com/site/about/guidelines.xhtml

epidemiological criteria such as age and gender (1–4,26). But since cases of the diseases in this review are known to cluster geographically, targeting of all people in a carefully identified geographically-defined hotspots offers an alternative criteria to define at-risk groups for interventions (13–19).

The main aim of this systematic review was to systematically-appraise evidence for spatially-targeted community public health interventions directed towards major infectious diseases that are transmitted in different ways: HIV is predominantly sexually transmitted; TB by respiratory droplet transmission; leprosy by direct contact and droplet transmission; and malaria by vector-borne transmission. We aim to summarise lessons learnt from evaluation of spatially targeted interventions against these diseases and to make recommendations for researchers, policymakers and disease control programmes, as well as to inform the design and evaluation of future studies investigating spatially-targeted interventions.

For peer review only

For peer review only - http://bmjopen.bmj.com/site/about/guidelines.xhtml

Methods

Study design

Systematic literature review.

Public involvement statement

We developed the research question for this project after realising the evidence gap on the effectiveness of spatially targeted interventions despite the growing interest that this approach has. To develop the scope of the research question we engaged experts in public health interventions of HIV, tuberculosis, leprosy and malaria. The search strategy was also developed in consultation with the experts. The results of the research are going to be disseminated to researchers and policy makers at local and international conferences.

Search strategy

We systematically searched the literature using major subject headings and keywords to identify published studies meeting the inclusion criteria following our published protocol (PROSPERO ID: CRD42019130133). Databases searched included: Medline, Embase, Global Health, Web of Science and the Cochrane Database of Systematic Reviews. The three central concepts included in our search strategy (**Table S1**) were the disease condition (HIV, TB, leprosy and malaria); space (techniques used to identify hotspots); and community interventions.

Eligibility criteria

We included studies published between the period from 01 January 1993 to 22 July 2019. The start of the search period was chosen as the year that TB was declared as a "global emergency" by the World Health Organization (WHO). We focused on studies of spatially targeted interventions for HIV, TB, leprosy and malaria with the aim of identifying all the available literature on the effectiveness of spatially targeted intervention in the selected diseases, and then summarising the findings. We did not limit inclusion by age group of participants or geographic region. We included randomised controlled trials, non-randomised observational studies, before-and-after studies and time-series analysis studies. The following articles were excluded: editorials; narrative and non-systematic reviews; case studies; case reports; case-control studies; contact investigation studies; and non-spatially targeted community studies.

Selection of studies and data extraction

We imported studies into an Endnote (Thompsons Reuters) database, and duplicates were removed. MK and MN independently screened the title and abstracts against inclusion and exclusion criteria

For peer review only - http://bmjopen.bmj.com/site/about/guidelines.xhtml

(**Table S2**), identified studies eligible for full-text review, and subsequently reviewed the full text of each selected study independently. Discrepancies between MK and MN were resolved with discussion, and where agreement could not be reached, two other reviewers (PM and JC) participated in a consensus review.

We developed a data extraction form that was piloted on a sample of selected manuscripts. This form was independently completed for each study selected for full-text review by MK and MN.

Assessment of study quality

Two reviewers (MK and MN) assessed the quality of each study. We used separate tools to assess the methodological quality of cluster randomised trials (Cochrane Collaboration risk of bias tool) (27) and non-randomized studies (ROBINS-I tool for risk of bias assessment) (28).

Definitions

We defined spatially-targeted interventions as community interventions that targeted hotspots of disease. Hotspots were defined as sub-district geospatially-defined areas that had a high number of incident or prevalent cases of the infection or disease compared to surrounding areas. In practice, as definitions varied considerably between studies, we extracted and compared hotspot definitions between studies.

Statistical analysis

The main objective of the systematic review was to compare the impact of the intervention in the intervention hotspot areas compared to control areas. We report the outcomes defined by the included studies, with outcomes being prevalence, incidence, case notification rates, and number needed to screen to identify a positive case. We calculated measures of effect and uncertainty in effect estimates using data available in manuscripts where these were not provided by the authors using R 3.61 (R Core Team). Because of high anticipated heterogeneity between and within diseases and interventions investigated, we decided not to undertake meta-analysis.

Ethics statement

This review used published data, and ethical review was not required.

Data sharing statement

The search strategy and summary data tables for this systematic review have been deposited at the LSHTM Data Compass repository.

For peer review only - http://bmjopen.bmj.com/site/about/guidelines.xhtml

Results

A total of 2786 unique abstracts were identified by the search strategy, from which 2757 were excluded from the title and abstract review, leaving 29 studies that were reviewed as full-text articles. Overall, 12 studies met the inclusion criteria (**Figure S2**). The reasons for exclusion are found in **Table S2**.

One USA based study was identified for HIV. Four TB studies were found: three studies from the USA and one from Pakistan. Three leprosy studies were found: two from Brazil and one study from the Federated States of Micronesia. For malaria, we identified four studies: one each from India, Indonesia, Eswatini and Kenya. The most common study design was implementation demonstration studies (i.e. studies in which spatially targeted interventions are introduced without random allocation of hotspots to study arms). Nine studies had this design: one HIV, three TB, three leprosy and two malaria. We identified one cluster randomised trial (CRT), which evaluated interventions targeted against malaria (**Table S3)**.

Table 1 summarises the characteristics of the included studies, presenting outcomes, measures of intervention effects and study quality/risk of bias assessment results. **Table 2** synthesises methods used to geolocate cases and identify hotspots in the included studies. **Table S3** has additional study details.

Spatially-targeted HIV interventions

In Goswami et al. (29), cases of TB (n=150), HIV (n=665) and syphilis (n=155) notified between 1 Jan 2005 and 31 Dec 2007 in Wake County, North Carolina USA, were geocoded to households. Kernel density maps of the three diseases were generated; maps identified two areas with the densest number of cases (hotspots). Hotspots were defined as areas with more than 10 cases of either TB, HIV or syphilis notified per square mile during the three years. A map of streets and local businesses of Wake County was used to identify the locations of the hotspot areas.

Between 2 June 2009 and 3 Nov 2011, adult community screening for HIV was done in the defined hotspots at specific sites by community nurses and disease intervention specialists from the HIV, syphilis, and TB clinics at the county health department. Of 247 community participants screened by the study, 240 had valid HIV test results. Prevalence of HIV in hotspot areas was compared to that among patients presenting to a sexually transmitted disease clinic (STD) located outside of the hotspot areas. HIV prevalence was higher among community screened participants (8/240, 3%, 95% CI:1.4-6.5) compared to the Wake County STD clinic (64/15936, 0.4%, 95%CI: 0.3-0.5) with a risk ratio of 8.3

For peer review only

(95% CI: 4.0-17.1, p<0.001). Community HIV screening identified eight HIV positive cases, only one of whom was previously undiagnosed.

Spatially-targeted tuberculosis interventions

In Moonan et al. (24), notified TB cases from 1 Jan 1993 to 31 Dec 2000 (n=991) were geolocated to zip codes in Tarrant County, north-central Texas, USA. Three zip codes were found to have the highest TB notification rates per 100,000 of the population, in addition to having genotypically-clustered *Mycobacterium tuberculosis* isolates (24). The TB notification rates of the zip codes were 94, 55 and 32 cases per 100,000 while the case notification rate for the whole county for the same period was 5.9 cases per 100,000; 95 of 117 (81%) isolates were genotypically clustered in these three zip codes (30). Between 1 Sep 2002 and 31 Dec 2004 community-based organizations offered screening for TB and latent TB infection (LTBI) to hotspot residents. Overall, 3,645 individuals were screened. 1.2% (44) people were diagnosed and treated for active TB, and 18.6% (681) were diagnosed and treated for LTBI. This targeted screening identified one person with active TB for every 83 screened, and one person with LTBI for every five screened. The yield of the targeted approach was considered to be more than what would be expected in a county with an active TB notification rate of 5.7 per 100,000 population year.

Goswami et al. (29) (see above in HIV section) also offered screening for LTBI to the same individuals who were offered HIV testing. Both tests were performed from the same blood sample. Hotspot prevalence was compared to LTBI prevalence at the Wake County TB clinic, which was outside of the hotspot areas. Latent TB testing at the TB clinic was offered to high risk individuals who had close contact with a recently diagnosed active TB cases, refugees and those referred by primary care providers or employers. 234/247 had valid LTBI screening results. LTBI prevalence was higher among community screened participants (36/234, 15%, 95% CI:11.0-21.7) versus 6% (95%CI: 5.6-6.6) at the TB clinic with a risk ratio of 2.5 (95% CI: 1.9-3.5, p<0.001).

In Cegielski et al. (31), notified TB cases in Smith County, Texas USA between 1985 to 1995 (n=128) and all notified LTBI cases from 1993 to 1995 (n=311) were geocoded to their households. The geocoded cases were loaded into geographical information systems (GIS) software to produce a point map of both active TB and LTBI cases. The two densest neighbourhoods of TB and LTBI cases were identified visually from the map. In 1996, study field workers went door to door in these neighbourhoods and offered tuberculin skin testing (TST). 1291/2258 had LTBI testing of whom 1236 (95.7%) had results read and 229/1236 (18.5%) were TST positive and 147 received treatment. To assess the intervention, the notified TB cases from 1996 to 2006 in Smith County were mapped. The TB notification rates in the targeted hotspots declined from 39.6 per 100,0000 people per year (95% CI:30.4-48.8) from 1985 to 1995 to zero from 1996 to 2006 (p<0.001). While for the entire Smith

For peer review only

County the TB notification rates reduced from 8.1 per 100,0000 people per year (95% CI: 5.2-11.0) from 1985 to 1995 to 3.7 per 100,000 people per year (95% CI: 1.2-6.1) from 1996 to 2006 ($p<0.001$)

In Fatima et al. (32), four districts in Pakistan with densely populated slums (Lahore, Rawalpindi, Faisalabad and Islamabad) were selected for the study. From July 2013 to June 2015, contacts of notified TB patients living within the household (household contacts) or a 50-metre radius of notified index TB cases (non-household contacts) were screened for cough of more than two weeks followed collection of sputum if symptomatic. Of 23,741 presumptive TB patients, 4710 (19.8%) had confirmed TB disease, with similar percentages in household (22.3%) and non-household (19.1%) contacts. 4084 (17%) had a bacteriologically confirmed TB diagnosis. The absolute number of notified cases in the four districts before intervention increased by 7.9% (95% CI: 7.0-8.9, $p < 0.05$) from 100,384 (July 2011 to June 2013) compared to the intervention period 108,341 (July 13-June 15).

Spatially-targeted leprosy interventions

In De Souza Dias et al. (33), leprosy cases notified between 1998 and 2002 (n=368) in the municipality of Mossoro, Rio Grande do Norte in Brazil were geocoded to households. The geocoded cases were used to create a density map with a radius of 100m, and four neighbourhoods with the highest concentration of cases were identified as hotspots. Four active case finding campaigns were conducted in these hotspots between March and September 2005. Study team members went door to door to identify people with symptoms of leprosy and referred them to the nearest primary health clinic. 512 possible leprosy cases were referred, and 104 leprosy cases were diagnosed. The cases identified through hotspot ACF represented 50% of the total cases diagnosed in the city in 2005. In addition, the case notification rate in 2005 was higher than in 2004; 9.34 per 10,000 versus 5.16 per 10000 respectively.

In Jim et al. (34), in the state of Pohnpei in the Federated States of Micronesia, notified leprosy cases from 2002 to 2006 (n=502) were geocoded to households, producing a point-density map of 1 mile radius; areas with the densest areas were identified for ACF. During 2007 to 2008, ACF teams undertook door to door visits and screened household members for leprosy. There was an eight-fold statewide decrease in the number of households that were screened between 2007 to 2009, while the number of identified cases was similar to the pre-spatially targeted ACF campaign period of 2002 to 2006.

In Barreto et al. (35), notified leprosy cases from the two municipalities of Castanhal and Oriximinal in the state of Para, Brazil from January 2004 to February 2010 (n=633) were geocoded to households. The notified leprosy cases were aggregated by census tract to calculate census case notification rates, and a spatially empirical case detection rate was used to smooth notification rates. Census tracts were

For peer review only - http://bmjopen.bmj.com/site/about/guidelines.xhtml

classified into four categories, with the highest category assigned to areas with >40 cases per 100,000 population. Kulldorff's spatial scan statistic was used to define groups of hotspot census tracts with statistically higher than average notification rates. Two schools were selected: one within a hotspot and one from a census tract with ≥40 notified leprosy cases per 100,000 populations. At these schools, 134 students with a mean age of 10 years were screened and 11/134, 8.2% (95% CI:3.5-13.0) were diagnosed with leprosy based on clinical signs and symptoms. The diagnostic yield in hotspot area schools was significantly higher than in students from randomly-selected schools from eight municipalities in the state of Para in a cross-sectional study between 2009 to 2011: 63/1592, 3.9% (95% CI:3.0-4.9) with a risk ratio of 2.1 (95% CI: 1.1-3.8).

Malaria

In Srivastava et al. (36), notified malaria cases between 2000 to 2005 obtained from the State Department of Health in Madhya Predesh were aggregated at block and district level. Malaria hotspots were defined based on the percentage of malaria cases that had *P. falciparum* malaria of all notified cases from 2000 to 2005. Unspecified targeted malaria interventions in 2007 were evaluated for effectiveness by comparing overall number of confirmed cases notified in 2006 with those notified in 2007. Absolute numbers of notified cases decreased by 5.7% (95%CI:4.7-6.7) from 96,042 in 2006 (p < 0.05).

In Herdiana et al. (37), in Sabang island, Indonesia, documented malaria cases from 2007 to 2008 and self-reported malaria cases identified during a survey (n=319), were geocoded to households to produce point maps that classified 14 out of 18 villages as hotspots, based on absolute number of cases. From May 2010, home visits were conducted twice a month in hotspot villages, and only once in non-hotspot villages. Malaria blood smears were taken from anyone with current or recent history of fever, with smear-positive participants referred for treatment. Household contacts and neighbours within 500 meters of smear-positive participants were also screened. Interventions in hotspot areas included: improving malaria diagnostic labs, introduction of Artemisinin-based Combination Therapy(ACT) for malaria treatment, scale-up of indoor residual spraying (IRS) and distribution of long-lasting insecticide-treated nets (LLINs). The incidence of malaria in hotspot areas decreased by 30-fold from 3.18 to 0.13 per 1000 population from 2008 to 2011. (36) (37) (38)

In Bousema et al. (38), between June and July 2011 in Rachuonyo, western Kenya, 17,503 individuals were screened for a malaria prevalence survey, including *P. falciparum* antibodies. SaTScan software was used to define hotspots based on the prevalence of antibody-positivity and age-adjusted antibody density. In total 27 hotspots were identified.

For peer review only

For peer review only - http://bmjopen.bmj.com/site/about/guidelines.xhtml

A randomised controlled trial randomly allocated ten hotspots 1:1 to either intervention or control. The intervention activities were weekly larviciding of stagnant water bodies, provision of LLINs, IRS and mass drug administration (MDA). MDA was only administered to households that had a confirmed malaria case; febrile individuals (temperature > 37.5^0C) and children aged 6mo – 15 years were offered malaria rapid diagnostic tests (RDT). The following standard interventions were available to both arms, hotspots and evaluation zones (the area 500 metres around each hotspot): annual IRS, case management at health facilities and distribution of LLINs from antenatal clinics.

There was no statistically significant difference in parasite prevalence in evaluation zones (area around the hotspot) at 8 weeks and 16 weeks post-intervention time points. The first evaluation zone (1-249m) at the 8th week found 3.6% (95% CI: -2.6 to 9.7%, p=0.216) and the second evaluation zone (250-500m) at the 8th week 3.8% (95% CI: -2.4 to 10.0%, p=0.187). Neither was there any significant difference in the first evaluation zone (1-249m) at the 16th week: 1.0%, (95% CI: -7.0 to 9.1%, p=0.713) and the second evaluation zone (250-500m) at 16th week: 1.0% (95% CI: -8.3 to 10.4%, p=0.809).

In Hsiang et al. (39), between September 2012 and March 2015, notified malaria index cases from public and private health facilities in Eswatini were traced to their households within 48 hours of notification (39). All individuals in the 500m radius area around the index case household were screened for malaria using a rapid diagnostic test (RDT), microscopy and loop-mediated isothermal amplification (LAMP) test as the gold standard. RDT and microscopy positive individuals were referred to the nearest health facility for treatment as per the national treatment policy.

Overall, 1163/1394 (83%) index cases were investigated and 10,890 individuals within the 500-metre radius of the index case were screened. LAMP identified 180 (1.7%) individuals with malaria. The yield of several screening strategies was evaluated against the approach of screening all individuals in the 500-metre radius. Restricting screening to index cases with at least one epidemiological risk factor (female, farming, or lack of vector control) while maintaining screening radius to 500m, reduced by 25% the number of individuals that had to be screened, and identified 82% of infections. Screening only index case household contacts reduced the number of screened individuals by 90% and identified 27% infections. Limiting the screening radius to 200m or the six closest households reduced 40% of individuals from being screened and identified >85% of infections.

For peer review only

Influence of study quality on results

The studies in this review were found to have issues that would make them susceptible to risk of bias. Hotspot selection was based mainly on notified cases (24,29,33–37,39–41). Relying on notified cases alone can introduce selection bias because notified cases can over-represent cases from areas that have good access to health systems (15).

Further, identified geographical hotspots need to be investigated for stability overtime to ensure that they display spatio-temporal consistency in notification trends (15,42). This was not accounted for in the studies included in this review (24,29,33–41).

The majority of the identified studies were implementation/pragmatic studies (24,29,31–37,39). These did not randomise hotspots to interventions and therefore the studies might have compared outcomes of the study in groups of people that had baseline characteristics that were different.

For peer review only

367 **Table 1: Characteristics of spatially targeted interventions for HIV, TB, Leprosy and Malaria**

Reference, year	Outcome	Effect of intervention[1]	Risk of bias assessment
**HIV**			
Goswami 2012 (29)	Comparison of case detection yield between hotspot intervention areas and a county sexually-transmitted disease clinic over the same period (2009-2011)	HIV prevalence was higher among community screened participants (8/240, 3%, 95% CI:1.4-6.5) compared to the Wake County STD clinic (64/15936, 0.4%, 95%CI:0.3-0.5) with a risk ratio of (8.3, 95% CI: 4.0-17.1), p<0.001.	Moderate
**Tuberculosis**			
Moonan2006 (24)	Yield of TB case detection in hotspots.	Targeted screening identified one person with TB for every 83 screened and one person with LTBI for every five screened. The yield of the targeted approach was considered to be more than what would be expected in a county with an active TB notification rate of 5.7 per 100,000 population year.	Critical
Goswami 2012 (29)	Comparison of case detection yield of latent TB between hotspot areas and county TB clinic over the same period (2009-2011)	LTBI prevalence was higher among community screened participants (36/234, 15%, 95% CI:11.0-21.7) versus (541/9024, 6%, 95%CI=5.6-6.6) at the TB clinic with a risk ratio of (2.5, 95% CI: 1.9-3.5), p<0.001.	Moderate
Cegielski 2013 (31)	A before and after intervention comparison of mapped TB notification rates between 1985-1995 and 1996-2006	TB notification rates in the targeted hotspots declined from 39.6 per 100,0000 people per year (95% CI:30.4-48.8) from 1985 to 1995 to zero from 1996 to 2006 (p<0.001)	Serious
Fatima 2016 (32)	Comparison of change in notified TB cases before intervention (July 2011 to June 2013) and during the intervention (July13-June 15).	The number of notified cases in the four districts before intervention 100384 (July 2011 to June 2013) and during the intervention period 108341 (July13-June 15) increased by (7.9%, 95% CI: 7.0-8.9), p < 0.05.	Serious

For peer review only - http://bmjopen.bmj.com/site/about/guidelines.xhtml

**Leprosy (three studies)**			
De Souza Dias 2007 (33)	Percent of notified cases attributable to the intervention, and before and after intervention case notification rate comparison.	ACF identified 50% of the total cases that were diagnosed in 2005. The case notification rate in 2005 was higher compared to pre intervention year 2004, 9.34 per 10000 versus 5.16 per 10000 respectively.	Serious
Jim 2010 (34)	Yield of leprosy case detection during intervention period compared to the pre-intervention period and reduction in households that needed to be screened.	Eight-fold decrease in the number of households that needed to be screened from 2007 to 2009. While still identifying a similar number of new cases to pre-spatially targeted active case finding period 2002 to 2006.	Moderate
Barreto 2015 (35)	The yield of case detection of leprosy cases in school children in hotspot intervention schools versus in children from randomly selected schools.	In the hotspot school's (11/134, 8.2%, 95% CI:3.5-13.0) students with a mean age of 10 years were diagnosed with leprosy. While (63/1592, 3.9%, 95% CI:3.0-4.9) students from randomly selected schools with a mean age of 12 years were diagnosed with leprosy with a risk ratio of (2.1, 95% CI: 1.1-3.8), p<0.05.	Moderate
**Malaria (four studies)**			
Srivastava 2009 (36)	Difference in absolute numbers of notified malaria cases between 2006 and 2007.	An absolute reduction in numbers of notified cases in 2007 (N = 90829) from the notified cases in 2006 (N = 96042), (5.7%, 95% CI:4.7-6.7), p < 0.05.	[2]Unable to make judgement
Herdiana2013 (37)	Change of malaria notification rates from pre-intervention to post-intervention period.	30-fold reduction in malaria notifications from 3.83 per 1000 in 2008 to 0.13 per 1000 in 2011.	Serious

For peer review only - http://bmjopen.bmj.com/site/about/guidelines.xhtml

Bousema2016 (38)	Change in parasite prevalence in the evaluation zones (1-500km from hotspots) of intervention clusters versus control clusters	The first evaluation zone 1-249m at 8th week (3.6%, 95% CI: -2.6 to 9.7%), p=0.216 and the second evaluation zone 250-500m at 8th week (3.8%, 95% CI: -2.4 to 10.0%), p=0.187. The first evaluation zone 1-249m at 16th week (1.0%, 95% CI: -7.0 to 9.1%), p=0.713 and the second evaluation zone 250-500m at 16th week (1.0%, 95% CI: -8.3 to 10.4%), p=0.809	Low
Hsiang 2019 (39)	Investigation screening strategies that would maximize malaria infection test yields compared to screening everyone in the 500-metre radius of an index case.	Screening radius of 200m or 6 closest households would prevent 40% of individuals from being screened and identify > 85% of infections. Limiting screening to index cases with at least 1 epidemiological risk factor (female, farming, or lack of vector control) while maintaining screening radius to 500m prevents 25.2% individuals from screening and identifies 82.1% of infections. Screening only index case household contacts reduced the number of screened individuals by 90.1% and identified 26.7% infections.	Serious

368 [1] Results calculated from the data published in the papers. [2]The specific interventions used were not described in the paper.

For peer review only

369 **Table2: Geolocation of cases and hotspot identification**

Reference, year	Geolocation of cases	Hotspot identification
**HIV (one study)**		
Goswami2012 (29)	Notified cases of either TB (n=150), HIV (n=665) or syphilis (155) between 1 Jan 2005 and 31 Dec 2007 were geolocated to households. The method for geolocation of the cases was not described in the paper.	A kernel density map of the cases was produced. Areas with the highest densities of three diseases of HIV, Syphilis and TB (greater than 10 cases per square mile) were classified as hotspots. Two hotspot neighbourhoods were identified in the county.
**Tuberculosis (four studies)**		
Moonan, 2006 (24)	Notified TB cases (n=991) from 1 Jan 1993 to 31 Dec 2000 in Tarrant County, north central Texas, USA were geolocated to zip codes using residential addresses and zip codes that patients gave at the time of diagnosis with the aid of a GIS software.	Areas with the highest TB notification rates and high percentage of genotypically clustered TB isolates were identified as hotspots. Three neighbourhood hotspots were identified.
Goswami2012 (29)	Notified cases of TB (n=150), HIV (n=665) and syphilis (n= 155) that were notified between 1 Jan 2005 and 31 Dec 2007 were geolocated to households. The method for geolocation of the households was not described in the paper	A kernel density map was developed. Areas with the highest densities of three diseases of HIV, Syphilis and TB (greater than 10 cases per square mile) were classified as hotspots. Two hotspot neighbourhoods were identified in the county.
Cegielski 2013 (31)	Notified TB cases between 1985 to 1995 (n=128) and all notified LTBI from 1993 to 1995 (n=311) were geocoded to their households using the addresses that patients gave at the time of diagnosis. In addition, field workers tracked addresses to households to get household coordinates of addresses that failed to geolocate.	The points of cases were plotted on a map and areas with the densest clusters of points of cases were identified as hotspots, 2 neighbourhoods were identified in the county.

For peer review only - http://bmjopen.bmj.com/site/about/guidelines.xhtml

Fatima2016 (32)	GIS coordinates of the household of index TB case were captured by study field workers using a GIS enabled mobile phone.	The 50 metre radius area around an index case household including the household contacts was designated as the hotspot.
**Leprosy (three studies)**		
DE Souza Dias2007 (33)	Notified leprosy cases that occurred between 1998 and 2002 (n=368) in the municipality of geocoded to households. The method for geolocation of cases was not described.	Density map with a radius of 100m of the notified leprosy cases was produced. Four hotspot areas were identified
Jim2010(34)	Notified leprosy cases from 2002 to 2006 (n=502) were geolocated to households. Field workers visited all notified cases to get household GPS coordinates using a GIS device.	A density map based on 1-mile radius of the notified leprosy cases. Areas with high concentration of cases classified as hotspots.
Barreto2015 (35)	Notified leprosy cases from January 2004 to February 2010 (n=633) were geocoded to households. Field workers visited households of registered cases to collect GPS coordinates.	Hotspots were identified using the Kulldorff's spatial scan statistic and by stratification of the leprosy notified rates. Two hotspots were identified.
**Malaria (four studies)**		
Srivastava2009 (36)	Notified malaria cases between 2000 to 2005 were obtained from the State Department of Health based on the cases notified in clinics in the blocks or districts.	Blocks or districts with a percentage of plasmodium falciparum malaria cases of all notified cases that was either 100% or consistently greater than 30% from 2000-2005 or greater than 70% in 2005
Herdiana2013 (37)	Notified malaria cases from 2007 to 2008 in addition to other self-reported malaria cases that were found during a survey (n=319) were geocoded to households. Field workers obtained the GPS coordinates of households using GIS devices.	Villages that had the majority of malaria cases were classified as hotspots. 14 out of 18 villages were identified
Bousema2016 (38)	June and July 2011, 17,503 individuals tested in a malaria prevalence survey for the prevalence of P. falciparum antibodies (AMA-1 or MSP-10). Field workers collected the GPS coordinates of the households using GPS devices.	Segments of the study area were scanned in the 2 x 4 km rolling windows and areas with higher ($p < 0.05$) prevalence of antibodies and age-adjusted antibody density than the local average values were identified as hotspots.

For peer review only - http://bmjopen.bmj.com/site/about/guidelines.xhtml

Hsiang2019 (39)	GIS coordinates of index TB case were captured by study field workers using GPS device.	Screening of individuals in the 500m radius of an index case including the index cases household contacts.

For peer review only

For peer review only - http://bmjopen.bmj.com/site/about/guidelines.xhtml

Discussion

The key finding of this review is that, despite increasing enthusiasm for the application of spatially targeted interventions in public health research addressing infectious diseases (44), very few studies have rigorously evaluated the effectiveness of such approaches against non-hotspot comparator areas. We identified only 12 studies conducted since 1993, nearly all of which had substantial limitations in how hotspots were defined and identified, how spatially-targeted interventions were evaluated, or how comparator areas were selected. With the limited evidence available, we found some suggestion that hotspot-targeted interventions for tuberculosis, malaria and leprosy may be efficient and effective approaches in increasing diagnostic yield, reducing unnecessary screening, and perhaps in improving disease epidemiology. However, almost all the studies were vulnerable to bias due to regression to the mean: hotspots identified by highest prevalence/incidence will typically see prevalence/incidence decline, even in the absence of an intervention. As such, spatially-targeted approaches hold promise, but require further evaluation in high-quality studies to guide policymakers considering implementing this approach.

We found fewer studies than we anticipated, potentially due to the challenges of collecting spatial data in developing countries where most areas do not yet have municipal address systems or subdistrict postal code systems (15,45). Nevertheless, in recent years technologies for obtaining spatial data have improved, become cheaper and hence more widely available; consequently, the inclusion of spatial data within epidemiological and surveillance studies as a tool to identify disease hotspots has become more common (44). In theory, this should enable disease control programmes to collect spatial data for identifying and targeting hotspots (44)(45). Thus, in future, prioritising complete mapping of low income countries, with introduction of effective residential identification systems should be a major priority. This would allow some of the methods for geolocation of notified cases in the studies in this review, which used manual and labour intensive approaches that also had high potential for imprecision, to be replaced with more efficient approaches (34).

Investment in national geospatial and surveillance tools that allow rapid, accurate and scalable geolocation of cases from within health facilities in settings that lack postal codes would greatly facilitate spatial mapping of areas of interest (52). Such tools would enable the identification of hotspots at finer scale and limit the use of arbitrary blocks (36). One important concern about targeting interventions on the basis of case-notifications alone, however, would be the potential for ascertainment bias to further disadvantage underserved populations from which cases are already under-notified due to limited access to health services (53). For populations where this is likely, the ideal approach would be to investigate disease burden using prevalence surveys, or developing models that include data on geographical distribution of measures of poverty and health service

For peer review only - http://bmjopen.bmj.com/site/about/guidelines.xhtml

access that can be then allow routine notification data to be adjusted to account for likely under-ascertainment (53).

We report a wide variety of interventions in the studies included in this systematic review. For HIV (29), TB (24,29,31,32) and leprosy (33–35), studies tended to focus on community screening interventions in suspected geographic hotspots. Malaria studies (36–39), however, were more focused on complex interventions that combined community screening and mass drug administration, with environmental measures to address the vector, including larviciding of stagnant water bodies and indoor residual spraying.

Despite the above reservations, the studies that we have identified demonstrate the feasibility of the spatially targeted approach to direct resources towards community-based interventions. For case-finding interventions or community screening, spatially targeted interventions are a compromise between tracing known contacts of infectious cases and active case finding interventions across the entire general populations or sub-population (i.e. whole urban slums) (46). One limitation of contact tracing is that it misses out cases that are unknown to the index case, and so will fail to identify cases arising from transmission to casual contacts outside of the household (47,48). Spatially targeted interventions become increasingly important for diseases that are approaching elimination, since these conditions predispose the disease cases to cluster into disease hotspots (13–19) . Hence spatially targeted interventions can help the most successful control programmes to systematically identify and address residual disease hotspots (12,19,33,49).

Among all of the studies reviewed here, only one (Bousema et al.) (38) was assessed as having low risk of bias. This was a malaria intervention that evaluated the impact of a spatially targeted intervention in pre-defined inner and outer regions of hotspots (evaluation zones). This study design allows the evaluation of how targeting the hotspots benefits the individuals that are in the hotspot as well as the individuals that are in the outer regions of the hotspot. This in turn allows assessment of the impact of the intervention on reducing transmission. For vector borne diseases, including malaria, consideration need to be given to the mobility of the vectors themselves when considering spatially targeted interventions (51) i.e. if mosquitoes are able to travel over moderate distances the effect of a hotspot targeted intervention might end up being diluted.

For peer review only

For peer review only - http://bmjopen.bmj.com/site/about/guidelines.xhtml

434 **Conclusion**

435 The rapid increase in cheap, reliable, geolocation technology now provides such a significant tool in
436 the fight to control and eradicate endemic and epidemic infectious diseases that support for rapid
437 development of effective patient mapping systems should be considered essential for health systems
438 in low income countries. Ideally, mapping should be combined with statistical modelling to identify
439 hotspots with the most pressing need for intervention. In this systematic review we provide some
440 evidence in support of the effectiveness of spatially targeted intervention, but with the major
441 conclusion being that the current body of evidence is weak. There is an urgent need to define optimal
442 methodology, allowing recommendations to be made to support the design of more rigorous studies
443 that allow clear evaluation of likely impact to be made for the major categories of infectious diseases,
444 including vector-borne and those transmitted person-to-person. Recommendations should include
445 strategies for timely identification of hotspots and the critical aspects needed to measure the effect
446 of intervention strategies targeting those hotspots.

For peer review only

For peer review only - http://bmjopen.bmj.com/site/about/guidelines.xhtml

Declarations

Ethics approval and consent to participate

This review used published data, and ethical review was not required.

Consent for publication

Not applicable

Availability of data and materials

The search strategy and summary data tables for this systematic review have been deposited at the LSHTM Data Compass repository and all tables used for this analysis have also been included in this paper..

Competing interests

There are no conflicts of interest to declare.

Funding

This work was supported by Overseas Collaborative Site Staff-Studentship included as part of Prof Liz Corbett's Senior Research Fellowship in Clinical Science from Wellcome Trust ("Sustainable Community Action for Lung hEalth (SCALE): a cluster-randomised trial in Blantyre, Malawi." WT200901/Z/16/Z.). The funders had no role in study design; in the collection, analysis, and interpretation of data; in the writing of the report; and in the decision to submit the paper for publication. PM is funded by Wellcome (206575)

Authors' contributions

MK, PM, ELC, and JC designed the study. MK, PM, JC and MN contributed to the data collection and management. MK, PM, JC and MN contributed to the data analysis. MK, PM, ELC, JC, TC and MN contributed to the data interpretation. MK wrote the first draft. MK, PM, ELC, JC, TC and MN reviewed, contributed to, and approved the final draft. All authors read and approved the final manuscript

Acknowledgements

We would like to thank the LSHTM library staff for assisting us with the formulation of the search strategy.

For peer review only

For peer review only - http://bmjopen.bmj.com/site/about/guidelines.xhtml

References

1. United Nations Joint Programme on HIV/AIDS (UNAIDS). UNAIDS data 2018. Geneva; 2018.
2. World Health Organisation. Global tuberculosis report 2018. Geneva; 2018.
3. World Health Oganisation. Weekly leprosy epidemiological report 31AUG2018. Geneva; 2018.
4. World Health Organisation. World malaria report 2018. Geneva; 2018.
5. World Health Organisation. Global leprosy strategy 2016-2020. Geneva; 2015.
6. World Health Organisation. Frequently asked questions on leprosy. Geneva; 2013.
7. Almeida ACG, Kuehn A, Castro AJM, Vitor-Silva S, Figueiredo EFG, Brasil LW, et al. High proportions of asymptomatic and submicroscopic Plasmodium vivax infections in a peri-urban area of low transmission in the Brazilian Amazon. Parasit Vectors. 2018 Dec 20;11(1):194.
8. Ngwira LG, Dowdy DW, Khundi M, Barnes GL, Nkhoma A, Choko AT, et al. Delay in seeking care for tuberculosis symptoms among adults newly diagnosed with HIV in rural Malawi. INT J TUBERC LUNG DIS. 2018;22(3):280–6.
9. Fomundam HN, Tesfay AR, Mushipe SA, Mosina MB, Boshielo CT, Nyambi HT, et al. Prevalence and predictors of late presentation for HIV care in South Africa. S Afr Med J. 2017 Nov 27;107(12):1058–64.
10. Sulat JS, Prabandari YS, Sanusi R, Hapsari ED, Santoso B. The impacts of community-based HIV testing and counselling on testing uptake. J Heal Res. 2018;JHR-01-2018-015.
11. Ho J, Fox GJ, Marais BJ. Passive case finding for tuberculosis is not enough. Int J Mycobacteriology. 2016 Dec 1;5(4):374–8.
12. World Health Organisation. A framework for malaria elimination global Malaria programme. Geneva; 2017.
13. Brian J. Coburn, Justin T. Okano SB, Coburn BJ, Okano JT, Blower S. Using geospatial mapping to design HIV elimination strategies for sub-Saharan Africa. Sci Transl Med. 2017;9(383 PG-).
14. Cuadros DF, Li J, Branscum AJ, Akullian A, Jia P, Mziray EN, et al. Mapping the spatial variability of HIV infection in Sub-Saharan Africa: Effective information for localized HIV prevention and control. Sci Rep. 2017;7(1):1–11.
15. Shaweno D, Karmakar M, Alene KA, Ragonnet R, Clements AC, Trauer JM, et al. Methods used in the spatial analysis of tuberculosis epidemiology: a systematic review. BMC Med. 2018;16(1):193.
16. Monteiro LD, Martins-Melo FR, Brito AL, Alencar CH, Heukelbach J. Spatial patterns of leprosy in a hyperendemic state in Northern Brazil, 2001-2012. Rev Saude Publica. 2015;49.
17. Alencar CH, Ramos AN, dos Santos ES, Richter J, Heukelbach J. Clusters of leprosy transmission and of late diagnosis in a highly endemic area in Brazil: Focus on different spatial analysis approaches. Trop Med Int Heal. 2012;17(4):518–25.
18. Sluydts V, Heng S, Coosemans M, Van Roey K, Gryseels C, Canier L, et al. Spatial clustering and risk factors of malaria infections in Ratanakiri Province, Cambodia. Malar J. 2014;13(101139802):387.
19. Shaweno D, Trauer JM, Denholm JT, McBryde ES. The role of geospatial hotspots in the spatial spread of tuberculosis in rural Ethiopia: a mathematical model. R Soc open Sci. 2018;5(9):180887.
20. Lessler J, Azman AS, McKay HS, Moore SM. What is a Hotspot Anyway? Am J Trop Med Hyg. 2017 Jun;96(6):1270–3.
21. Shrestha S, Chatterjee S, Rao KD, Dowdy DW. Potential impact of spatially targeted adult tuberculosis vaccine in Gujarat, India. J R Soc Interface. 2016;13(116).
22. Lonnroth, Knut. Systematic screening for active tuberculosis Principles and recommendations.
23. United Nations Human Settlements Programme. SLUM ALMANAC 2015 2016 Tracking Improvement in the Lives of Slum Dweller. Nairobi; 2015.
24. Moonan PK, Oppong J, Sahbazian B, Singh KP, Sandhu R, Drewyer G, et al. What is the

For peer review only - http://bmjopen.bmj.com/site/about/guidelines.xhtml

outcome of targeted tuberculosis screening based on universal genotyping and location?. Am J Respir Crit Care Med. 2006;174(5):599–604.

25. Barreto JG, Bisanzio D, Frade MAC, Moraes TMP, Gobbo AR, Guimarães L de S, et al. Spatial epidemiology and serologic cohorts increase the early detection of leprosy. BMC Infect Dis. 2015;15(1):1–9.

26. Wold Health Organisation. Systematic screening for active tuberculosis. Geneva; 2013.

27. Higgins JPT, Altman DG, Gøtzsche PC, Jüni P, Moher D, Oxman AD, et al. The Cochrane Collaboration's tool for assessing risk of bias in randomised trials. BMJ. 2011 Oct 29;343(7829).

28. Sterne JA, Hernán MA, Reeves BC, Savović J, Berkman ND, Viswanathan M, et al. ROBINS-I: A tool for assessing risk of bias in non-randomised studies of interventions. BMJ. 2016 Oct 12;355.

29. Goswami ND, Hecker EJ, Vickery C, Ahearn MA, Cox GM, Holland DP, et al. Geographic information system-based screening for TB, HIV, and syphilis (GIS-THIS): a cross-sectional study. PLoS One. 2012;7(10):e46029.

30. Moonan PK, Bayona M, Quitugua TN, Oppong J, Dunbar D, Jost KCJ, et al. Using GIS technology to identify areas of tuberculosis transmission and incidence. Int J Health Geogr. 2004;3(1):23.

31. Cegielski JP, Griffith DE, McGaha PK, Wolfgang M, Robinson CB, Clark PA, et al. Eliminating tuberculosis one neighborhood at a time. Am J Public Health. 2013;103(7):1292–300.

32. Fatima R, Qadeer E, Yaqoob A, Haq MU, Majumdar SS, Shewade HD, et al. Extending "Contact Tracing" into the Community within a 50-Metre Radius of an Index Tuberculosis Patient Using Xpert MTB/RIF in Urban, Pakistan: Did It Increase Case Detection?. PLoS One. 2016;11(11):e0165813.

33. De Souza Dias MCF, Dias GH, Nobre ML. The use of Geographical Information System (GIS) to improve active leprosy case finding campaigns in the municipality of Mossoro, Rio Grande do Norte State, Brazil. Lepr Rev. 2007;78(3):261–9.

34. Jim R, Johnson E, Pavlin BI. Role of GIS technology during leprosy elimination efforts in Pohnpei. Pac Health Dialog. 2010;16(1):109–14.

35. Barreto JG, Bisanzio D, Frade MAC, Moraes TMP, Gobbo AR, de Souza Guimarães L, et al. Spatial epidemiology and serologic cohorts increase the early detection of leprosy. BMC Infect Dis. 2015 Nov 16;15:527.

36. Srivastava A, Nagpal BN, Joshi PL, Paliwal JC, Dash AP, A. S, et al. identification of malaria hot spots for focused intervention in tribal state of India: a GIS based approach. Int J Health Geogr. 2009;8(101152198):30.

37. Herdiana H, Fuad A, Asih PB, Zubaedah S, Arisanti RR, Syafruddin D, et al. Progress toward malaria elimination in sabang, aceh, indonesia. Am J Trop Med Hyg. 2012;87(5 SUPPL. 1):47.

38. Bousema T, Stresman G, Baidjoe AY, Bradley J, Knight P, Stone W, et al. The Impact of Hotspot-Targeted Interventions on Malaria Transmission in Rachuonyo South District in the Western Kenyan Highlands: A Cluster-Randomized Controlled Trial. PLoS Med. 2016;13(4):e1001993–e1001993.

39. Hsiang MS, Ntshalintshali N, Kang Dufour M-S, Dlamini N, Nhlabathi N, Vilakati S, et al. Active case-finding for malaria: A three-year national evaluation of optimal approaches to detect infections and hotspots through reactive case detection in the low transmission setting of Eswatini. Clin Infect Dis. 2019;

40. Morris U, Msellem MI, Mkali H, Islam A, Aydin-Schmidt B, Jovel I, et al. A cluster randomised controlled trial of two rounds of mass drug administration in Zanzibar, a malaria pre-elimination setting-high coverage and safety, but no significant impact on transmission. BMC Med. 2018;16(1):215.

41. Tripepi G, Jager KJ, Dekker FW, Zoccali C. Selection bias and information bias in clinical research. Nephron - Clin Pract. 2010;115(2).

For peer review only

42. Mosha JF, Sturrock HJW, Greenwood B, Sutherland CJ, Gadalla NB, Atwal S, et al. Hot spot or not: a comparison of spatial statistical methods to predict prospective malaria infections. Malar J. 2014;13(101139802):53.
43. Bousema T, Griffin JT, Sauerwein RW, Smith DL, Churcher TS, Takken W, et al. Hitting hotspots: spatial targeting of malaria for control and elimination. PLoS Med. 2012 Jan;9(1):e1001165.
44. Elliott P, Wartenberg D. Spatial epidemiology: Current approaches and future challenges. Vol. 112, Environmental Health Perspectives. Public Health Services, US Dept of Health and Human Services; 2004. p. 998–1006.
45. Dowdy DW, Dye C, Cohen T. Data Needs for Evidence-Based Decisions: A TB Modeler's "Wish List"
46. Department of HIV/AIDS, Stop TB Department, World Health Organization (WHO). Guidelines for intensified tuberculosis case-finding and isoniazid preventive therapy for people living with HIV in resource-constrained settings. Geneva; 20111.
47. Crampin AC, Glynn JR, Traore H, Yates MD, Mwaungulu L, Mwenebabu M, et al. Tuberculosis transmission attributable to close contacts and HIV status, Malawi. Emerg Infect Dis. 2006;12(5):729–35.
48. Chamie G, Kato-Maeda M, Emperador DM, Wandera B, Mugagga O, Crandall J, et al. Spatial overlap links seemingly unconnected genotype-matched TB cases in rural Uganda. 2018;
49. Busgeeth K, Rivett U. The use of a spatial information system in the management of HIV/AIDS in South Africa. Int J Health Geogr. 2004;3(13).
50. Baidjoe AY, Stevenson J, Knight P, Stone W, Stresman G, Osoti V, et al. Factors associated with high heterogeneity of malaria at fine spatial scale in the Western Kenyan highlands. Malar J. 2016;15(101139802):307.
51. Bousema T, Griffin JT, Sauerwein RW, Smith DL, Churcher TS, Takken W, et al. Hitting hotspots: spatial targeting of malaria for control and elimination. PLoS Med. 2012;9(1):e1001165.
52. MacPherson P, Khundi M, Nliwasa M, Choko AT, Phiri VK, Webb EL, et al. Disparities in access to diagnosis and care in Blantyre, Malawi, identified through enhanced tuberculosis surveillance and spatial analysis. BMC Med. 2019 Dec 29;17(1):21.
53. Borgdorff MW. New measurable indicator for tuberculosis case detection. Emerg Infect Dis. 2004 Sep;10(9):1523–8.
54. Odek WO, Githuka GN, Avery L, Njoroge PK, Kasonde L, Gorgens M, et al. Estimating the size of the female sex worker population in Kenya to inform HIV prevention programming. PLoS One. 2014 Mar 3;9(3).
55. Ikpeazu A, Momah-Haruna A, Madu Mari B, Thompson LH, Ogungbemi K, Daniel U, et al. An appraisal of female sex work in Nigeria--implications for designing and scaling up HIV prevention programmes. PLoS One. 2014;9(8):e103619.
56. Mburu G, Ngin C, Tuot S, Chhoun P, Pal K, Yi S. Patterns of HIV testing, drug use, and sexual behaviors in people who use drugs: findings from a community-based outreach program in Phnom Penh, Cambodia. Addict Sci Clin Pract. 2017;12(1):27.
57. Clark PA, Cegielski JP, Hassell W. TB or not TB? Increasing door-to-door response to screening. Public Health Nurs. 1997;14(5):268–71.
58. Wilkinson D, Tanser F, D. W, F. T. GIS/GPS to document increased access to community-based treatment for tuberculosis in Africa. Lancet. 1999;354(9176):394–5.
59. Sy O, Niang EHA, Diallo A, Ndiaye A, Konate L, Ba EHCC, et al. Evaluation of the effectiveness of a targeted community-based IRS approach for malaria elimination in an area of low malaria transmission of the central-western Senegal. Parasite Epidemiol Control. 2019;6:e00109.
60. Mnzava AE, Sharp BL, Mthembu DJ, le Sueur D, Dlamini SS, Gumede JK, et al. Malaria control–two years' use of insecticide-treated bednets compared with insecticide house spraying in

For peer review only

KwaZulu-Natal. South African Med J. 2001;91(11):978–83.
61. Manin BO, Ferguson HM, Vythilingam I, Fornace K, William T, Torr SJ, et al. Investigating the Contribution of Peri-domestic Transmission to Risk of Zoonotic Malaria Infection in Humans. PLoS Negl Trop Dis. 2016;10(10):e0005064.
62. Majambere S, Fillinger U, Sayer DR, Green C, Lindsay SW. Spatial distribution of mosquito larvae and the potential for targeted larval control in The Gambia. Am J Trop Med Hyg. 2008;79(1):19–27.
63. Marston L, Kelly GC, Hale E, Clements ACA, Hodge A, Jimenez-Soto E. Cost analysis of the development and implementation of a spatial decision support system for malaria elimination in Solomon Islands. Malar J. 2014;13(325).
64. Cook J, Xu W, Msellem M, Vonk M, Bergstrom B, Gosling R, et al. Mass screening and treatment on the basis of results of a Plasmodium falciparum-specific rapid diagnostic test did not reduce malaria incidence in Zanzibar. J Infect Dis. 2015;211(9):1476–83.
65. Ghosh SK, Dash AP. Larvivorous fish against malaria vectors: a new outlook. Trans R Soc Trop Med Hyg. 2007;101(11):1063–4.
66. Dongus S, Nyika D, Kannady K, Mtasiwa D, Mshinda H, Fillinger U, et al. Participatory mapping of target areas to enable operational larval source management to suppress malaria vector mosquitoes in Dar es Salaam, Tanzania. Int J Health Geogr. 2007 Sep 4;6:37.
67. Cook J, Aydin-Schmidt B, Gonzalez IJ, Bell D, Edlund E, Nassor MH, et al. Loop-mediated isothermal amplification (LAMP) for point-of-care detection of asymptomatic low-density malaria parasite carriers in Zanzibar. Malar J. 2015;14:43.
68. Shiff C. Utilising malariometric data in Real Time: a strategy to roll back malaria and sustain local elimination. Malar J. 2012 Oct 15;11(S1):1–1.

For peer review only

For peer review only - http://bmjopen.bmj.com/site/about/guidelines.xhtml

Appendix 1

Figure S1: Research Overview

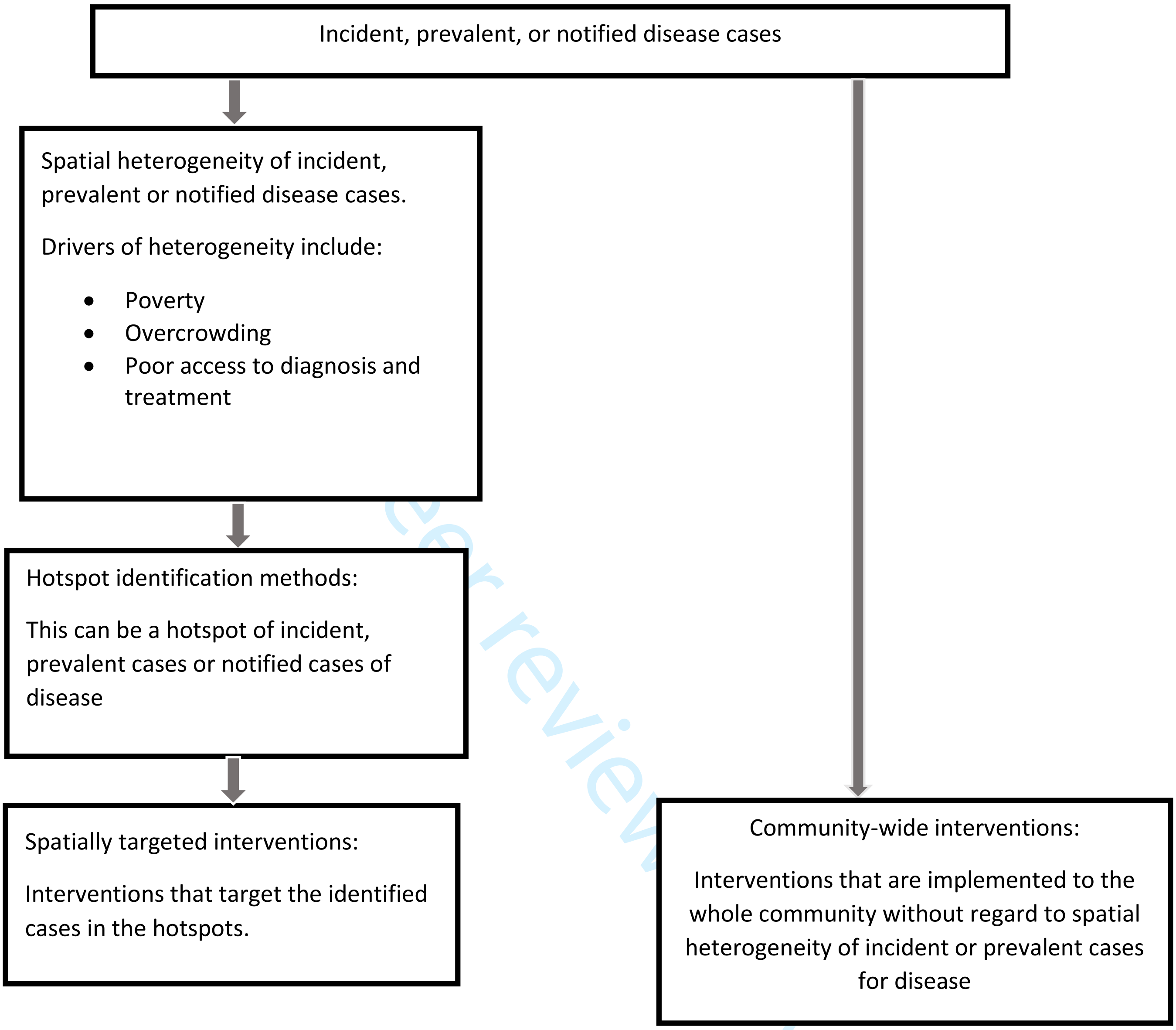

For peer review only - http://bmjopen.bmj.com/site/about/guidelines.xhtml

Figure S2: PRISMA flow diagram for the process of selecting included studies

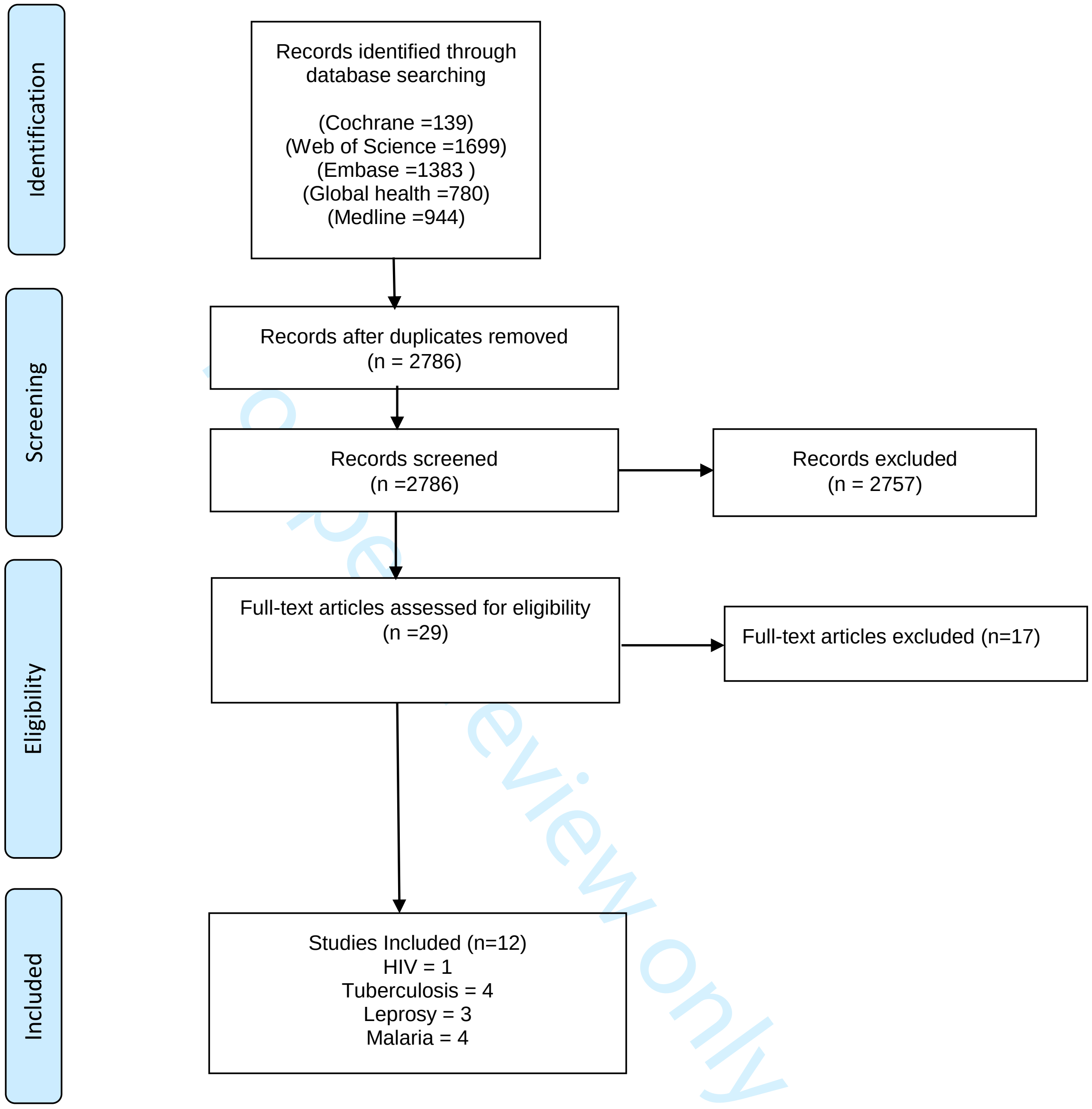

For peer review only

Table S1: Search Strategy

1	(HIV or human immunodeficiency virus or HIV-1 or HIV-2).mp.
2	exp HIV/
3	exp HIV Infections/
4	(aids or acquired immune deficiency syndrome).mp.
5	exp Acquired Immunodeficiency Syndrome/ (75716)
6	(tuberculosis or TB).mp.
7	exp Tuberculosis/
8	exp Mycobacterium tuberculosis/
9	(malaria* or Plasmodium).mp.
10	exp Malaria/
11	exp Plasmodium/
12	(Leprosy or lepra or lepromatosis or hanseniase).mp.
13	exp Leprosy/
14	exp Mycobacterium leprae/
15	(spatial adj3 (analys?s or regression or temporal or autocorrelation* or auto-correlation* or statistics or epidemiology)).mp.
16	exp spatial analysis/ (7792)
17	(geographic* adj3 (analys?s or regression or temporal or autocorrelation* or auto-correlation* or system* or epidemiology)).mp.
18	(GPS or global position* system* or GIS or global information system* or space?time or geospatial or hotspot* or hot-spot*).mp.
19	exp Geographic Information Systems/
20	(communit* or neighbo?rhood*).mp.
21	exp Residence Characteristics/ (60831)
22	(intervention* or target*).mp.
23	((high-burden or highburden) adj3 (area* or region* or tract* or setting*)).mp.
24	(high-incidence adj3 (area* or region* or tract* or setting*)).mp.
25	(high-prevalence adj3 (area* or region* or tract* or setting*)).mp.
26	((hot-spot* or hotspot*) adj3 (area* or region* or tract*)).mp
27	1 or 2 or 3 or 4 or 5 or 6 or 7 or 8 or 9 or 10 or 11 or 12 or 13 or 14
28	15 or 16 or 17 or 18 or 19
29	20 or 21 or 22 or 23 or 24 or 25 or 26
30	27 and 28 and 29
31	limit 30 to english language
32	limit 31 to yr="1993 -Current"

For peer review only

Table S2: Reasons for exclusion of studies

Reference, year	Reason for excluding
**HIV (three studies)**	
Odek 2014 (54)	HIV hotspots not based on geolocated cases
Ikpeazu 2014 (55)	HIV hotspots not based on geolocated cases
Mburu 2017 (56)	HIV hotspots not based on geolocated cases
**Tuberculosis (four studies)**	
Clark 1999 (57)	Review
Wilkinson 1999 (58)	No hotspots based on geolocated TB cases.
**Malaria (ten studies)**	
Ousmane 2019 (59)	No clear definition of hotspot and study evaluation not based on hotspots.
Mnzava 2001 (60)	CRT with no standard of care arm.
Manin 2016 (61)	Case control study
Majembere 2008 (62)	No intervention
Marston 2014 (63)	Health economics study
Cook 2014 (64)	Hotspot not based on geolocated malaria cases
Ghosh 2007 (65)	Review
Dongus 2007 (66)	Hotspot not based on geolocated malaria cases
Cook 2007 (67)	No spatially targeted intervention was evaluated
Shiff 2012 (68)	No spatially targeted intervention was evaluated

For peer review only

For peer review only - http://bmjopen.bmj.com/site/about/guidelines.xhtml

Table S3: Full description of the characteristics of spatially targeted interventions of HIV, TB, Leprosy and Malaria

Reference, year	Setting	Study type	Intervention	Control
**HIV (one study)**				
Goswami2012(29)	Wake County, North Carolina USA	Implementation study	Community screening of HIV at sites in the hotspot areas by community nurses and disease intervention specialists from the HIV, syphilis and TB clinics at the county health department.	HIV screening of patients going to *STD* clinic outside of hotspot areas in the same county.
**Tuberculosis (four studies)**				
Moonan, 2006(24)	Tarrant County, north central Texas, USA	Implementation study	Community tuberculin skin testing and active TB screening by community organisations in hotspots (period 2002-2004)	No control or comparison group.
Goswami2012(29)	Wake County, North Carolina USA	Implementation study	Community screening of latent TB at sites in the hotspot areas by community nurses and disease intervention specialists from the HIV, Syphilis and TB clinics at the county health department.	Screening latent TB in patients going to a TB clinic outside of hotspot areas in the same county.
Cegielski2013 (31)	Smith County, Texas USA	Implementation study	Community screening of tuberculin skin testing and treatment of diagnosed in the hotspots (1996)	No control or comparison group
Fatima2016(32)	Pakistan districts of Lahore,	Extended contact tracing	Screening for active TB in people with a cough of more than 2 weeks in household contacts and neighbours in 50m radius.	No control or comparison group.

For peer review only

	Rawalpindi, Faisalabad and Islamabad			
**Leprosy (three studies)**				
DE Souza Dias2007(33)	Municipality of Mossoro, Rio Grande do Norte, Brazil	Implementation study	Study team members moved door to door in the hotspots to identify people with symptoms of leprosy and referred them to the nearest primary health clinic for diagnosis (period 2005)	No control group
Jim2010(34)	state of Pohnpei in the Federated State of Micronesia.	Implementation study	Door to door community Screening of Leprosy cases in the hotspots (2007 to 2009)	No control group
Barreto2015(35)	Municipalities of Castanhal and Oriximinal in the state of Para, Brazil	Implementation study	Community leprosy case screening in two public schools located in the hotspot	Prevalence of leprosy in children from randomly selected schools not from the hotspot areas.

For peer review only

**Malaria (four studies)**				
Srivastava2009(36)	central Indian state of Madhya Predesh	Implementation study	Not specifically listed. (2007)	No control
Herdiana2013(37)	Island district of Sabang in Indonesia	Implementation study	Twice a month visit households. At each visit screen for malaria from household members with fever or recent history of fever. Treat all confirmed malaria cases.(May 2010 onwards)	Once a month to households in the hotspots. At each visit screen for malaria from household members with fever or recent history of fever. Treat all confirmed malaria cases. (May 2010 onwards)
Bousema2016(38)	Rachuonyo, a western district in Kenya	Cluster randomised trial	5 intervention cluster. Weekly larviciding of stagnant water bodies, long lasting treated nets, Indoor residual spraying and mass drug administration to households with confirmed malaria case.(2012)	5 cluster control areas. Annual residual spraying, distribution of long lasting treated nets at antenatal clinics. No drug mass administration
Hsiang2019 (39)	Eswatini	Extended contact tracing	Community screening of Malaria	No control

For peer review only

64

For peer review only

PRISMA 2009 Checklist

Section/topic	#	Checklist item	Reported on page #
**TITLE**			
Title	1	Identify the report as a systematic review, meta-analysis, or both.	1
**ABSTRACT**			
Structured summary	2	Provide a structured summary including, as applicable: background; objectives; data sources; study eligibility criteria, participants, and interventions; study appraisal and synthesis methods; results; limitations; conclusions and implications of key findings; systematic review registration number.	2
**INTRODUCTION**			
Rationale	3	Describe the rationale for the review in the context of what is already known.	4-5
Objectives	4	Provide an explicit statement of questions being addressed with reference to participants, interventions, comparisons, outcomes, and study design (PICOS).	6-7
**METHODS**			
Protocol and registration	5	Indicate if a review protocol exists, if and where it can be accessed (e.g., Web address), and, if available, provide registration information including registration number.	6
Eligibility criteria	6	Specify study characteristics (e.g., PICOS, length of follow-up) and report characteristics (e.g., years considered, language, publication status) used as criteria for eligibility, giving rationale.	6-7
Information sources	7	Describe all information sources (e.g., databases with dates of coverage, contact with study authors to identify additional studies) in the search and date last searched.	6-7
Search	8	Present full electronic search strategy for at least one database, including any limits used, such that it could be repeated.	6,34
Study selection	9	State the process for selecting studies (i.e., screening, eligibility, included in systematic review, and, if applicable, included in the meta-analysis).	6-7
Data collection process	10	Describe method of data extraction from reports (e.g., piloted forms, independently, in duplicate) and any processes for obtaining and confirming data from investigators.	6-7
Data items	11	List and define all variables for which data were sought (e.g., PICOS, funding sources) and any assumptions and simplifications made.	7,14,15,16
Risk of bias in individual studies	12	Describe methods used for assessing risk of bias of individual studies (including specification of whether this was done at the study or outcome level), and how this information is to be used in any data synthesis.	7,14,15,16
Summary measures	13	State the principal summary measures (e.g., risk ratio, difference in means).	6,7
Synthesis of results	14	Describe the methods of handling data and combining results of studies, if done, including measures of consistency (e.g., I^2) for each meta-analysis.	6,7

For peer review only - http://bmjopen.bmj.com/site/about/guidelines.xhtml

PRISMA 2009 Checklist

Section/topic	#	Checklist item	Reported on page #
Risk of bias across studies	15	Specify any assessment of risk of bias that may affect the cumulative evidence (e.g., publication bias, selective reporting within studies).	7,14,15,16
Additional analyses	16	Describe methods of additional analyses (e.g., sensitivity or subgroup analyses, meta-regression), if done, indicating which were pre-specified.	Not applicable
**RESULTS**			
Study selection	17	Give numbers of studies screened, assessed for eligibility, and included in the review, with reasons for exclusions at each stage, ideally with a flow diagram.	8-13
Study characteristics	18	For each study, present characteristics for which data were extracted (e.g., study size, PICOS, follow-up period) and provide the citations.	7,14,15,16
Risk of bias within studies	19	Present data on risk of bias of each study and, if available, any outcome level assessment (see item 12).	7,14,15,16
Results of individual studies	20	For all outcomes considered (benefits or harms), present, for each study: (a) simple summary data for each intervention group (b) effect estimates and confidence intervals, ideally with a forest plot.	7,14,15,16
Synthesis of results	21	Present results of each meta-analysis done, including confidence intervals and measures of consistency.	7,14,15,16
Risk of bias across studies	22	Present results of any assessment of risk of bias across studies (see Item 15).	7,14,15,16
Additional analysis	23	Give results of additional analyses, if done (e.g., sensitivity or subgroup analyses, meta-regression [see Item 16]).	Not applicable
**DISCUSSION**			
Summary of evidence	24	Summarize the main findings including the strength of evidence for each main outcome; consider their relevance to key groups (e.g., healthcare providers, users, and policy makers).	20,21
Limitations	25	Discuss limitations at study and outcome level (e.g., risk of bias), and at review-level (e.g., incomplete retrieval of identified research, reporting bias).	20,21
Conclusions	26	Provide a general interpretation of the results in the context of other evidence, and implications for future research.	22
**FUNDING**			
Funding	27	Describe sources of funding for the systematic review and other support (e.g., supply of data); role of funders for the systematic review.	23

From: Moher D, Liberati A, Tetzlaff J, Altman DG, The PRISMA Group (2009). Preferred Reporting Items for Systematic Reviews and Meta-Analyses: The PRISMA Statement. PLoS Med 6(7): e1000097. doi:10.1371/journal.pmed1000097

For more information, visit: **www.prisma-statement.org**.

For peer review only - http://bmjopen.bmj.com/site/about/guidelines.xhtml

BMJ Open

EFFECTIVENESS OF SPATIALLY-TARGETED INTERVENTIONS FOR CONTROL OF HIV, TUBERCULOSIS, LEPROSY AND MALARIA: A SYSTEMATIC REVIEW

Journal:	*BMJ Open*
Manuscript ID	bmjopen-2020-044715.R1
Article Type:	Original research
Date Submitted by the Author:	12-Apr-2021
Complete List of Authors:	Khundi, McEwen; London School of Hygiene & Tropical Medicine, Medical Statistics; Malawi-Liverpool-Wellcome Trust Clinical Research Programme, Public Health<br>Carpenter, James; London School of Hygiene and Tropical Medicine, Medical Statistics; University College London, MRC Clinical Trials Unit<br>Nliwasa, Marriott; University of Malawi College of Medicine, Helse Nord Tuberculosis Initiative<br>Cohen, Ted; Yale University, School of Public Health<br>Corbett, Liz; London School of Hygiene & Tropical Medicine, Department of Clinical Research; Malawi-Liverpool-Wellcome Trust Clinical Research Programme<br>MacPherson, Peter; Liverpool School of Tropical Medicine, Department of Clinical Sciences; Malawi-Liverpool-Wellcome Trust Clinical Research Programme
**Primary Subject Heading**:	Public health
Secondary Subject Heading:	Infectious diseases, Epidemiology
Keywords:	Tuberculosis < INFECTIOUS DISEASES, Infection control < INFECTIOUS DISEASES, HIV & AIDS < INFECTIOUS DISEASES, Public health < INFECTIOUS DISEASES, Epidemiology < INFECTIOUS DISEASES, INFECTIOUS DISEASES

SCHOLARONE™
Manuscripts

I, the Submitting Author has the right to grant and does grant on behalf of all authors of the Work (as defined in the below author licence), an exclusive licence and/or a non-exclusive licence for contributions from authors who are: i) UK Crown employees; ii) where BMJ has agreed a CC-BY licence shall apply, and/or iii) in accordance with the terms applicable for US Federal Government officers or employees acting as part of their official duties; on a worldwide, perpetual, irrevocable, royalty-free basis to BMJ Publishing Group Ltd ("BMJ") its licensees and where the relevant Journal is co-owned by BMJ to the co-owners of the Journal, to publish the Work in this journal and any other BMJ products and to exploit all rights, as set out in our licence.

The Submitting Author accepts and understands that any supply made under these terms is made by BMJ to the Submitting Author unless you are acting as an employee on behalf of your employer or a postgraduate student of an affiliated institution which is paying any applicable article publishing charge ("APC") for Open Access articles. Where the Submitting Author wishes to make the Work available on an Open Access basis (and intends to pay the relevant APC), the terms of reuse of such Open Access shall be governed by a Creative Commons licence – details of these licences and which Creative Commons licence will apply to this Work are set out in our licence referred to above.

Other than as permitted in any relevant BMJ Author's Self Archiving Policies, I confirm this Work has not been accepted for publication elsewhere, is not being considered for publication elsewhere and does not duplicate material already published. I confirm all authors consent to publication of this Work and authorise the granting of this licence.

For peer review only

EFFECTIVENESS OF SPATIALLY-TARGETED INTERVENTIONS FOR CONTROL OF HIV, TUBERCULOSIS, LEPROSY AND MALARIA: A SYSTEMATIC REVIEW

Corresponding author:
McEwen Khundi
Malawi Liverpool Welcome Trust
P.0 Box 30096
Malawi,
mcewenkhundi@gmail.com
Tel: +265 1811628
Fax: +265 1875774

James Carpenter
Department of medical statistics
London School of Hygiene and Tropical Medicine
London
United Kingdom

Marriot Nliwasa
Department of Pathology
College of Medicine, Malawi
Blantyre
Malawi

Ted Cohen
School of Public Health
Yale University
Connectut, New Haven
USA

Elizabeth L Corbett
Department of Clinical Research
London School of Hygiene and Tropical Medicine
London
United Kingdom

Peter MacPherson
Department of Clinical Sciences
Liverpool School of Tropical Medicine
Liverpool
United Kingdom

Keywords: infectious diseases, community interventions, spatial hotspot, spatially targeted, systematic review.

Word Count:
Abstract: 287
Main text: 4848

For peer review only

For peer review only - http://bmjopen.bmj.com/site/about/guidelines.xhtml

Abstract (287 words)

Background: As infectious diseases approach global elimination targets, spatial targeting is increasingly important to identify community hotspots of transmission and effectively target interventions. We aimed to synthesise relevant evidence to define best practice approaches and identify policy and research gaps.

Objective: To systematically-appraise evidence for the effectiveness of spatially-targeted community public health interventions for HIV, Tuberculosis, Leprosy and Malaria.

Design: Systematic review.

Data sources: We searched Medline, Embase, Global Health, Web of Science and Cochrane Database of Systematic Reviews between 01 January 1993 and 22 March 2021.

Study selection: The studies had to include HIV or TB or leprosy or malaria and spatial hotspot definition, and community interventions.

Data extraction and synthesis: A data extraction tool was used. For each study, we summarised approaches to identifying hotpots, intervention design and effectiveness of the intervention.

Results: Ten studies, including one cluster randomised trials and nine with alternative designs (before-after, comparator area), satisfied our inclusion criteria. Spatially targeted interventions for HIV (one United States of America (USA) study), TB (three USA) and leprosy (two Brazil, one Federated States of Micronesia) each used household location and disease density to define hotspots followed by community-based screening. Malaria studies (one each from India, Indonesia and Kenya) used household location and disease density for hotspot identification followed by complex interventions typically combining community screening, larviciding of stagnant water bodies, indoor residual spraying and mass drug administration. Evidence of effect was mixed.

Conclusions: Studies investigating spatially targeted interventions were few in number, and mostly underpowered or otherwise limited methodologically, affecting interpretation of intervention impact. Applying advanced epidemiological methodologies supporting more robust hotspot identification and larger or more intensive interventions would strengthen the evidence-base for this increasingly important approach.

PROSPERO registration: CRD42019130133

For peer review only

Strengths and limitations of the study

- This was a thoroughly conducted systematic review which only included published literature.
- We developed the search strategy with input from infectious disease experts, statisticians and librarians, and we only included studies that met our objective assessment criteria.
- We acknowledge that some studies might still have been missed even with the systematic approach that we used.
- The effect of publication bias cannot be ruled out as it is possible that studies that had negative results might not have been published; thereby, they would not have been captured by our search strategy.
- The studies had different interventions and outcomes as such a meta-analysis was not done contrary to the initial plans.

For peer review only

Introduction

The world has made tremendous strides in controlling human immunodeficiency virus (HIV), tuberculosis (TB), leprosy and malaria epidemics. Despite impressive achievements in reductions of new cases, considerable efforts are required to meet and maintain elimination targets (1–4). Much of the current progress in disease control is attributable to the combination of facility based routine health services, supplemented by community based interventions (1–4).

The success of disease control strategies provided through facility-based services relies on prompt recognition of symptoms by the patient, early health seeking by the patient, and correct recognition and management by health providers to provide early diagnosis and effective medical care (1–4). However, health-seeking delays can be prolonged, and this strategy will also have no effect on transmission during subclinical illness (2,5–7). In low-income settings, lack of faith in the quality of services, high opportunity and indirect costs, and non-adherence to syndromic management protocols can combine to cause substantial health-seeking and diagnostic delays, undermining the effectiveness of facility-based strategies based on early diagnosis and treatment (2,4,5,8–12). Community interventions can be complementary to facility-based services but are more resource intensive, logistically challenging and are generally only justifiable when targeted at groups of people at risk or in a geographically defined area with high prevalence or incidence of disease (1–4).

In communities, HIV, TB, leprosy and malaria cluster geographically into hotspots (13–19). Hotspots are defined as areas of high incidence or prevalence compared to neighbouring geographical areas (20). For malaria, which is vector-borne, environmental characteristics conducive to replication of anopheles mosquitos are a key consideration (18). For HIV, TB and leprosy, diseases that are transmitted exclusively person-to-person, hotspots tend to be characterised by poverty, poor access to health services, overcrowding, concentrations of migrant populations, and poor housing (13–18). Since hotspots are likely to be areas of relatively high transmission, targeting interventions at hotspots may prevent many more infections and cases of disease than similar efforts in low-transmission settings or untargeted efforts (**Figure S1**) (21). Public health interventions amenable to spatially-defined hotspot targeting include: screening, case-finding, prevention (including vaccination), and improvement in access to services for diagnosis and treatment (15,20).

Community health and prevention interventions frequently target people in slums or informal urban settlements (22). In low and middle income countries (LMIC), many millions of people live in informal urban settlements (23), and disease control programmes would benefit from more precise identification of high priority areas within these to allow interventions to be delivered to communities where the impact is likely to be greatest (24,25). At the moment targeting tends to use relatively crude

For peer review only

epidemiological criteria such as age and gender (1–4,26). But since cases of the diseases in this review are known to cluster geographically, targeting of all people in a carefully identified geographically-defined hotspots offers an alternative criteria to define at-risk groups for interventions (13–19).

The main aim of this systematic review was to systematically-appraise evidence for spatially-targeted community public health interventions directed towards major infectious diseases that are transmitted in different ways: HIV is predominantly sexually transmitted; TB by respiratory droplet transmission; leprosy by direct contact and droplet transmission; and malaria by vector-borne transmission. We aim to summarise lessons learnt from evaluation of spatially targeted interventions against these diseases and to make recommendations for researchers, policymakers and disease control programmes, as well as to inform the design and evaluation of future studies investigating spatially-targeted interventions.

For peer review only

For peer review only - http://bmjopen.bmj.com/site/about/guidelines.xhtml

Methods

Study design

Systematic literature review.

Public involvement statement

We developed the research question for this project after realising the evidence gap on the effectiveness of spatially targeted interventions despite the growing interest that this approach has. To develop the scope of the research question we engaged experts in public health interventions of HIV, tuberculosis, leprosy and malaria. The search strategy was also developed in consultation with the experts. The results of the research will be disseminated to researchers and policy makers at local and international conferences.

Search strategy

We systematically searched the literature using major subject headings and keywords to identify published studies meeting the inclusion criteria following our published protocol (PROSPERO ID: CRD42019130133). Databases searched included: Medline, Embase, Global Health, Web of Science and the Cochrane Database of Systematic Reviews. The three central concepts included in our search strategy (**Table S1**) were the disease condition (HIV, TB, leprosy and malaria); space (techniques used to identify hotspots); and community interventions.

Eligibility criteria

We included studies published between the period from 01 January 1993 and 22 July 2019 and then updated to 22 March 2021. The start of the search period was chosen as the year that TB was declared as a "global emergency" by the World Health Organization (WHO). We focused on studies of spatially targeted interventions for HIV, TB, leprosy and malaria with the aim of identifying all the available literature on the effectiveness of spatially targeted intervention in the selected diseases, and then summarising the findings. We did not limit inclusion by age group of participants or geographic region. We included randomised controlled trials, non-randomised observational studies, before-and-after studies and time-series analysis studies. The following articles were excluded: editorials; narratives; systematic reviews; case studies; case reports; case-control studies; contact investigation studies; and non- spatially targeted community studies.

Selection of studies and data extraction

We imported studies into an Endnote (Thompsons Reuters) database, and duplicates were removed. McEwen Khundi (MK) and Marriot Nliwasa (MN) independently screened the title and abstracts

For peer review only

For peer review only - http://bmjopen.bmj.com/site/about/guidelines.xhtml

against inclusion and exclusion criteria (**Table S2**), identified studies eligible for full-text review, and subsequently reviewed the full text of each selected study independently. Discrepancies between MK and MN were resolved with discussion, and where agreement could not be reached, two other reviewers (Peter MacPherson (PM) and James Carpenter (JC)) participated in a consensus review.

We developed a data extraction form that was piloted on a sample of selected manuscripts. This form was independently completed for each study selected for full-text review by MK and MN.

Assessment of study quality

Two reviewers (MK and MN) assessed the quality of each study. We used separate tools to assess the methodological quality of cluster randomised trials (Cochrane Collaboration risk of bias tool) (27) and non-randomized studies (ROBINS-I tool for risk of bias assessment) (28).

Definitions

We defined spatially-targeted interventions as community interventions that targeted hotspots of disease. Hotspots were defined as sub-district geospatially-defined areas that had a high number of incident or prevalent cases of the infection or disease compared to surrounding areas, studies that based their hotspots as around diagnosed index cases were not included. In practice, as definitions varied considerably between studies, we extracted and compared hotspot definitions between studies.

Statistical analysis

The main objective of the systematic review was to compare the impact of the intervention in the intervention hotspot areas compared to control areas. We report the outcomes defined by the included studies, with outcomes being prevalence, incidence, case notification rates, and number needed to screen to identify a positive case. We calculated measures of effect and uncertainty in effect estimates using data available in manuscripts where these were not provided by the authors using R 3.61 (R Core Team). Because of high anticipated heterogeneity between and within diseases and interventions investigated, we decided not to undertake meta-analysis.

Ethics statement

This review used published data, and ethical review was not required.

Data sharing statement

The search strategy and summary data tables for this systematic review have all been included in the main text and in the appendix section.

For peer review only

Results

A total of 3919 unique abstracts were identified by the search strategy, from which 3886 were excluded from the title and abstract review, leaving 28 studies that were reviewed as full-text articles. Overall, 10 studies met the inclusion criteria (**Figure S2**). The reasons for exclusion are found in **Table S2**.

One United States of America (USA) based study was identified for HIV. Three TB studies were found: all three studies were from the USA. Three leprosy studies were found: two from Brazil and one study from the Federated States of Micronesia. For malaria, we identified three studies: one each from India, Indonesia and Kenya. The most common study design was implementation demonstration studies (i.e. studies in which spatially targeted interventions were introduced without random allocation of hotspots to study arms). Nine studies had this design: one HIV, three TB, three leprosy and two malaria. We identified one cluster randomised trial (CRT), which evaluated interventions targeted against malaria (**Table S3)**.

Table 1 summarises the characteristics of the included studies, presenting outcomes, measures of intervention effects and study quality/risk of bias assessment results. **Table 2** synthesises methods used to geolocate cases and identify hotspots in the included studies. **Table S3** has details on why studies were excluded following full text review.

Spatially-targeted HIV interventions

In Goswami et al. (29), cases of TB (N=150), HIV (N=665) and syphilis (N=155) notified between 1 Jan 2005 and 31 Dec 2007 in Wake County, North Carolina USA, were geocoded to households. Kernel density maps of the three diseases were generated; maps identified two areas with the densest number of cases (hotspots). Hotspots were defined as areas with more than 10 cases of either TB, HIV or syphilis notified per square mile during the three years. A map of streets and local businesses of Wake County was used to identify the locations of the hotspot areas.

Between 2 June 2009 and 3 Nov 2011, adult community screening for HIV was done in the defined hotspots at specific sites by community nurses and disease intervention specialists from the HIV, syphilis, and TB clinics at the county health department. Of 247 community participants screened by the study, 240 had valid HIV test results. Prevalence of HIV in hotspot areas was compared to that among patients presenting to a sexually transmitted disease (STD) clinic located outside of the hotspot areas. HIV prevalence was higher among community screened participants (8/240, 3%, 95% CI:1.4-6.5) compared to the Wake County STD clinic (64/15936, 0.4%, 95%CI: 0.3-0.5) with a risk ratio of 8.3

For peer review only - http://bmjopen.bmj.com/site/about/guidelines.xhtml

(95% CI: 4.0-17.1, p<0.001). Community HIV screening identified eight HIV positive cases, only one of whom was previously undiagnosed.

Spatially-targeted tuberculosis interventions

In Moonan et al. (24), notified TB cases from 1 Jan 1993 to 31 Dec 2000 (N=991) were geolocated to zip codes in Tarrant County, north-central Texas, USA. Three zip codes were found to have the highest TB notification rates per 100,000 of the population, in addition to having genotypically-clustered *Mycobacterium tuberculosis* isolates (24). The TB notification rates of the zip codes were 94, 55 and 32 cases per 100,000 while the case notification rate for the whole county for the same period was 5.9 cases per 100,000; 95 of 117 (81%) isolates were genotypically clustered in these three zip codes (30). Between 1 Sep 2002 and 31 Dec 2004 community-based organizations offered screening for TB and latent TB infection (LTBI) to hotspot residents. Overall, 3,645 individuals were screened. 1.2% (N = 44) people were diagnosed and treated for active TB, and 18.6% (N=681) were diagnosed and treated for LTBI. This targeted screening identified one person with active TB for every 83 screened, and one person with LTBI for every five screened. The yield of the targeted approach was considered to be more than what would be expected in a county with an active TB notification rate of 5.7 per 100,000 population year.

Goswami et al. (29) (see above in HIV section) also offered screening for LTBI to the same individuals who were offered HIV testing. Both tests were performed from the same blood sample. Hotspot prevalence was compared to LTBI prevalence at the Wake County TB clinic, which was outside of the hotspot areas. Latent TB testing at the TB clinic was offered to high risk individuals who had close contact with a recently diagnosed active TB cases, refugees and those referred by primary care providers or employers. 234/247 had valid LTBI screening results. LTBI prevalence was higher among community screened participants (36/234, 15%, 95% CI:11.0-21.7) versus 6% (95%CI: 5.6-6.6) at the TB clinic with a risk ratio of 2.5 (95% CI: 1.9-3.5, p<0.001).

In Cegielski et al. (31), notified TB cases in Smith County, Texas USA between 1985 to 1995 (N=128) and all notified LTBI cases from 1993 to 1995 (N=311) were geocoded to their households. The geocoded cases were loaded into geographical information systems (GIS) software to produce a point map of both active TB and LTBI cases. The two densest neighbourhoods of TB and LTBI cases were identified visually from the map. In 1996, study field workers went door to door in these neighbourhoods and offered tuberculin skin testing (TST). 1236 /2258 had LTBI testing and received results, 229/1236 (18.5%) were TST positive and 147 received treatment. To assess the intervention, the notified TB cases from 1996 to 2006 in Smith County were mapped to do a before and after intervention comparison. The TB notification rates in the targeted hotspots declined from 39.6 per 100,0000 people per year (95% CI:30.4-48.8) from 1985 to 1995 to zero from 1996 to 2006 (p<0.001).

For peer review only - http://bmjopen.bmj.com/site/about/guidelines.xhtml

While for the entire Smith County the TB notification rates reduced from 8.1 per 100,0000 people per year (95% CI: 5.2-11.0) from 1985 to 1995 to 3.7 per 100,000 people per year (95% CI: 1.2-6.1) from 1996 to 2006 (p<0.001)

Spatially-targeted leprosy interventions

In De Souza Dias et al. (32), leprosy cases notified between 1998 and 2002 (N=368) in the municipality of Mossoro, Rio Grande do Norte in Brazil were geocoded to households. The geocoded cases were used to create a density map with a radius of 100m, and four neighbourhoods with the highest concentration of cases were identified as hotspots. Four active case finding (ACF) campaigns were conducted in these hotspots between March and September 2005. Study team members went door to door to identify people with symptoms of leprosy and referred them to the nearest primary health clinic. 512 possible leprosy cases were referred, and 104 leprosy cases were diagnosed. The cases identified through hotspot active case finding represented 50% of the total cases diagnosed in the city in 2005. In addition, the case notification rate in 2005 was higher than in 2004; 9.34 per 10,000 versus 5.16 per 10,000 respectively.

In Jim et al. (33), in the state of Pohnpei in the Federated States of Micronesia, notified leprosy cases from 2002 to 2006 (N=502) were geocoded to households, producing a point-density map of 1 mile radius; areas with the densest areas were identified for ACF. During 2007 to 2008, ACF teams undertook door to door visits and screened household members for leprosy. There was an eight-fold statewide decrease in the number of households that were screened between 2007 to 2009, while the number of identified cases was similar to the pre-spatially targeted ACF campaign period of 2002 to 2006.

In Barreto et al. (25), notified leprosy cases from the two municipalities of Castanhal and Oriximinal in the state of Para, Brazil from January 2004 to February 2010 (N=633) were geocoded to households. The notified leprosy cases were aggregated by census tract to calculate census case notification rates, and a spatially empirical case detection rate was used to smooth notification rates. Census tracts were classified into four categories, with the highest category assigned to areas with >40 cases per 100,000 population. Kulldorff's spatial scan statistic was used to define groups of hotspot census tracts with statistically higher than average notification rates. Two schools were selected: one within a hotspot and one from a census tract with ≥40 notified leprosy cases per 100,000 populations. At these schools, 134 students with a mean age of 10 years were screened and 11/134, 8.2% (95% CI:3.5-13.0) were diagnosed with leprosy based on clinical signs and symptoms. The diagnostic yield in hotspot area schools was significantly higher than in students from randomly-selected schools from eight

For peer review only - http://bmjopen.bmj.com/site/about/guidelines.xhtml

municipalities in the state of Para in a cross-sectional study between 2009 to 2011: 63/1592, 3.9% (95% CI:3.0-4.9) with a risk ratio of 2.1 (95% CI: 1.1-3.8).

Malaria

In Srivastava et al. (34), notified malaria cases between 2000 to 2005 obtained from the State Department of Health in Madhya Predesh were aggregated at block and district level. Malaria hotspots were defined based on the percentage of malaria cases that had *P. falciparum* malaria of all notified cases from 2000 to 2005. Unspecified targeted malaria interventions in 2007 were evaluated for effectiveness by comparing overall number of confirmed cases notified in 2006 with those notified in 2007. Absolute numbers of notified cases decreased by 5.7% (95%CI:4.7-6.7) from 96,042 in 2006 ($p < 0.05$).

In Herdiana et al. (35), in Sabang island, Indonesia, documented malaria cases from 2007 to 2008 and self-reported malaria cases identified during a survey (N=319), were geocoded to households to produce point maps that classified 14 out of 18 villages as hotspots, based on absolute number of cases. From May 2010, home visits were conducted twice a month in hotspot villages, and only once in non-hotspot villages. Malaria blood smears were taken from anyone with current or recent history of fever, with smear-positive participants referred for treatment. Household contacts and neighbours within 500 meters of smear-positive participants were also screened. Interventions in hotspot areas included: improving malaria diagnostic labs, introduction of Artemisinin-based Combination Therapy (ACT) for malaria treatment, scale-up of indoor residual spraying (IRS) and distribution of long-lasting insecticide-treated nets (LLINs). The incidence of malaria in hotspot areas decreased by 30-fold from 3.18 to 0.13 per 1000 population from 2008 to 2011. (34) (35) (36)

In Bousema et al. (36), between June and July 2011 in Rachuonyo, western Kenya, 17,503 individuals were screened for a malaria prevalence survey, including *P. falciparum* antibodies. SaTScan software was used to define hotspots based on the prevalence of antibody-positivity and age-adjusted antibody density. In total 27 hotspots were identified. A randomised controlled trial randomly allocated ten hotspots 1:1 to either intervention or control. The intervention activities were weekly larviciding of stagnant water bodies, provision of LLINs, IRS and mass drug administration (MDA). MDA was only administered to households that had a confirmed malaria case; febrile individuals (temperature > 37.5^0C) and children aged 6 months – 15 years were offered malaria rapid diagnostic tests (RDT). The following standard interventions were available to both arms, hotspots and evaluation zones (the area 500 metres around each hotspot): annual IRS, case management at health facilities and distribution of LLINs from antenatal clinics.

For peer review only

For peer review only - http://bmjopen.bmj.com/site/about/guidelines.xhtml

There was no statistically significant difference in parasite prevalence in evaluation zones (area around the hotspot) at 8 weeks and 16 weeks post-intervention time points. The first evaluation zone (1-249m) at the 8th week found 3.6% (95% CI: -2.6 to 9.7%, p=0.216) and the second evaluation zone (250-500m) at the 8th week 3.8% (95% CI: -2.4 to 10.0%, p=0.187). Neither was there any significant difference in the first evaluation zone (1-249m) at the 16th week: 1.0%, (95% CI: -7.0 to 9.1%, p=0.713) and the second evaluation zone (250-500m) at 16th week: 1.0% (95% CI: -8.3 to 10.4%, p=0.809).

Influence of study quality on results

The risk of bias assessment of the studies in this review focused on how the hotspots were selected and how the intervention were assessed in each of the studies that were included. The studies in this review were found to have issues that would make them susceptible to risk of bias. Hotspot selection was based mainly on notified cases (24,25,29,32–35,37,38). Relying on notified cases alone can introduce selection bias because notified cases can over-represent cases from areas that have good access to health systems (15).

Further, identified geographical hotspots need to be investigated for stability overtime to ensure that they display spatio-temporal consistency in notification trends (15,39). This was not accounted for in the studies included in this review (24,25,40,29,32–38).

The majority of the identified studies were implementation/pragmatic studies (24,25,29,31–35,40,41). These did not randomise hotspots to interventions and therefore the studies might have compared outcomes of the study in groups of people that had baseline characteristics that were different.

For peer review only

For peer review only - http://bmjopen.bmj.com/site/about/guidelines.xhtml

346 **Table 1: Characteristics of spatially targeted interventions for HIV, TB, Leprosy and Malaria**

**Reference, year**	**Outcome**	**Effect of intervention[1]**	**Risk of bias assessment**
**HIV**			
Goswami 2012 (29)	Comparison of case detection yield between hotspot intervention areas and a county STD clinic over the same period (2009-2011)	HIV prevalence was higher among community screened participants (8/240, 3%, 95% CI:1.4-6.5) compared to the Wake County STD clinic (64/15936, 0.4%, 95%CI:0.3-0.5) with a risk ratio of (8.3, 95% CI: 4.0-17.1), p<0.001.	Moderate
**Tuberculosis**			
Moonan2006 (24)	Yield of TB case detection in hotspots.	Targeted screening identified one person with TB for every 83 screened and one person with LTBI for every five screened. The yield of the targeted approach was considered to be more than what would be expected in a county with an active TB notification rate of 5.7 per 100,000 population year.	Critical
Goswami 2012 (29)	Comparison of case detection yield of LTBI between hotspot areas and county TB clinic over the same period (2009-2011)	LTBI prevalence was higher among community screened participants (36/234, 15%, 95% CI:11.0-21.7) versus (541/9024, 6%, 95%CI=5.6-6.6) at the TB clinic with a risk ratio of (2.5, 95% CI: 1.9-3.5), p<0.001.	Moderate
Cegielski 2013 (31)	A before and after intervention comparison of mapped TB notification rates between 1985-1995 and 1996-2006	TB notification rates in the targeted hotspots declined from 39.6 per 100,0000 people per year (95% CI:30.4-48.8) from 1985 to 1995 to zero from 1996 to 2006 (p<0.001)	Serious
**Leprosy (three studies)**			

For peer review only

De Souza Dias 2007 (32)	Percent of notified cases attributable to the intervention, and before and after intervention case notification rate comparison.	Active case finding identified 50% of the total cases that were diagnosed in 2005. The case notification rate in 2005 was higher compared to pre intervention year 2004, 9.34 per 10000 versus 5.16 per 10000 respectively.	Serious
Jim 2010 (33)	Yield of leprosy case detection during intervention period compared to the pre-intervention period and reduction in households that needed to be screened.	Eight-fold decrease in the number of households that needed to be screened from 2007 to 2009. While still identifying a similar number of new cases to pre-spatially targeted active case finding period 2002 to 2006.	Moderate
Barreto 2015 (25)	The yield of case detection of leprosy cases in school children in hotspot intervention schools versus in children from randomly selected schools.	In the hotspot school's (11/134, 8.2%, 95% CI:3.5-13.0) students with a mean age of 10 years were diagnosed with leprosy. While (63/1592, 3.9%, 95% CI:3.0-4.9) students from randomly selected schools with a mean age of 12 years were diagnosed with leprosy with a risk ratio of (2.1, 95% CI: 1.1-3.8), $p<0.05$.	Moderate
**Malaria (four studies)**			
Srivastava 2009 (34)	Difference in absolute numbers of notified malaria cases between 2006 and 2007.	An absolute reduction in numbers of notified cases in 2007 (N = 90829) from the notified cases in 2006 (N = 96042), (5.7%, 95% CI:4.7-6.7), $p < 0.05$.	Critical
Herdiana2013 (35)	Change of malaria notification rates from pre-intervention to post-intervention period.	30-fold reduction in malaria notifications from 3.83 per 1000 in 2008 to 0.13 per 1000 in 2011.	Serious
Bousema2016 (36)	Change in parasite prevalence in the evaluation zones (1-500m from hotspots) of intervention clusters versus control clusters	The first evaluation zone 1-249m at 8th week (3.6%, 95% CI: -2.6 to 9.7%), p=0.216 and the second evaluation zone 250-500m at 8th week (3.8%, 95% CI: -2.4 to 10.0%), p=0.187. The first evaluation zone 1-249m at 16th	Low

For peer review only - http://bmjopen.bmj.com/site/about/guidelines.xhtml

		week (1.0%, 95% CI: -7.0 to 9.1%), p=0.713 and the second evaluation zone 250-500m at 16th week (1.0%, 95% CI: -8.3 to 10.4%), p=0.809	

[1] Results calculated from the data published in the papers.

Abbreviations: TB Tuberculosis, LTBI Latent Tuberculosis Infection, m Metres, N Number, STD Sexually Transmitted Disease.

For peer review only

349 **Table2: Geolocation of cases and hotspot identification**

Reference, year	Geolocation of cases	Hotspot identification
**HIV (one study)**		
Goswami2012 (29)	Notified cases of either Tuberculosis(TB) (N=150), HIV (N=665) or syphilis (155) between 1 Jan 2005 and 31 Dec 2007 were geolocated to households. The method for geolocation of the cases was not described in the paper.	A kernel density map of the cases was produced. Areas with the highest densities of three diseases of HIV, Syphilis and TB (greater than 10 cases per square mile) were classified as hotspots. Two hotspot neighbourhoods were identified in the county.
**Tuberculosis (four studies)**		
Moonan, 2006 (24)	Notified TB cases (N=991) from 1 Jan 1993 to 31 Dec 2000 in Tarrant County, north central Texas, USA were geolocated to zip codes using residential addresses and zip codes that patients gave at the time of diagnosis with the aid of a GIS software.	Areas with the highest TB notification rates and high percentage of genotypically clustered TB isolates were identified as hotspots. Three neighbourhood hotspots were identified.
Goswami2012 (29)	Notified cases of TB (N=150), HIV (N=665) and syphilis (N= 155) that were notified between 1 Jan 2005 and 31 Dec 2007 were geolocated to households. The method for geolocation of the households was not described in the paper	A kernel density map was developed. Areas with the highest densities of three diseases of HIV, Syphilis and TB (greater than 10 cases per square mile) were classified as hotspots. Two hotspot neighbourhoods were identified in the county.
Cegielski 2013 (31)	Notified TB cases between 1985 to 1995 (N=128) and all notified LTBI from 1993 to 1995 (N=311) were geocoded to their households using the addresses that patients gave at the time of diagnosis. In addition, field workers tracked addresses to households to get household coordinates of addresses that failed to geolocate.	The points of cases were plotted on a map and areas with the densest clusters of points of cases were identified as hotspots, 2 neighbourhoods were identified in the county.

For peer review only - http://bmjopen.bmj.com/site/about/guidelines.xhtml

**Leprosy (three studies)**		
DE Souza Dias2007 (32)	Notified leprosy cases that occurred between 1998 and 2002 (N=368) in the municipality of geocoded to households. The method for geolocation of cases was not described.	Density map with a radius of 100m of the notified leprosy cases was produced. Four hotspot areas were identified
Jim2010(33)	Notified leprosy cases from 2002 to 2006 (N=502) were geolocated to households. Field workers visited all notified cases to get household GPS coordinates using a GIS device.	A density map based on 1-mile radius of the notified leprosy cases. Areas with high concentration of cases classified as hotspots.
Barreto2015 (25)	Notified leprosy cases from January 2004 to February 2010 (n=633) were geocoded to households. Field workers visited households of registered cases to collect GPS coordinates.	Hotspots were identified using the Kulldorff's spatial scan statistic and by stratification of the leprosy notified rates. Two hotspots were identified.
**Malaria (four studies)**		
Srivastava2009 (34)	Notified malaria cases between 2000 to 2005 were obtained from the State Department of Health based on the cases notified in clinics in the blocks or districts.	Blocks or districts with a percentage of plasmodium falciparum malaria cases of all notified cases that was either 100% or consistently greater than 30% from 2000-2005 or greater than 70% in 2005
Herdiana2013 (35)	Notified malaria cases from 2007 to 2008 in addition to other self-reported malaria cases that were found during a survey (n=319) were geocoded to households. Field workers obtained the GPS coordinates of households using GIS devices.	Villages that had the majority of malaria cases were classified as hotspots. 14 out of 18 villages were identified
Bousema2016 (36)	June and July 2011, 17,503 individuals tested in a malaria prevalence survey for the prevalence of P. falciparum antibodies (AMA-1 or MSP-10). Field workers collected the GPS coordinates of the households using GPS devices.	Segments of the study area were scanned in the 2 x 4 km rolling windows and areas with higher ($p < 0.05$) prevalence of antibodies and age-adjusted antibody density than the local average values were identified as hotspots.

350 Abbreviations: GIS Geographical Information System, GPS Global Positioning System, HIV Human Immunodeficiency Virus, m Metres, N Number, TB Tuberculosis, STD
351 Sexually Transmitted Disease.

For peer review only

Discussion

Main findings

The key finding of this review is that, despite increasing enthusiasm for the application of spatially targeted interventions in public health research addressing infectious diseases (42), very few studies have rigorously evaluated the effectiveness of such approaches against non-hotspot comparator areas. We identified only 10 studies conducted since 1993, nearly all of which had substantial limitations in how hotspots were defined and identified, how spatially-targeted interventions were evaluated, or how comparator areas were selected. With the limited evidence available, we found some suggestion that hotspot-targeted interventions for tuberculosis, malaria and leprosy may be efficient and effective approaches in increasing diagnostic yield, reducing unnecessary screening, and perhaps in improving disease epidemiology. However, almost all the studies were vulnerable to bias due to regression to the mean: hotspots identified by highest prevalence/incidence will typically see prevalence/incidence decline, even in the absence of an intervention. As such, spatially-targeted approaches hold promise, but require further evaluation in high-quality studies to guide policymakers considering implementing this approach.

Strengths and limitations

We found fewer studies than we anticipated, potentially due to the challenges of collecting spatial data in developing countries where most areas do not yet have municipal address systems or subdistrict postal code systems (15,43). Nevertheless, in recent years technologies for obtaining spatial data have improved, become cheaper and hence more widely available; consequently, the inclusion of spatial data within epidemiological and surveillance studies as a tool to identify disease hotspots has become more common (42). In theory, this should enable disease control programmes to collect spatial data for identifying and targeting hotspots (42)(43). Thus, in future, prioritising complete mapping of low income countries, with introduction of effective residential identification systems should be a major priority. This would allow some of the methods for geolocation of notified cases in the studies in this review, which used manual and labour intensive approaches that also had high potential for imprecision, to be replaced with more efficient approaches (33).

Investment in national geospatial and surveillance tools that allow rapid, accurate and scalable geolocation of cases from within health facilities in settings that lack postal codes would greatly facilitate spatial mapping of areas of interest (44). Such tools would enable the identification of hotspots at finer scale and limit the use of arbitrary blocks (34). One important concern about targeting interventions on the basis of case-notifications alone, however, would be the potential for ascertainment bias to further disadvantage underserved populations from which cases are already

For peer review only

under-notified due to limited access to health services (45). For populations where this is likely, the ideal approach would be to investigate disease burden using prevalence surveys, or developing models that include data on geographical distribution of measures of poverty and health service access that can be then allow routine notification data to be adjusted to account for likely under-ascertainment (45).

We report a wide variety of interventions in the studies included in this systematic review. For HIV (29), TB (24,29,31) and leprosy (25,32,33), studies tended to focus on community screening interventions in suspected geographic hotspots. Malaria studies (34–36), however, were more focused on complex interventions that combined community screening and mass drug administration, with environmental measures to address the vector, including larviciding of stagnant water bodies and indoor residual spraying.

Despite the above reservations, the studies that we have identified demonstrate the feasibility of the spatially targeted approach to direct resources towards community-based interventions. For case-finding interventions or community screening, spatially targeted interventions are a compromise between tracing known contacts of infectious cases and active case finding interventions across the entire general populations or sub-population (i.e. whole urban slums) (46). One limitation of contact tracing is that it misses out cases that are unknown to the index case, and so will fail to identify cases arising from transmission to casual contacts outside of the household (47,48). Spatially targeted interventions become increasingly important for diseases that are approaching elimination, since these conditions predispose the disease cases to cluster into disease hotspots (13–19) . Hence spatially targeted interventions can help the most successful control programmes to systematically identify and address residual disease hotspots (12,19,32,49).Among all of the studies reviewed here, only one (Bousema et al.) (36) was assessed as having low risk of bias. This was a malaria intervention that evaluated the impact of a spatially targeted intervention in pre-defined inner and outer regions of hotspots (evaluation zones). This study design allows the evaluation of how targeting the hotspots benefits the individuals that are in the hotspot as well as the individuals that are in the outer regions of the hotspot. This in turn allows assessment of the impact of the intervention on reducing transmission. For vector borne diseases, including malaria, consideration need to be given to the mobility of the vectors themselves when considering spatially targeted interventions (50) i.e. if mosquitoes are able to travel over moderate distances the effect of a hotspot targeted intervention might end up being diluted.

However, this study had several limitations (36). It was underpowered to detect small changes due to the hotspot targeted intervention. In addition, the intervention was also done in one transmission

For peer review only - http://bmjopen.bmj.com/site/about/guidelines.xhtml

season which might not have been enough to interrupt the epidemic (36). The area of study might also not have been suitable for a hotspot targeted intervention because even though malaria was heterogeneous there might have been widespread transmission of malaria going on in the non-hotspot areas; this might have meant that a non-targeted approach would have been more appropriate for this setting (51).

Overall, our systematic review's limitation was that the studies we identified were heterogeneous, and we were unable to do a meta-analysis as planned. Also, the studies' methodological quality had challenges due to the lack of validation of disease hotspots and the lack of random allocation of clusters to interventions. We also only included manuscripts in English due to resource limitations, and only four diseases were included. Despite these limitations, we were able to show the potential that spatially targeted interventions have towards improving disease epidemiology.

Recommendations

In general spatially-targeted interventions are only justifiable in settings where the epidemic is heterogenous and secondly where the non-hotspot areas cannot sustain sufficient transmission of the disease (36). The influence of the hotspots on the epidemic of the disease in the surrounding communities also depends on the effective contact rates between the residents of the hotspots and the surrounding areas; in the case of malaria this also depends on how far mosquitoes can travel between the hotspots and the surrounding communities. Careful attention needs also to be given to identifying hotspots to make sure that the hotspots capture the true burden of the disease by making sure that the hotspots are persistent in time and are adjusted for confounding (50–52).

Conclusion

The rapid increase in cheap, reliable, geolocation technology now provides such a significant tool in the fight to control and eradicate endemic and epidemic infectious diseases that support for rapid development of effective patient mapping systems should be considered essential for health systems in low income countries. Ideally, mapping should be combined with statistical modelling to identify hotspots with the most pressing need for intervention. In this systematic review we provide some evidence in support of the effectiveness of spatially targeted intervention, but with the major conclusion being that the current body of evidence is weak. There is an urgent need to define optimal methodology, allowing recommendations to be made to support the design of more rigorous studies that allow clear evaluation of likely impact to be made for the major categories of infectious diseases, including vector-borne and those transmitted person-to-person. Recommendations should include

For peer review only - http://bmjopen.bmj.com/site/about/guidelines.xhtml

449 strategies for timely identification of hotspots and the critical aspects needed to measure the effect

450 of intervention strategies targeting those hotspots.

For peer review only

For peer review only - http://bmjopen.bmj.com/site/about/guidelines.xhtml

Declarations

Ethics approval and consent to participate

This review used published data, and ethical review was not required.

Consent for publication

Not applicable

Availability of data and materials

The search strategy and summary data tables for this systematic review have all been included in the main text and in the appendix section.

Competing interests

There are no conflicts of interest to declare.

Funding

This work was supported by Overseas Collaborative Site Staff-Studentship included as part of Professor Elizabeth Corbett's (ELC) Senior Research Fellowship in Clinical Science from Wellcome Trust ("Sustainable Community Action for Lung hEalth (SCALE): a cluster-randomised trial in Blantyre, Malawi." WT200901/Z/16/Z.). The funders had no role in study design; in the collection, analysis, and interpretation of data; in the writing of the report; and in the decision to submit the paper for publication. Peter MacPherson (PM) is funded by Wellcome Trust (206575)

Authors' contributions

MK, PM, ELC, and James Carpenter (JC) designed the study. MK, PM, JC and MN contributed to the data collection and management. MK, PM, JC and MN contributed to the data analysis. MK, PM, ELC, JC, Ted Cohen (TC) and MN contributed to the data interpretation. MK wrote the first draft. MK, PM, ELC, JC, TC and MN reviewed, contributed to, and approved the final draft. All authors read and approved the final manuscript

Acknowledgements

We would like to thank the LSHTM library staff for assisting us with the formulation of the search strategy.

For peer review only

For peer review only - http://bmjopen.bmj.com/site/about/guidelines.xhtml

References

1. United Nations Joint Programme on HIV/AIDS (UNAIDS). UNAIDS data 2018 [Internet]. Geneva; 2018. Available from: http://www.unaids.org/sites/default/files/media_asset/unaids-data-2018_en.pdf
2. World Health Organisation. Global tuberculosis report 2018. Geneva; 2018.
3. World Health Oganisation. Weekly leprosy epidemiological report 31AUG2018 [Internet]. Geneva; 2018 [cited 2019 Feb 1]. Available from: http://apps.who.int/iris/bitstream/hand
4. World Health Organisation. World malaria report 2018 [Internet]. Geneva; 2018 [cited 2019 Feb 1]. Available from: www.who.int/malaria
5. World Health Organisation. Global leprosy strategy 2016-2020. Geneva; 2015.
6. World Health Organisation. Frequently asked questions on leprosy [Internet]. Geneva; 2013. Available from: http://apps.searo.who.int/PDS_DOCS/B5044.pdf
7. Almeida ACG, Kuehn A, Castro AJM, Vitor-Silva S, Figueiredo EFG, Brasil LW, et al. High proportions of asymptomatic and submicroscopic Plasmodium vivax infections in a peri-urban area of low transmission in the Brazilian Amazon. Parasit Vectors [Internet]. 2018 Dec 20 [cited 2019 Feb 1];11(1):194. Available from: http://www.ncbi.nlm.nih.gov/pubmed/29558985
8. Ngwira LG, Dowdy DW, Khundi M, Barnes GL, Nkhoma A, Choko AT, et al. Delay in seeking care for tuberculosis symptoms among adults newly diagnosed with HIV in rural Malawi. INT J TUBERC LUNG DIS [Internet]. 2018 Mar 1 [cited 2019 Feb 1];22(3):280–6. Available from: http://dx.doi.org/10.5588/ijtld.17.0539
9. Fomundam HN, Tesfay AR, Mushipe SA, Mosina MB, Boshielo CT, Nyambi HT, et al. Prevalence and predictors of late presentation for HIV care in South Africa. S Afr Med J [Internet]. 2017 Nov 27 [cited 2019 Feb 1];107(12):1058–64. Available from: http://www.ncbi.nlm.nih.gov/pubmed/29262956
10. Sulat JS, Prabandari YS, Sanusi R, Hapsari ED, Santoso B. The impacts of community-based HIV testing and counselling on testing uptake. J Heal Res [Internet]. 2018;JHR-01-2018-015. Available from: http://www.emeraldinsight.com/doi/10.1108/JHR-01-2018-015
11. Ho J, Fox GJ, Marais BJ. Passive case finding for tuberculosis is not enough. Int J Mycobacteriology [Internet]. 2016 Dec 1 [cited 2019 Mar 7];5(4):374–8. Available from: https://www.sciencedirect.com/science/article/pii/S2212553116301595?via%3Dihub
12. World Health Organisation. A framework for malaria elimination global Malaria programme [Internet]. Geneva; 2017 [cited 2019 Mar 13]. Available from: https://apps.who.int/iris/bitstream/handle/10665/254761/9789241511988-

For peer review only - http://bmjopen.bmj.com/site/about/guidelines.xhtml

eng.pdf;jsessionid=FEBA2DD0A83E29D8833B238DAD060E96?sequence=1

13. Brian J. Coburn, Justin T. Okano SB, Coburn BJ, Okano JT, Blower S. Using geospatial mapping to design HIV elimination strategies for sub-Saharan Africa. Sci Transl Med [Internet]. 2017;9(383 PG-). Available from: NS -

14. Cuadros DF, Li J, Branscum AJ, Akullian A, Jia P, Mziray EN, et al. Mapping the spatial variability of HIV infection in Sub-Saharan Africa: Effective information for localized HIV prevention and control. Sci Rep. 2017;7(1):1–11.

15. Shaweno D, Karmakar M, Alene KA, Ragonnet R, Clements AC, Trauer JM, et al. Methods used in the spatial analysis of tuberculosis epidemiology: a systematic review. BMC Med [Internet]. 2018 [cited 2019 Feb 20];16(1):193. Available from: https://bmcmedicine.biomedcentral.com/articles/10.1186/s12916-018-1178-4

16. Monteiro LD, Martins-Melo FR, Brito AL, Alencar CH, Heukelbach J. Spatial patterns of leprosy in a hyperendemic state in Northern Brazil, 2001-2012. Rev Saude Publica. 2015;49.

17. Alencar CH, Ramos AN, dos Santos ES, Richter J, Heukelbach J. Clusters of leprosy transmission and of late diagnosis in a highly endemic area in Brazil: Focus on different spatial analysis approaches. Trop Med Int Heal. 2012;17(4):518–25.

18. Sluydts V, Heng S, Coosemans M, Van Roey K, Gryseels C, Canier L, et al. Spatial clustering and risk factors of malaria infections in Ratanakiri Province, Cambodia. Malar J [Internet]. 2014 Dec 30 [cited 2019 Feb 1];13(101139802):387. Available from: http://ovidsp.ovid.com/ovidweb.cgi?T=JS&PAGE=reference&D=med10&NEWS=N&AN=25269827

19. Shaweno D, Trauer JM, Denholm JT, McBryde ES. The role of geospatial hotspots in the spatial spread of tuberculosis in rural Ethiopia: a mathematical model. R Soc open Sci [Internet]. 2018;5(9):180887. Available from: http://ovidsp.ovid.com/ovidweb.cgi?T=JS&PAGE=reference&D=prem&NEWS=N&AN=30839742

20. Lessler J, Azman AS, McKay HS, Moore SM. What is a Hotspot Anyway? Am J Trop Med Hyg [Internet]. 2017 Jun [cited 2019 Feb 18];96(6):1270–3. Available from: http://www.ncbi.nlm.nih.gov/pubmed/28719289

21. Shrestha S, Chatterjee S, Rao KD, Dowdy DW. Potential impact of spatially targeted adult tuberculosis vaccine in Gujarat, India. J R Soc Interface [Internet]. 2016;13(116). Available from: http://ovidsp.ovid.com/ovidweb.cgi?T=JS&PAGE=reference&D=med12&NEWS=N&AN=27009179

For peer review only

22. Lonnroth, Knut. Systematic screening for active tuberculosis Principles and recommendations [Internet]. [cited 2019 May 27]. Available from: https://www.who.int/tb/publications/Final_TB_Screening_guidelines.pdf

23. United Nations Human Settlements Programme. SLUM ALMANAC 2015 2016 Tracking Improvement in the Lives of Slum Dweller [Internet]. Nairobi; 2015. Available from: https://unhabitat.org/slum-almanac-2015-2016/

24. Moonan PK, Oppong J, Sahbazian B, Singh KP, Sandhu R, Drewyer G, et al. What is the outcome of targeted tuberculosis screening based on universal genotyping and location? Am J Respir Crit Care Med [Internet]. 2006;174(5):599–604. Available from: http://ovidsp.ovid.com/ovidweb.cgi?T=JS&PAGE=reference&D=med5&NEWS=N&AN=16728707

25. Barreto JG, Bisanzio D, Frade MAC, Moraes TMP, Gobbo AR, Guimarães L de S, et al. Spatial epidemiology and serologic cohorts increase the early detection of leprosy. BMC Infect Dis [Internet]. 2015 Nov 16 [cited 2019 Mar 12];15(1):1–9. Available from: http://www.ncbi.nlm.nih.gov/pubmed/26573912

26. Wold Health Organisation. Systematic screening for active tuberculosis. Geneva; 2013.

27. Higgins JPT, Altman DG, Gøtzsche PC, Jüni P, Moher D, Oxman AD, et al. The Cochrane Collaboration's tool for assessing risk of bias in randomised trials. BMJ. 2011 Oct 29;343(7829).

28. Sterne JA, Hernán MA, Reeves BC, Savović J, Berkman ND, Viswanathan M, et al. ROBINS-I: A tool for assessing risk of bias in non-randomised studies of interventions. BMJ. 2016 Oct 12;355.

29. Goswami ND, Hecker EJ, Vickery C, Ahearn MA, Cox GM, Holland DP, et al. Geographic information system-based screening for TB, HIV, and syphilis (GIS-THIS): a cross-sectional study. Pai M, editor. PLoS One [Internet]. 2012 Oct 2 [cited 2019 Mar 29];7(10):e46029. Available from: http://ovidsp.ovid.com/ovidweb.cgi?T=JS&PAGE=reference&D=med8&NEWS=N&AN=23056227

30. Moonan PK, Bayona M, Quitugua TN, Oppong J, Dunbar D, Jost KCJ, et al. Using GIS technology to identify areas of tuberculosis transmission and incidence. Int J Health Geogr [Internet]. 2004;3(1):23. Available from: http://ovidsp.ovid.com/ovidweb.cgi?T=JS&PAGE=reference&D=medp&NEWS=N&AN=15479478

31. Cegielski JP, Griffith DE, McGaha PK, Wolfgang M, Robinson CB, Clark PA, et al. Eliminating

For peer review only

For peer review only - http://bmjopen.bmj.com/site/about/guidelines.xhtml

tuberculosis one neighborhood at a time. Am J Public Health [Internet]. 2013 Jul [cited 2019 Feb 5];103(7):1292–300. Available from: http://www.ncbi.nlm.nih.gov/pubmed/23078465

32. De Souza Dias MCF, Dias GH, Nobre ML, M.C.F. DSD, G.H. D, M.L. N. The use of Geographical Information System (GIS) to improve active leprosy case finding campaigns in the Municipality of Mossoro, Rio Grande do Norte State, Brazil. Lepr Rev [Internet]. 2007;78(3):261–9. Available from: http://www.lepra.org.uk/lr/Sept07/Lep261-269.pdf

33. Jim R, Johnson E, Pavlin BI. Role of GIS technology during leprosy elimination efforts in Pohnpei. Pac Health Dialog [Internet]. 2010;16(1):109–14. Available from: http://ovidsp.ovid.com/ovidweb.cgi?T=JS&PAGE=reference&D=med7&NEWS=N&AN=20968242

34. Srivastava A, Nagpal BN, Joshi PL, Paliwal JC, Dash AP, A. S, et al. Identification of malaria hot spots for focused intervention in tribal state of India: a GIS based approach. Int J Health Geogr [Internet]. 2009;8(101152198):30. Available from: http://ovidsp.ovid.com/ovidweb.cgi?T=JS&PAGE=reference&D=med6&NEWS=N&AN=19457227

35. Herdiana H, Fuad A, Asih PB, Zubaedah S, Arisanti RR, Syafruddin D, et al. Progress toward malaria elimination in sabang, aceh, indonesia. Am J Trop Med Hyg [Internet]. 2012;87(5 SUPPL. 1):47. Available from: http://www.ajtmh.org/content/87/5_Suppl_1/1.1.full.pdf+html

36. Bousema T, Stresman G, Baidjoe AY, Bradley J, Knight P, Stone W, et al. The Impact of Hotspot-Targeted Interventions on Malaria Transmission in Rachuonyo South District in the Western Kenyan Highlands: A Cluster-Randomized Controlled Trial. PLoS Med. 2016;13(4):e1001993–e1001993.

37. Tripepi G, Jager KJ, Dekker FW, Zoccali C. Selection bias and information bias in clinical research. Nephron - Clin Pract. 2010;115(2).

38. Morris U, Msellem MI, Mkali H, Islam A, Aydin-Schmidt B, Jovel I, et al. A cluster randomised controlled trial of two rounds of mass drug administration in Zanzibar, a malaria pre-elimination setting-high coverage and safety, but no significant impact on transmission. BMC Med [Internet]. 2018;16(1):215. Available from: http://ovidsp.ovid.com/ovidweb.cgi?T=JS&PAGE=reference&D=medl&NEWS=N&AN=30526588

39. Mosha JF, Sturrock HJW, Greenwood B, Sutherland CJ, Gadalla NB, Atwal S, et al. Hot spot or not: a comparison of spatial statistical methods to predict prospective malaria infections. Malar J [Internet]. 2014;13(101139802):53. Available from: http://ovidsp.ovid.com/ovidweb.cgi?T=JS&PAGE=reference&D=med10&NEWS=N&AN=24517

For peer review only - http://bmjopen.bmj.com/site/about/guidelines.xhtml

452

40. Hsiang MS, Ntshalintshali N, Kang Dufour M-S, Dlamini N, Nhlabathi N, Vilakati S, et al. Active case-finding for malaria: A three-year national evaluation of optimal approaches to detect infections and hotspots through reactive case detection in the low transmission setting of Eswatini. Clin Infect Dis [Internet]. 2019; Available from: http://ovidsp.ovid.com/ovidweb.cgi?T=JS&PAGE=reference&D=medp&NEWS=N&AN=31095677

41. Fatima R, Qadeer E, Yaqoob A, Haq MU, Majumdar SS, Shewade HD, et al. Extending "Contact Tracing" into the Community within a 50-Metre Radius of an Index Tuberculosis Patient Using Xpert MTB/RIF in Urban, Pakistan: Did It Increase Case Detection?. PLoS One [Internet]. 2016;11(11):e0165813. Available from: http://ovidsp.ovid.com/ovidweb.cgi?T=JS&PAGE=reference&D=med12&NEWS=N&AN=27898665

42. Elliott P, Wartenberg D. Spatial epidemiology: Current approaches and future challenges. Vol. 112, Environmental Health Perspectives. Public Health Services, US Dept of Health and Human Services; 2004. p. 998–1006.

43. Dowdy DW, Dye C, Cohen T. Data Needs for Evidence-Based Decisions: A TB Modeler's "Wish List" [cited 2019 Feb 25]; Available from: https://www.ncbi.nlm.nih.gov/pmc/articles/PMC4041555/pdf/nihms584157.pdf

44. MacPherson P, Khundi M, Nliwasa M, Choko AT, Phiri VK, Webb EL, et al. Disparities in access to diagnosis and care in Blantyre, Malawi, identified through enhanced tuberculosis surveillance and spatial analysis. BMC Med [Internet]. 2019 Dec 29 [cited 2019 Feb 6];17(1):21. Available from: https://bmcmedicine.biomedcentral.com/articles/10.1186/s12916-019-1260-6

45. Borgdorff MW. New measurable indicator for tuberculosis case detection. Emerg Infect Dis [Internet]. 2004 Sep [cited 2019 Feb 6];10(9):1523–8. Available from: http://www.ncbi.nlm.nih.gov/pubmed/15498151

46. Department of HIV/AIDS, Stop TB Department, World Health Organization (WHO). Guidelines for intensified tuberculosis case-finding and isoniazid preventive therapy for people living with HIV in resource-constrained settings [Internet]. Geneva; 20111 [cited 2019 May 24]. Available from: https://apps.who.int/iris/bitstream/handle/10665/44472/9789241500708_eng.pdf;jsessionid=21FF5A6C6174558D73F38D25777B56F0?sequence=1

47. Crampin AC, Glynn JR, Traore H, Yates MD, Mwaungulu L, Mwenebabu M, et al. Tuberculosis

For peer review only

For peer review only - http://bmjopen.bmj.com/site/about/guidelines.xhtml

transmission attributable to close contacts and HIV status, Malawi. Emerg Infect Dis. 2006;12(5):729–35.

48. Chamie G, Kato-Maeda M, Emperador DM, Wandera B, Mugagga O, Crandall J, et al. Spatial overlap links seemingly unconnected genotype-matched TB cases in rural Uganda. 2018 [cited 2019 Jan 30]; Available from: https://doi.org/10.1371/journal.pone.0192666

49. Busgeeth K, Rivett U. The use of a spatial information system in the management of HIV/AIDS in South Africa. Int J Health Geogr [Internet]. 2004;3(13). Available from: http://www.ij-healthgeographics.com/content/3/1/13/abstract

50. Bousema T, Griffin JT, Sauerwein RW, Smith DL, Churcher TS, Takken W, et al. Hitting hotspots: spatial targeting of malaria for control and elimination. PLoS Med [Internet]. 2012 Jan [cited 2019 Mar 14];9(1):e1001165. Available from: http://www.ncbi.nlm.nih.gov/pubmed/22303287

51. Stresman G, Bousema T, Cook J. Malaria Hotspots: Is There Epidemiological Evidence for Fine-Scale Spatial Targeting of Interventions? Vol. 35, Trends in Parasitology. Elsevier Ltd; 2019. p. 822–34.

52. World Health Organisation. WHO consolidated guidelines on tuberculosis - Module 2: Screening - Systematic screening for tuberculosis disease - World | ReliefWeb [Internet]. 2021 [cited 2021 Apr 6]. Available from: https://reliefweb.int/report/world/who-consolidated-guidelines-tuberculosis-module-2-screening-systematic-screening

For peer review only

For peer review only - http://bmjopen.bmj.com/site/about/guidelines.xhtml

Appendix 1

Figure S1: Research Overview

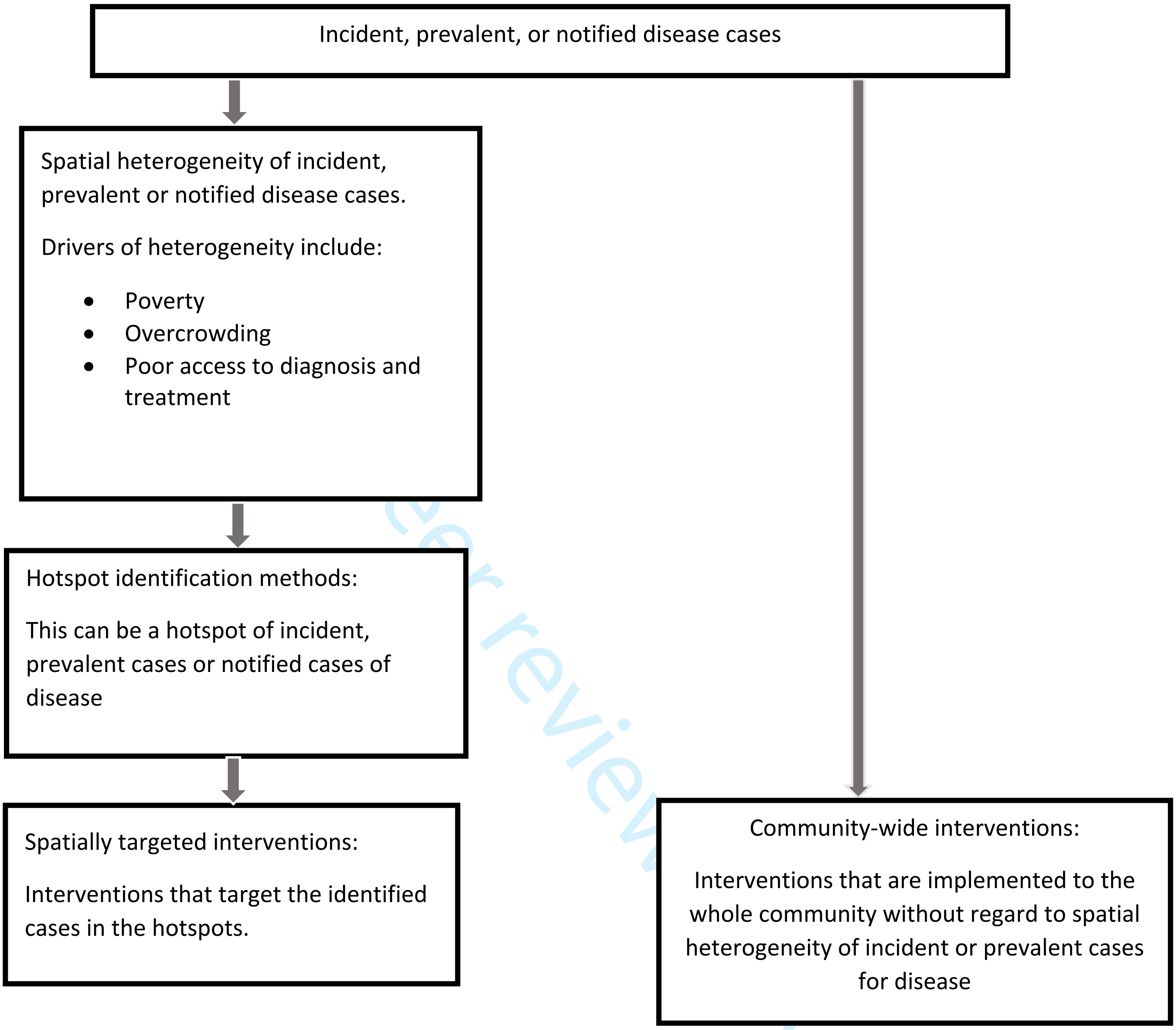

For peer review only - http://bmjopen.bmj.com/site/about/guidelines.xhtml

Figure S2: PRISMA flow diagram for the process of selecting included studies

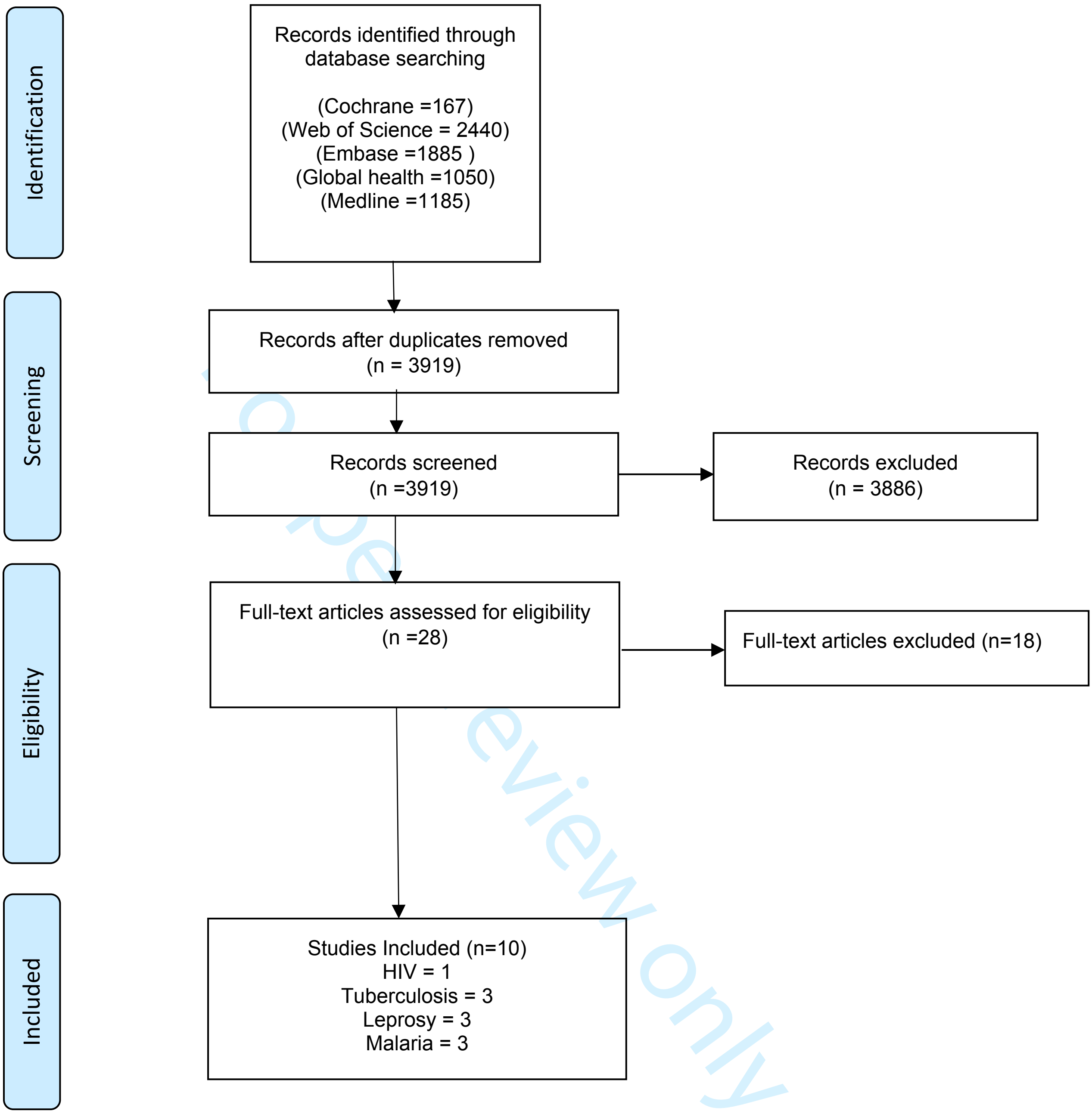

For peer review only

Table S1: Search Strategy

1	(HIV or human immunodeficiency virus or HIV-1 or HIV-2).mp.
2	exp HIV/
3	exp HIV Infections/
4	(aids or acquired immune deficiency syndrome).mp.
5	exp Acquired Immunodeficiency Syndrome/
6	(tuberculosis or TB).mp.
7	exp Tuberculosis/
8	exp Mycobacterium tuberculosis/
9	(malaria* or Plasmodium).mp.
10	exp Malaria/
11	exp Plasmodium/
12	(Leprosy or lepra or lepromatosis or hanseniase).mp.
13	exp Leprosy/
14	exp Mycobacterium leprae/
15	(spatial adj3 (analys?s or regression or temporal or autocorrelation* or auto-correlation* or statistics or epidemiology)).mp.
16	exp spatial analysis/
17	(geographic* adj3 (analys?s or regression or temporal or autocorrelation* or auto-correlation* or system* or epidemiology)).mp.
18	(GPS or global position* system* or GIS or global information system* or space?time or geospatial or hotspot* or hot-spot*).mp.
19	exp Geographic Information Systems/
20	(communit* or neighbo?rhood*).mp.
21	exp Residence Characteristics/
22	(intervention* or target*).mp.
23	((high-burden or highburden) adj3 (area* or region* or tract* or setting*)).mp.
24	(high-incidence adj3 (area* or region* or tract* or setting*)).mp.
25	(high-prevalence adj3 (area* or region* or tract* or setting*)).mp.
26	((hot-spot* or hotspot*) adj3 (area* or region* or tract*)).mp
27	1 or 2 or 3 or 4 or 5 or 6 or 7 or 8 or 9 or 10 or 11 or 12 or 13 or 14
28	15 or 16 or 17 or 18 or 19
29	20 or 21 or 22 or 23 or 24 or 25 or 26
30	27 and 28 and 29
31	limit 30 to english language
32	limit 31 to yr="1993 -Current"

For peer review only

Table S2: Reasons for exclusion of studies

**Reference, year**	**Reason for excluding**
**HIV (three studies)**	
Odek 2014 (1)	HIV hotspots not based on geolocated cases
Ikpeazu 2014 (2)	HIV hotspots not based on geolocated cases
Mburu 2017 (3)	HIV hotspots not based on geolocated cases
**Tuberculosis (four studies)**	
Clark 1999 (4)	Review
Wilkinson 1999 (5)	No hotspots based on geolocated TB cases.
Fatima 2016 (6)	Contact tracing study
Vo 2020 (7)	Hotspots were not based on TB cases
**Malaria (eleven studies)**	
Mnzava 2001 (8)	Cluster randomised trial with no standard of care arm.
Ghosh 2007 (9)	Review
Dongus 2007 (10)	Hotspot not based on geolocated malaria cases
Cook 2007 (11)	No spatially targeted intervention was evaluated
Shiff 2012 (12)	No spatially targeted intervention was evaluated
Marston 2014 (13)	Health economics study
Cook 2014 (14)	Hotspot not based on geolocated malaria cases
Hsiang 2019 (15)	Defining optimal strategies for contact tracing strategies
Ousmane 2019 (16)	No clear definition of hotspot and study evaluation not based on hotspots.
Ngo 2019 (17)	Feasibility study of acceptability of geographic information technology
Bhondoekhan 2020 (18)	Contact tracing study

For peer review only

Table S3: Full description of the characteristics of spatially targeted interventions of HIV, TB, Leprosy and Malaria

Reference, year	Setting	Study type	Intervention	Control
**HIV (one study)**				
Goswami2012(19)	Wake County, North Carolina USA	Implementation study	Community screening of HIV at sites in the hotspot areas by community nurses and disease intervention specialists from the HIV, syphilis and TB clinics at the county health department.	HIV screening of patients going to STD clinic outside of hotspot areas in the same county.
**Tuberculosis (three studies)**				
Moonan, 2006(20)	Tarrant County, north central Texas, USA	Implementation study	Community tuberculin skin testing and active TB screening by community organisations in hotspots (period 2002-2004)	No control or comparison group.
Goswami2012(19)	Wake County, North Carolina USA	Implementation study	Community screening of latent TB at sites in the hotspot areas by community nurses and disease intervention specialists from the HIV, Syphilis and TB clinics at the county health department.	Screening latent TB in patients going to a TB clinic outside of hotspot areas in the same county.
Cegielski2013 (21)	Smith County, Texas USA	Implementation study	Community screening of tuberculin skin testing and treatment of diagnosed in the hotspots (1996)	No control or comparison group
**Leprosy (three studies)**				

For peer review only

DE Souza Dias2007(22)	Municipality of Mossoro, Rio Grande do Norte, Brazil	Implementation study	Study team members moved door to door in the hotspots to identify people with symptoms of leprosy and referred them to the nearest primary health clinic for diagnosis (period 2005)	No control group
Jim2010(23)	state of Pohnpei in the Federated State of Micronesia.	Implementation study	Door to door community Screening of Leprosy cases in the hotspots (2007 to 2009)	No control group
Barreto2015(24)	Municipalities of Castanhal and Oriximinal in the state of Para, Brazil	Implementation study	Community leprosy case screening in two public schools located in the hotspot	Prevalence of leprosy in children from randomly selected schools not from the hotspot areas.
**Malaria (three studies)**				
Srivastava2009(25)	central Indian state of Madhya Predesh	Implementation study	Not specifically listed. (2007)	No control

For peer review only

Herdiana2013(26)	Island district of Sabang in Indonesia	Implementation study	Twice a month visit households. At each visit screen for malaria from household members with fever or recent history of fever. Treat all confirmed malaria cases.(May 2010 onwards)	Once a month to households in the hotspots. At each visit screen for malaria from household members with fever or recent history of fever. Treat all confirmed malaria cases. (May 2010 onwards)
Bousema2016(27)	Rachuonyo, a western district in Kenya	Cluster randomised trial	5 intervention cluster. Weekly larviciding of stagnant water bodies, long lasting treated nets, Indoor residual spraying and mass drug administration to households with confirmed malaria case.(2012)	5 cluster control areas. Annual residual spraying, distribution of long lasting treated nets at antenatal clinics. No drug mass administration

62 Abbreviations: HIV Human Immunodeficiency Virus, STD Sexually Transmitted Disease,TB Tuberculosis.

For peer review only

Reference

1. Odek WO, Githuka GN, Avery L, Njoroge PK, Kasonde L, Gorgens M, et al. Estimating the size of the female sex worker population in Kenya to inform HIV prevention programming. PLoS One. 2014 Mar 3;9(3).

2. Ikpeazu A, Momah-Haruna A, Madu Mari B, Thompson LH, Ogungbemi K, Daniel U, et al. An appraisal of female sex work in Nigeria--implications for designing and scaling up HIV prevention programmes. PLoS One [Internet]. 2014;9(8):e103619. Available from: http://ovidsp.ovid.com/ovidweb.cgi?T=JS&PAGE=reference&D=med10&NEWS=N&AN=25118691

3. Mburu G, Ngin C, Tuot S, Chhoun P, Pal K, Yi S. Patterns of HIV testing, drug use, and sexual behaviors in people who use drugs: findings from a community-based outreach program in Phnom Penh, Cambodia. Addict Sci Clin Pract [Internet]. 2017 Dec 19 [cited 2020 Mar 1];12(1):27. Available from: http://ovidsp.ovid.com/ovidweb.cgi?T=JS&PAGE=reference&D=med13&NEWS=N&AN=29202872

4. Clark PA, Cegielski JP, Hassell W. TB or not TB? Increasing door-to-door response to screening. Public Health Nurs [Internet]. 1997;14(5):268–71. Available from: http://ovidsp.ovid.com/ovidweb.cgi?T=JS&PAGE=reference&D=med4&NEWS=N&AN=9342917

5. Wilkinson D, Tanser F, D. W, F. T. GIS/GPS to document increased access to community-based treatment for tuberculosis in Africa. Lancet [Internet]. 1999;354(9176):394–5. Available from: http://ovidsp.ovid.com/ovidweb.cgi?T=JS&PAGE=reference&D=emed6&NEWS=N&AN=29355762

6. Fatima R, Qadeer E, Yaqoob A, Haq MU, Majumdar SS, Shewade HD, et al. Extending "Contact Tracing" into the Community within a 50-Metre Radius of an Index Tuberculosis Patient Using Xpert MTB/RIF in Urban, Pakistan: Did It Increase Case Detection?. PLoS One [Internet]. 2016;11(11):e0165813. Available from: http://ovidsp.ovid.com/ovidweb.cgi?T=JS&PAGE=reference&D=med12&NEWS=N&AN=27898665

7. Vo LNQ, Codlin AJ, Forse RJ, Nguyen NT, Vu TN, Le GT, et al. Evaluating the yield of systematic screening for tuberculosis among three priority groups in Ho Chi Minh City, Viet Nam. Infect Dis Poverty [Internet]. 2020 Dec 1 [cited 2021 Apr 2];9(1):166. Available from: https://idpjournal.biomedcentral.com/articles/10.1186/s40249-020-00766-4

8. Mnzava AE, Sharp BL, Mthembu DJ, le Sueur D, Dlamini SS, Gumede JK, et al. Malaria control–two years' use of insecticide-treated bednets compared with insecticide house spraying in KwaZulu-Natal. South African Med J [Internet]. 2001;91(11):978–83. Available from: https://www.cochranelibrary.com/central/doi/10.1002/central/CN-00377979/full

9. Ghosh SK, Dash AP. Larvivorous fish against malaria vectors: a new outlook. Trans R Soc Trop Med Hyg [Internet]. 2007;101(11):1063–4. Available from: http://ovidsp.ovid.com/ovidweb.cgi?T=JS&PAGE=reference&D=med5&NEWS=N&AN=17804029

10. Dongus S, Nyika D, Kannady K, Mtasiwa D, Mshinda H, Fillinger U, et al. Participatory mapping of target areas to enable operational larval source management to suppress malaria vector mosquitoes in Dar es Salaam, Tanzania. Int J Health Geogr [Internet]. 2007 Sep 4 [cited 2020 Mar 2];6:37. Available from: http://www.ncbi.nlm.nih.gov/pubmed/17784963

For peer review only

11. Cook J, Aydin-Schmidt B, Gonzalez IJ, Bell D, Edlund E, Nassor MH, et al. Loop-mediated isothermal amplification (LAMP) for point-of-care detection of asymptomatic low-density malaria parasite carriers in Zanzibar. Malar J [Internet]. 2015;14:43. Available from: http://ovidsp.ovid.com/ovidweb.cgi?T=JS&PAGE=reference&D=med11&NEWS=N&AN=25627037

12. Shiff C. Utilising malariometric data in Real Time: a strategy to roll back malaria and sustain local elimination. Malar J. 2012 Oct 15;11(S1):1–1.

13. Marston L, Kelly GC, Hale E, Clements ACA, Hodge A, Jimenez-Soto E. Cost analysis of the development and implementation of a spatial decision support system for malaria elimination in Solomon Islands. Malar J [Internet]. 2014;13(325). Available from: http://www.malariajournal.com/content/pdf/1475-2875-13-325.pdf

14. Cook J, Xu W, Msellem M, Vonk M, Bergstrom B, Gosling R, et al. Mass screening and treatment on the basis of results of a Plasmodium falciparum-specific rapid diagnostic test did not reduce malaria incidence in Zanzibar. J Infect Dis [Internet]. 2015;211(9):1476–83. Available from: http://ovidsp.ovid.com/ovidweb.cgi?T=JS&PAGE=reference&D=med11&NEWS=N&AN=25429102

15. Hsiang MS, Ntshalintshali N, Kang Dufour M-S, Dlamini N, Nhlabathi N, Vilakati S, et al. Active case-finding for malaria: A three-year national evaluation of optimal approaches to detect infections and hotspots through reactive case detection in the low transmission setting of Eswatini. Clin Infect Dis [Internet]. 2019; Available from: http://ovidsp.ovid.com/ovidweb.cgi?T=JS&PAGE=reference&D=medp&NEWS=N&AN=31095677

16. Sy O, Niang EHA, Diallo A, Ndiaye A, Konate L, Ba EHCC, et al. Evaluation of the effectiveness of a targeted community-based IRS approach for malaria elimination in an area of low malaria transmission of the central-western Senegal. Parasite Epidemiol Control [Internet]. 2019;6:e00109. Available from: http://ovidsp.ovid.com/ovidweb.cgi?T=JS&PAGE=reference&D=prem&NEWS=N&AN=31193475

17. Ngo TD, Canavati SE, Dinh HS, Ngo TD, Tran DT, Martin NJ, et al. Addressing operational challenges of combatting malaria in a remote forest area of Vietnam using spatial decision support system approaches. Geospat Health [Internet]. 2019 Nov 6 [cited 2021 Apr 2];14(2):195–203. Available from: https://geospatialhealth.net/index.php/gh/article/view/770

18. Bhondoekhan FRP, Searle KM, Hamapumbu H, Lubinda M, Matoba J, Musonda M, et al. Improving the efficiency of reactive case detection for malaria elimination in southern Zambia: A cross-sectional study. Malar J [Internet]. 2020 May 7 [cited 2021 Apr 2];19(1):175. Available from: https://malariajournal.biomedcentral.com/articles/10.1186/s12936-020-03245-1

19. Goswami ND, Hecker EJ, Vickery C, Ahearn MA, Cox GM, Holland DP, et al. Geographic information system-based screening for TB, HIV, and syphilis (GIS-THIS): a cross-sectional study. Pai M, editor. PLoS One [Internet]. 2012 Oct 2 [cited 2019 Mar 29];7(10):e46029. Available from: http://ovidsp.ovid.com/ovidweb.cgi?T=JS&PAGE=reference&D=med8&NEWS=N&AN=23056227

20. Moonan PK, Oppong J, Sahbazian B, Singh KP, Sandhu R, Drewyer G, et al. What is the

For peer review only

outcome of targeted tuberculosis screening based on universal genotyping and location? Am J Respir Crit Care Med [Internet]. 2006;174(5):599–604. Available from: http://ovidsp.ovid.com/ovidweb.cgi?T=JS&PAGE=reference&D=med5&NEWS=N&AN=16728707

21. Cegielski JP, Griffith DE, McGaha PK, Wolfgang M, Robinson CB, Clark PA, et al. Eliminating tuberculosis one neighborhood at a time. Am J Public Health [Internet]. 2013 Jul [cited 2019 Feb 5];103(7):1292–300. Available from: http://www.ncbi.nlm.nih.gov/pubmed/23078465

22. De Souza Dias MCF, Dias GH, Nobre ML, M.C.F. DSD, G.H. D, M.L. N. The use of Geographical Information System (GIS) to improve active leprosy case finding campaigns in the Municipality of Mossoro, Rio Grande do Norte State, Brazil. Lepr Rev [Internet]. 2007;78(3):261–9. Available from: http://www.lepra.org.uk/lr/Sept07/Lep261-269.pdf

23. Jim R, Johnson E, Pavlin BI. Role of GIS technology during leprosy elimination efforts in Pohnpei. Pac Health Dialog [Internet]. 2010;16(1):109–14. Available from: http://ovidsp.ovid.com/ovidweb.cgi?T=JS&PAGE=reference&D=med7&NEWS=N&AN=20968242

24. Barreto JG, Bisanzio D, Frade MAC, Moraes TMP, Gobbo AR, Guimarães L de S, et al. Spatial epidemiology and serologic cohorts increase the early detection of leprosy. BMC Infect Dis [Internet]. 2015 Nov 16 [cited 2019 Mar 12];15(1):1–9. Available from: http://www.ncbi.nlm.nih.gov/pubmed/26573912

25. Srivastava A, Nagpal BN, Joshi PL, Paliwal JC, Dash AP, A. S, et al. Identification of malaria hot spots for focused intervention in tribal state of India: a GIS based approach. Int J Health Geogr [Internet]. 2009;8(101152198):30. Available from: http://ovidsp.ovid.com/ovidweb.cgi?T=JS&PAGE=reference&D=med6&NEWS=N&AN=19457227

26. Herdiana H, Fuad A, Asih PB, Zubaedah S, Arisanti RR, Syafruddin D, et al. Progress toward malaria elimination in sabang, aceh, indonesia. Am J Trop Med Hyg [Internet]. 2012;87(5 SUPPL. 1):47. Available from: http://www.ajtmh.org/content/87/5_Suppl_1/1.1.full.pdf+html

27. Bousema T, Stresman G, Baidjoe AY, Bradley J, Knight P, Stone W, et al. The Impact of Hotspot-Targeted Interventions on Malaria Transmission in Rachuonyo South District in the Western Kenyan Highlands: A Cluster-Randomized Controlled Trial. PLoS Med. 2016;13(4):e1001993–e1001993.

For peer review only

Synthesis Without Meta-analysis (SWiM) reporting items

The citation for the Synthesis Without Meta-analysis explanation and elaboration article is: Campbell M, McKenzie JE, Sowden A, Katikireddi SV, Brennan SE, Ellis S, Hartmann-Boyce J, Ryan R, Shepperd S, Thomas J, Welch V, Thomson H. Synthesis without meta-analysis (SWiM) in systematic reviews: reporting guideline BMJ 2020;368:l6890 http://dx.doi.org/10.1136/bmj.l6890

**SWiM is intended to complement and be used as an extension to PRISMA**			
**SWiM reporting item**	**Item description**	**Page in manuscript where item is reported**	**Other***
Methods			
**1** Grouping studies for synthesis	1a) Provide a description of, and rationale for, the groups used in the synthesis (e.g., groupings of populations, interventions, outcomes, study design)	6	
	1b) Detail and provide rationale for any changes made subsequent to the protocol in the groups used in the synthesis	Not applicable	
**2** Describe the standardised metric and transformation methods used	Describe the standardised metric for each outcome. Explain why the metric(s) was chosen, and describe any methods used to transform the intervention effects, as reported in the study, to the standardised metric, citing any methodological guidance consulted	7	
**3** Describe the synthesis methods	Describe and justify the methods used to synthesise the effects for each outcome when it was not possible to undertake a meta-analysis of effect estimates	7	
**4** Criteria used to prioritise results for summary and synthesis	Where applicable, provide the criteria used, with supporting justification, to select the particular studies, or a particular study, for the main synthesis or to draw conclusions from the synthesis (e.g., based on study design, risk of bias assessments, directness in relation to the review question)	6 and 7	

For peer review only - http://bmjopen.bmj.com/site/about/guidelines.xhtml

Synthesis Without Meta-analysis (SWiM) reporting items

SWiM reporting item	Item description	Page in manuscript where item is reported	Other*
**5** Investigation of heterogeneity in reported effects	State the method(s) used to examine heterogeneity in reported effects when it was not possible to undertake a meta-analysis of effect estimates and its extensions to investigate heterogeneity	7	
**6** Certainty of evidence	Describe the methods used to assess certainty of the synthesis findings	7	
**7** Data presentation methods	Describe the graphical and tabular methods used to present the effects (e.g., tables, forest plots, harvest plots).<br><br>Specify key study characteristics (e.g., study design, risk of bias) used to order the studies, in the text and any tables or graphs, clearly referencing the studies included	6,7,12	
Results			
**8** Reporting results	For each comparison and outcome, provide a description of the synthesised findings, and the certainty of the findings. Describe the result in language that is consistent with the question the synthesis addresses, and indicate which studies contribute to the synthesis	8-17	
Discussion			
**9** Limitations of the synthesis	Report the limitations of the synthesis methods used and/or the groupings used in the synthesis, and how these affect the conclusions that can be drawn in relation to the original review question	18-21	

PRISMA=Preferred Reporting Items for Systematic Reviews and Meta-Analyses.
*If the information is not provided in the systematic review, give details of where this information is available (e.g., protocol, other published papers (provide citation details), or website (provide the URL)).

For peer review only

EFFECTIVENESS OF SPATIALLY-TARGETED INTERVENTIONS FOR CONTROL OF HIV, TUBERCULOSIS, LEPROSY AND MALARIA: A SYSTEMATIC REVIEW

Journal:	*BMJ Open*
Manuscript ID	bmjopen-2020-044715.R2
Article Type:	Original research
Date Submitted by the Author:	31-May-2021
Complete List of Authors:	Khundi, McEwen; London School of Hygiene & Tropical Medicine, Medical Statistics; Malawi-Liverpool-Wellcome Trust Clinical Research Programme, Public Health<br>Carpenter, James; London School of Hygiene and Tropical Medicine, Medical Statistics; University College London, MRC Clinical Trials Unit<br>Nliwasa, Marriott; University of Malawi College of Medicine, Helse Nord Tuberculosis Initiative<br>Cohen, Ted; Yale University, School of Public Health<br>Corbett, Liz; London School of Hygiene & Tropical Medicine, Department of Clinical Research; Malawi-Liverpool-Wellcome Trust Clinical Research Programme<br>MacPherson, Peter; Liverpool School of Tropical Medicine, Department of Clinical Sciences; Malawi-Liverpool-Wellcome Trust Clinical Research Programme
**Primary Subject Heading**:	Public health
Secondary Subject Heading:	Infectious diseases, Epidemiology
Keywords:	Tuberculosis < INFECTIOUS DISEASES, Infection control < INFECTIOUS DISEASES, HIV & AIDS < INFECTIOUS DISEASES, Public health < INFECTIOUS DISEASES, Epidemiology < INFECTIOUS DISEASES, INFECTIOUS DISEASES

SCHOLARONE™ Manuscripts

I, the Submitting Author has the right to grant and does grant on behalf of all authors of the Work (as defined in the below author licence), an exclusive licence and/or a non-exclusive licence for contributions from authors who are: i) UK Crown employees; ii) where BMJ has agreed a CC-BY licence shall apply, and/or iii) in accordance with the terms applicable for US Federal Government officers or employees acting as part of their official duties; on a worldwide, perpetual, irrevocable, royalty-free basis to BMJ Publishing Group Ltd ("BMJ") its licensees and where the relevant Journal is co-owned by BMJ to the co-owners of the Journal, to publish the Work in this journal and any other BMJ products and to exploit all rights, as set out in our licence.

The Submitting Author accepts and understands that any supply made under these terms is made by BMJ to the Submitting Author unless you are acting as an employee on behalf of your employer or a postgraduate student of an affiliated institution which is paying any applicable article publishing charge ("APC") for Open Access articles. Where the Submitting Author wishes to make the Work available on an Open Access basis (and intends to pay the relevant APC), the terms of reuse of such Open Access shall be governed by a Creative Commons licence – details of these licences and which Creative Commons licence will apply to this Work are set out in our licence referred to above.

Other than as permitted in any relevant BMJ Author's Self Archiving Policies, I confirm this Work has not been accepted for publication elsewhere, is not being considered for publication elsewhere and does not duplicate material already published. I confirm all authors consent to publication of this Work and authorise the granting of this licence.

For peer review only

EFFECTIVENESS OF SPATIALLY-TARGETED INTERVENTIONS FOR CONTROL OF HIV, TUBERCULOSIS, LEPROSY AND MALARIA: A SYSTEMATIC REVIEW

Corresponding author:
McEwen Khundi
Malawi Liverpool Welcome Trust
P.0 Box 30096
Malawi,
mcewenkhundi@gmail.com
Tel: +265 1811628
Fax: +265 1875774

James Carpenter
Department of medical statistics
London School of Hygiene and Tropical Medicine
London
United Kingdom

Marriot Nliwasa
Department of Pathology
College of Medicine, Malawi
Blantyre
Malawi

Ted Cohen
School of Public Health
Yale University
Connectut, New Haven
USA

Elizabeth L Corbett
Department of Clinical Research
London School of Hygiene and Tropical Medicine
London
United Kingdom

Peter MacPherson
Department of Clinical Sciences
Liverpool School of Tropical Medicine
Liverpool
United Kingdom

Keywords: infectious diseases, community interventions, spatial hotspot, spatially targeted, systematic review.

Word Count:
Abstract: 287
Main text: 4658

For peer review only

Abstract (287 words)

Background: As infectious diseases approach global elimination targets, spatial targeting is increasingly important to identify community hotspots of transmission and effectively target interventions. We aimed to synthesise relevant evidence to define best practice approaches and identify policy and research gaps.

Objective: To systematically-appraise evidence for the effectiveness of spatially-targeted community public health interventions for HIV, Tuberculosis, Leprosy and Malaria.

Design: Systematic review.

Data sources: We searched Medline, Embase, Global Health, Web of Science and Cochrane Database of Systematic Reviews between 01 January 1993 and 22 March 2021.

Study selection: The studies had to include HIV or TB or leprosy or malaria and spatial hotspot definition, and community interventions.

Data extraction and synthesis: A data extraction tool was used. For each study, we summarised approaches to identifying hotpots, intervention design and effectiveness of the intervention.

Results: Ten studies, including one cluster randomised trials and nine with alternative designs (before-after, comparator area), satisfied our inclusion criteria. Spatially targeted interventions for HIV (one United States of America (USA) study), TB (three USA) and leprosy (two Brazil, one Federated States of Micronesia) each used household location and disease density to define hotspots followed by community-based screening. Malaria studies (one each from India, Indonesia and Kenya) used household location and disease density for hotspot identification followed by complex interventions typically combining community screening, larviciding of stagnant water bodies, indoor residual spraying and mass drug administration. Evidence of effect was mixed.

Conclusions: Studies investigating spatially targeted interventions were few in number, and mostly underpowered or otherwise limited methodologically, affecting interpretation of intervention impact. Applying advanced epidemiological methodologies supporting more robust hotspot identification and larger or more intensive interventions would strengthen the evidence-base for this increasingly important approach.

PROSPERO registration: CRD42019130133

For peer review only

Strengths and limitations of the study

- This was a thoroughly conducted systematic review which only included published literature.
- We developed the search strategy with input from infectious disease experts, statisticians and librarians, and we only included studies that met our objective assessment criteria.
- We acknowledge that some studies might still have been missed even with the systematic approach that we used.
- The effect of publication bias cannot be ruled out as it is possible that studies that had negative results might not have been published; thereby, they would not have been captured by our search strategy.
- The studies had different interventions and outcomes as such a meta-analysis was not done contrary to the initial plans.

For peer review only

Introduction

The world has made tremendous strides in controlling human immunodeficiency virus (HIV), tuberculosis (TB), leprosy and malaria epidemics. Despite impressive achievements in reductions of new cases, considerable efforts are required to meet and maintain elimination targets (1–4). Much of the current progress in disease control is attributable to the combination of facility based routine health services, supplemented by community based interventions (1–4).

The success of disease control strategies provided through facility-based services relies on prompt recognition of symptoms by the patient, early health seeking by the patient, and correct recognition and management by health providers to provide early diagnosis and effective medical care (1–4). However, health-seeking delays can be prolonged, and this strategy will also have no effect on transmission during subclinical illness (2,5–7). In low-income settings, lack of faith in the quality of services, high opportunity and indirect costs, and non-adherence to syndromic management protocols can combine to cause substantial health-seeking and diagnostic delays, undermining the effectiveness of facility-based strategies based on early diagnosis and treatment (2,4,5,8–12). Community interventions can be complementary to facility-based services but are more resource intensive, logistically challenging and are generally only justifiable when targeted at groups of people at risk or in a geographically defined area with high prevalence or incidence of disease (1–4).

In communities, HIV, TB, leprosy and malaria cluster geographically into hotspots (13–19). Hotspots are defined as areas of high incidence or prevalence compared to neighbouring geographical areas (20). For malaria, which is vector-borne, environmental characteristics conducive to replication of anopheles mosquitos are a key consideration (18). For HIV, TB and leprosy, diseases that are transmitted exclusively person-to-person, hotspots tend to be characterised by poverty, poor access to health services, overcrowding, concentrations of migrant populations, and poor housing (13–18). Since hotspots are likely to be areas of relatively high transmission, targeting interventions at hotspots may prevent many more infections and cases of disease than similar efforts in low-transmission settings or untargeted efforts (**Figure S1**) (21). Public health interventions amenable to spatially-defined hotspot targeting include: screening, case-finding, prevention (including vaccination), and improvement in access to services for diagnosis and treatment (15,20).

Community health and prevention interventions frequently target people in slums or informal urban settlements (22). In low and middle income countries (LMIC), many millions of people live in informal urban settlements (23), and disease control programmes would benefit from more precise identification of high priority areas within these to allow interventions to be delivered to communities where the impact is likely to be greatest (24,25). At the moment targeting tends to use relatively crude

For peer review only - http://bmjopen.bmj.com/site/about/guidelines.xhtml

epidemiological criteria such as age and gender (1–4,26). But since cases of the diseases in this review are known to cluster geographically, targeting of all people in a carefully identified geographically-defined hotspots offers an alternative criteria to define at-risk groups for interventions (13–19).

The main aim of this systematic review was to systematically-appraise evidence for spatially-targeted community public health interventions directed towards major infectious diseases that are transmitted in different ways: HIV is predominantly sexually transmitted; TB by respiratory droplet transmission; leprosy by direct contact and droplet transmission; and malaria by vector-borne transmission. We aim to summarise lessons learnt from evaluation of spatially targeted interventions against these diseases and to make recommendations for researchers, policymakers and disease control programmes, as well as to inform the design and evaluation of future studies investigating spatially-targeted interventions.

For peer review only

Methods

Study design

Systematic literature review.

Public involvement statement

We developed the research question for this project after realising the evidence gap on the effectiveness of spatially targeted interventions despite the growing interest that this approach has. To develop the scope of the research question we engaged experts in public health interventions of HIV, tuberculosis, leprosy and malaria. The search strategy was also developed in consultation with the experts. The results of the research will be disseminated to researchers and policy makers at local and international conferences.

Search strategy

We systematically searched the literature using major subject headings and keywords to identify published studies meeting the inclusion criteria following our published protocol (PROSPERO ID: CRD42019130133). Databases searched included: Medline, Embase, Global Health, Web of Science and the Cochrane Database of Systematic Reviews. The three central concepts included in our search strategy (**Table S1**) were the disease condition (HIV, TB, leprosy and malaria); space (techniques used to identify hotspots); and community interventions.

Eligibility criteria

We included studies published between the period from 01 January 1993 and 22 July 2019 and then updated to 22 March 2021. The start of the search period was chosen as the year that TB was declared as a "global emergency" by the World Health Organization (WHO). We focused on studies of spatially targeted interventions for HIV, TB, leprosy and malaria with the aim of identifying all the available literature on the effectiveness of spatially targeted intervention in the selected diseases, and then summarising the findings. We did not limit inclusion by age group of participants or geographic region. We included randomised controlled trials, non-randomised observational studies, before-and-after studies and time-series analysis studies. The following articles were excluded: editorials; narratives; systematic reviews; case studies; case reports; case-control studies; contact investigation studies; and non- spatially targeted community studies.

Selection of studies and data extraction

We imported studies into an Endnote (Thompsons Reuters) database, and duplicates were removed. McEwen Khundi (MK) and Marriot Nliwasa (MN) independently screened the title and abstracts

For peer review only - http://bmjopen.bmj.com/site/about/guidelines.xhtml

against inclusion and exclusion criteria (**Table S2**), identified studies eligible for full-text review, and subsequently reviewed the full text of each selected study independently. Discrepancies between MK and MN were resolved with discussion, and where agreement could not be reached, two other reviewers (Peter MacPherson (PM) and James Carpenter (JC)) participated in a consensus review.

We developed a data extraction form that was piloted on a sample of selected manuscripts. This form was independently completed for each study selected for full-text review by MK and MN.

Assessment of study quality

Two reviewers (MK and MN) assessed the quality of each study. We used separate tools to assess the methodological quality of cluster randomised trials (Cochrane Collaboration risk of bias tool) (27) and non-randomized studies (ROBINS-I tool for risk of bias assessment) (28).

Definitions

We defined spatially-targeted interventions as community interventions that targeted hotspots of disease. Hotspots were defined as sub-district geospatially-defined areas that had a high number of incident or prevalent cases of the infection or disease compared to surrounding areas, studies that based their hotspots as around diagnosed index cases were not included. In practice, as definitions varied considerably between studies, we extracted and compared hotspot definitions between studies.

Statistical analysis

The main objective of the systematic review was to compare the impact of the intervention in the intervention hotspot areas compared to control areas. We report the outcomes defined by the included studies, with outcomes being prevalence, incidence, case notification rates, and number needed to screen to identify a positive case. We calculated measures of effect and uncertainty in effect estimates using data available in manuscripts where these were not provided by the authors using R 3.61 (R Core Team). Because of high anticipated heterogeneity between and within diseases and interventions investigated, we decided not to undertake meta-analysis.

Ethics statement

This review used published data, and ethical review was not required.

Data sharing statement

The search strategy and summary data tables for this systematic review have all been included in the main text and in the appendix section.

For peer review only - http://bmjopen.bmj.com/site/about/guidelines.xhtml

Results

A total of 3919 unique abstracts were identified by the search strategy, from which 3886 were excluded from the title and abstract review, leaving 28 studies that were reviewed as full-text articles. Overall, 10 studies met the inclusion criteria (**Figure S2**). The reasons for exclusion are found in **Table S2**.

One United States of America (USA) based study was identified for HIV. Three TB studies were found: all three studies were from the USA. Three leprosy studies were found: two from Brazil and one study from the Federated States of Micronesia. For malaria, we identified three studies: one each from India, Indonesia and Kenya. The most common study design was implementation demonstration studies (i.e. studies in which spatially targeted interventions were introduced without random allocation of hotspots to study arms). Nine studies had this design: one HIV, three TB, three leprosy and two malaria. We identified one cluster randomised trial (CRT), which evaluated interventions targeted against malaria (**Table S3)**.

Table 1 summarises the characteristics of the included studies, presenting outcomes, measures of intervention effects and study quality/risk of bias assessment results. **Table 2** synthesises methods used to geolocate cases and identify hotspots in the included studies. **Table S3** has details on why studies were excluded following full text review.

Spatially-targeted HIV interventions

In Goswami et al. (29), cases of TB (N=150), HIV (N=665) and syphilis (N=155) notified between 1 Jan 2005 and 31 Dec 2007 in Wake County, North Carolina USA, were geocoded to households. Kernel density maps of the three diseases were generated; maps identified two areas with the densest number of cases (hotspots). Hotspots were defined as areas with more than 10 cases of either TB, HIV or syphilis notified per square mile during the three years. A map of streets and local businesses of Wake County was used to identify the locations of the hotspot areas.

Between 2 June 2009 and 3 Nov 2011, adult community screening for HIV was done in the defined hotspots at specific sites by community nurses and disease intervention specialists from the HIV, syphilis, and TB clinics at the county health department. Of 247 community participants screened by the study, 240 had valid HIV test results. Prevalence of HIV in hotspot areas was compared to that among patients presenting to a sexually transmitted disease (STD) clinic located outside of the hotspot areas. HIV prevalence was higher among community screened participants (8/240, 3%, 95% CI:1.4-6.5) compared to the Wake County STD clinic (64/15936, 0.4%, 95%CI: 0.3-0.5) with a risk ratio of 8.3

For peer review only - http://bmjopen.bmj.com/site/about/guidelines.xhtml

(95% CI: 4.0-17.1, p<0.001). Community HIV screening identified eight HIV positive cases, only one of whom was previously undiagnosed.

Spatially-targeted tuberculosis interventions

In Moonan et al. (24), notified TB cases from 1 Jan 1993 to 31 Dec 2000 (N=991) were geolocated to zip codes in Tarrant County, north-central Texas, USA. Three zip codes were found to have the highest TB notification rates per 100,000 of the population, in addition to having genotypically-clustered *Mycobacterium tuberculosis* isolates (24). The TB notification rates of the zip codes were 94, 55 and 32 cases per 100,000 while the case notification rate for the whole county for the same period was 5.9 cases per 100,000; 95 of 117 (81%) isolates were genotypically clustered in these three zip codes (30). Between 1 Sep 2002 and 31 Dec 2004 community-based organizations offered screening for TB and latent TB infection (LTBI) to hotspot residents. Overall, 3,645 individuals were screened. 1.2% (N = 44) people were diagnosed and treated for active TB, and 18.6% (N=681) were diagnosed and treated for LTBI. This targeted screening identified one person with active TB for every 83 screened, and one person with LTBI for every five screened. The yield of the targeted approach was considered to be more than what would be expected in a county with an active TB notification rate of 5.7 per 100,000 population year.

Goswami et al. (29) (see above in HIV section) also offered screening for LTBI to the same individuals who were offered HIV testing. Both tests were performed from the same blood sample. Hotspot prevalence was compared to LTBI prevalence at the Wake County TB clinic, which was outside of the hotspot areas. Latent TB testing at the TB clinic was offered to high risk individuals who had close contact with a recently diagnosed active TB cases, refugees and those referred by primary care providers or employers. 234/247 had valid LTBI screening results. LTBI prevalence was higher among community screened participants (36/234, 15%, 95% CI:11.0-21.7) versus 6% (95%CI: 5.6-6.6) at the TB clinic with a risk ratio of 2.5 (95% CI: 1.9-3.5, p<0.001).

In Cegielski et al. (31), notified TB cases in Smith County, Texas USA between 1985 to 1995 (N=128) and all notified LTBI cases from 1993 to 1995 (N=311) were geocoded to their households. The geocoded cases were loaded into geographical information systems (GIS) software to produce a point map of both active TB and LTBI cases. The two densest neighbourhoods of TB and LTBI cases were identified visually from the map. In 1996, study field workers went door to door in these neighbourhoods and offered tuberculin skin testing (TST). 1236 /2258 had LTBI testing and received results, 229/1236 (18.5%) were TST positive and 147 received treatment. To assess the intervention, the notified TB cases from 1996 to 2006 in Smith County were mapped to do a before and after intervention comparison. The TB notification rates in the targeted hotspots declined from 39.6 per 100,0000 people per year (95% CI:30.4-48.8) from 1985 to 1995 to zero from 1996 to 2006 (p<0.001).

For peer review only - http://bmjopen.bmj.com/site/about/guidelines.xhtml

While for the entire Smith County the TB notification rates reduced from 8.1 per 100,0000 people per year (95% CI: 5.2-11.0) from 1985 to 1995 to 3.7 per 100,000 people per year (95% CI: 1.2-6.1) from 1996 to 2006 ($p<0.001$)

Spatially-targeted leprosy interventions

In De Souza Dias et al. (32), leprosy cases notified between 1998 and 2002 (N=368) in the municipality of Mossoro, Rio Grande do Norte in Brazil were geocoded to households. The geocoded cases were used to create a density map with a radius of 100m, and four neighbourhoods with the highest concentration of cases were identified as hotspots. Four active case finding (ACF) campaigns were conducted in these hotspots between March and September 2005. Study team members went door to door to identify people with symptoms of leprosy and referred them to the nearest primary health clinic. 512 possible leprosy cases were referred, and 104 leprosy cases were diagnosed. The cases identified through hotspot active case finding represented 50% of the total cases diagnosed in the city in 2005. In addition, the case notification rate in 2005 was higher than in 2004; 9.34 per 10,000 versus 5.16 per 10,000 respectively.

In Jim et al. (33), in the state of Pohnpei in the Federated States of Micronesia, notified leprosy cases from 2002 to 2006 (N=502) were geocoded to households, producing a point-density map of 1 mile radius; areas with the densest areas were identified for ACF. During 2007 to 2008, ACF teams undertook door to door visits and screened household members for leprosy. There was an eight-fold statewide decrease in the number of households that were screened between 2007 to 2009, while the number of identified cases was similar to the pre-spatially targeted ACF campaign period of 2002 to 2006.

In Barreto et al. (25), notified leprosy cases from the two municipalities of Castanhal and Oriximinal in the state of Para, Brazil from January 2004 to February 2010 (N=633) were geocoded to households. The notified leprosy cases were aggregated by census tract to calculate census case notification rates, and a spatially empirical case detection rate was used to smooth notification rates. Census tracts were classified into four categories, with the highest category assigned to areas with >40 cases per 100,000 population. Kulldorff's spatial scan statistic was used to define groups of hotspot census tracts with statistically higher than average notification rates. Two schools were selected: one within a hotspot and one from a census tract with ≥40 notified leprosy cases per 100,000 populations. At these schools, 134 students with a mean age of 10 years were screened and 11/134, 8.2% (95% CI:3.5-13.0) were diagnosed with leprosy based on clinical signs and symptoms. The diagnostic yield in hotspot area schools was significantly higher than in students from randomly-selected schools from eight

For peer review only - http://bmjopen.bmj.com/site/about/guidelines.xhtml

municipalities in the state of Para in a cross-sectional study between 2009 to 2011: 63/1592, 3.9% (95% CI:3.0-4.9) with a risk ratio of 2.1 (95% CI: 1.1-3.8).

Malaria

In Srivastava et al. (34), notified malaria cases between 2000 to 2005 obtained from the State Department of Health in Madhya Predesh were aggregated at block and district level. Malaria hotspots were defined based on the percentage of malaria cases that had *P. falciparum* malaria of all notified cases from 2000 to 2005. Unspecified targeted malaria interventions in 2007 were evaluated for effectiveness by comparing overall number of confirmed cases notified in 2006 with those notified in 2007. Absolute numbers of notified cases decreased by 5.7% (95%CI:4.7-6.7) from 96,042 in 2006 (p < 0.05).

In Herdiana et al. (35), in Sabang island, Indonesia, documented malaria cases from 2007 to 2008 and self-reported malaria cases identified during a survey (N=319), were geocoded to households to produce point maps that classified 14 out of 18 villages as hotspots, based on absolute number of cases. From May 2010, home visits were conducted twice a month in hotspot villages, and only once in non-hotspot villages. Malaria blood smears were taken from anyone with current or recent history of fever, with smear-positive participants referred for treatment. Household contacts and neighbours within 500 meters of smear-positive participants were also screened. Interventions in hotspot areas included: improving malaria diagnostic labs, introduction of Artemisinin-based Combination Therapy (ACT) for malaria treatment, scale-up of indoor residual spraying (IRS) and distribution of long-lasting insecticide-treated nets (LLINs). The incidence of malaria in hotspot areas decreased by 30-fold from 3.18 to 0.13 per 1000 population from 2008 to 2011. (34) (35) (36)

In Bousema et al. (36), between June and July 2011 in Rachuonyo, western Kenya, 17,503 individuals were screened for a malaria prevalence survey, including *P. falciparum* antibodies. SaTScan software was used to define hotspots based on the prevalence of antibody-positivity and age-adjusted antibody density. In total 27 hotspots were identified. A randomised controlled trial randomly allocated ten hotspots 1:1 to either intervention or control. The intervention activities were weekly larviciding of stagnant water bodies, provision of LLINs, IRS and mass drug administration (MDA). MDA was only administered to households that had a confirmed malaria case; febrile individuals (temperature > 37.5^{0}C) and children aged 6 months – 15 years were offered malaria rapid diagnostic tests (RDT). The following standard interventions were available to both arms, hotspots and evaluation zones (the area 500 metres around each hotspot): annual IRS, case management at health facilities and distribution of LLINs from antenatal clinics.

For peer review only

There was no statistically significant difference in parasite prevalence in evaluation zones (area around the hotspot) at 8 weeks and 16 weeks post-intervention time points. The first evaluation zone (1-249m) at the 8th week found 3.6% (95% CI: -2.6 to 9.7%, p=0.216) and the second evaluation zone (250-500m) at the 8th week 3.8% (95% CI: -2.4 to 10.0%, p=0.187). Neither was there any significant difference in the first evaluation zone (1-249m) at the 16th week: 1.0%, (95% CI: -7.0 to 9.1%, p=0.713) and the second evaluation zone (250-500m) at 16th week: 1.0% (95% CI: -8.3 to 10.4%, p=0.809).

Influence of study quality on results

The risk of bias assessment of the studies in this review focused on how the hotspots were selected and how the intervention were assessed in each of the studies that were included. The studies in this review were found to have issues that would make them susceptible to risk of bias. Hotspot selection was based mainly on notified cases (24,25,29,32–35,37,38). Relying on notified cases alone can introduce selection bias because notified cases can over-represent cases from areas that have good access to health systems (15).

Further, identified geographical hotspots need to be investigated for stability overtime to ensure that they display spatio-temporal consistency in notification trends (15,39). This was not accounted for in the studies included in this review (24,25,40,29,32–38).

The majority of the identified studies were implementation/pragmatic studies (24,25,29,31–35,40,41). These did not randomise hotspots to interventions and therefore the studies might have compared outcomes of the study in groups of people that had baseline characteristics that were different.

For peer review only

Table 1: Characteristics of spatially targeted interventions for HIV, TB, Leprosy and Malaria

Reference, year	Outcome	Effect of intervention[1]	Risk of bias assessment
**HIV**			
Goswami 2012 (29)	Comparison of case detection yield between hotspot intervention areas and a county STD clinic over the same period (2009-2011)	HIV prevalence was higher among community screened participants (8/240, 3%, 95% CI:1.4-6.5) compared to the Wake County STD clinic (64/15936, 0.4%, 95%CI:0.3-0.5) with a risk ratio of (8.3, 95% CI: 4.0-17.1), p<0.001.	Moderate
**Tuberculosis**			
Moonan2006 (24)	Yield of TB case detection in hotspots.	Targeted screening identified one person with TB for every 83 screened and one person with LTBI for every five screened. The yield of the targeted approach was considered to be more than what would be expected in a county with an active TB notification rate of 5.7 per 100,000 population year.	Critical
Goswami 2012 (29)	Comparison of case detection yield of LTBI between hotspot areas and county TB clinic over the same period (2009-2011)	LTBI prevalence was higher among community screened participants (36/234, 15%, 95% CI:11.0-21.7) versus (541/9024, 6%, 95%CI=5.6-6.6) at the TB clinic with a risk ratio of (2.5, 95% CI: 1.9-3.5), p<0.001.	Moderate
Cegielski 2013 (31)	A before and after intervention comparison of mapped TB notification rates between 1985-1995 and 1996-2006	TB notification rates in the targeted hotspots declined from 39.6 per 100,0000 people per year (95% CI:30.4-48.8) from 1985 to 1995 to zero from 1996 to 2006 (p<0.001)	Serious
**Leprosy (three studies)**			

For peer review only - http://bmjopen.bmj.com/site/about/guidelines.xhtml

De Souza Dias 2007 (32)	Percent of notified cases attributable to the intervention, and before and after intervention case notification rate comparison.	Active case finding identified 50% of the total cases that were diagnosed in 2005. The case notification rate in 2005 was higher compared to pre intervention year 2004, 9.34 per 10000 versus 5.16 per 10000 respectively.	Serious
Jim 2010 (33)	Yield of leprosy case detection during intervention period compared to the pre-intervention period and reduction in households that needed to be screened.	Eight-fold decrease in the number of households that needed to be screened from 2007 to 2009. While still identifying a similar number of new cases to pre-spatially targeted active case finding period 2002 to 2006.	Moderate
Barreto 2015 (25)	The yield of case detection of leprosy cases in school children in hotspot intervention schools versus in children from randomly selected schools.	In the hotspot school's (11/134, 8.2%, 95% CI:3.5-13.0) students with a mean age of 10 years were diagnosed with leprosy. While (63/1592, 3.9%, 95% CI:3.0-4.9) students from randomly selected schools with a mean age of 12 years were diagnosed with leprosy with a risk ratio of (2.1, 95% CI: 1.1-3.8), $p<0.05$.	Moderate
**Malaria (four studies)**			
Srivastava 2009 (34)	Difference in absolute numbers of notified malaria cases between 2006 and 2007.	An absolute reduction in numbers of notified cases in 2007 (N = 90829) from the notified cases in 2006 (N = 96042), (5.7%, 95% CI:4.7-6.7), $p < 0.05$.	Critical
Herdiana2013 (35)	Change of malaria notification rates from pre-intervention to post-intervention period.	30-fold reduction in malaria notifications from 3.83 per 1000 in 2008 to 0.13 per 1000 in 2011.	Serious
Bousema2016 (36)	Change in parasite prevalence in the evaluation zones (1-500m from hotspots) of intervention clusters versus control clusters	The first evaluation zone 1-249m at 8th week (3.6%, 95% CI: -2.6 to 9.7%), p=0.216 and the second evaluation zone 250-500m at 8th week (3.8%, 95% CI: -2.4 to 10.0%), p=0.187. The first evaluation zone 1-249m at 16th	Low

For peer review only

		week (1.0%, 95% CI: -7.0 to 9.1%), p=0.713 and the second evaluation zone 250-500m at 16th week (1.0%, 95% CI: -8.3 to 10.4%), p=0.809	

[1] Results calculated from the data published in the papers.

Abbreviations: TB Tuberculosis, LTBI Latent Tuberculosis Infection, m Metres, N Number, STD Sexually Transmitted Disease.

For peer review only

For peer review only - http://bmjopen.bmj.com/site/about/guidelines.xhtml

349 **Table2: Geolocation of cases and hotspot identification**

Reference, year	Geolocation of cases	Hotspot identification
**HIV (one study)**		
Goswami2012 (29)	Notified cases of either Tuberculosis(TB) (N=150), HIV (N=665) or syphilis (155) between 1 Jan 2005 and 31 Dec 2007 were geolocated to households. The method for geolocation of the cases was not described in the paper.	A kernel density map of the cases was produced. Areas with the highest densities of three diseases of HIV, Syphilis and TB (greater than 10 cases per square mile) were classified as hotspots. Two hotspot neighbourhoods were identified in the county.
**Tuberculosis (four studies)**		
Moonan, 2006 (24)	Notified TB cases (N=991) from 1 Jan 1993 to 31 Dec 2000 in Tarrant County, north central Texas, USA were geolocated to zip codes using residential addresses and zip codes that patients gave at the time of diagnosis with the aid of a GIS software.	Areas with the highest TB notification rates and high percentage of genotypically clustered TB isolates were identified as hotspots. Three neighbourhood hotspots were identified.
Goswami2012 (29)	Notified cases of TB (N=150), HIV (N=665) and syphilis (N= 155) that were notified between 1 Jan 2005 and 31 Dec 2007 were geolocated to households. The method for geolocation of the households was not described in the paper	A kernel density map was developed. Areas with the highest densities of three diseases of HIV, Syphilis and TB (greater than 10 cases per square mile) were classified as hotspots. Two hotspot neighbourhoods were identified in the county.
Cegielski 2013 (31)	Notified TB cases between 1985 to 1995 (N=128) and all notified LTBI from 1993 to 1995 (N=311) were geocoded to their households using the addresses that patients gave at the time of diagnosis. In addition, field workers tracked addresses to households to get household coordinates of addresses that failed to geolocate.	The points of cases were plotted on a map and areas with the densest clusters of points of cases were identified as hotspots, 2 neighbourhoods were identified in the county.

For peer review only

**Leprosy (three studies)**		
DE Souza Dias2007 (32)	Notified leprosy cases that occurred between 1998 and 2002 (N=368) in the municipality of geocoded to households. The method for geolocation of cases was not described.	Density map with a radius of 100m of the notified leprosy cases was produced. Four hotspot areas were identified
Jim2010(33)	Notified leprosy cases from 2002 to 2006 (N=502) were geolocated to households. Field workers visited all notified cases to get household GPS coordinates using a GIS device.	A density map based on 1-mile radius of the notified leprosy cases. Areas with high concentration of cases classified as hotspots.
Barreto2015 (25)	Notified leprosy cases from January 2004 to February 2010 (n=633) were geocoded to households. Field workers visited households of registered cases to collect GPS coordinates.	Hotspots were identified using the Kulldorff's spatial scan statistic and by stratification of the leprosy notified rates. Two hotspots were identified.
**Malaria (four studies)**		
Srivastava2009 (34)	Notified malaria cases between 2000 to 2005 were obtained from the State Department of Health based on the cases notified in clinics in the blocks or districts.	Blocks or districts with a percentage of plasmodium falciparum malaria cases of all notified cases that was either 100% or consistently greater than 30% from 2000-2005 or greater than 70% in 2005
Herdiana2013 (35)	Notified malaria cases from 2007 to 2008 in addition to other self-reported malaria cases that were found during a survey (n=319) were geocoded to households. Field workers obtained the GPS coordinates of households using GIS devices.	Villages that had the majority of malaria cases were classified as hotspots. 14 out of 18 villages were identified
Bousema2016 (36)	June and July 2011, 17,503 individuals tested in a malaria prevalence survey for the prevalence of P. falciparum antibodies (AMA-1 or MSP-10). Field workers collected the GPS coordinates of the households using GPS devices.	Segments of the study area were scanned in the 2 x 4 km rolling windows and areas with higher ($p < 0.05$) prevalence of antibodies and age-adjusted antibody density than the local average values were identified as hotspots.

Abbreviations: GIS Geographical Information System, GPS Global Positioning System, HIV Human Immunodeficiency Virus, m Metres, N Number, TB Tuberculosis, STD Sexually Transmitted Disease.

For peer review only

Discussion

Main findings

The key finding of this review is that, despite increasing enthusiasm for the application of spatially targeted interventions in public health research addressing infectious diseases (42), very few studies have rigorously evaluated the effectiveness of such approaches against non-hotspot comparator areas. We identified only 10 studies conducted since 1993, nearly all of which had substantial limitations in how hotspots were defined and identified, how spatially-targeted interventions were evaluated, or how comparator areas were selected. With the limited evidence available, we found some suggestion that hotspot-targeted interventions for tuberculosis, malaria and leprosy may be efficient and effective approaches in increasing diagnostic yield, reducing unnecessary screening, and perhaps in improving disease epidemiology. However, almost all the studies were vulnerable to bias due to regression to the mean: hotspots identified by highest prevalence/incidence will typically see prevalence/incidence decline, even in the absence of an intervention. As such, spatially-targeted approaches hold promise, but require further evaluation in high-quality studies to guide policymakers considering implementing this approach.

Strengths and limitations

We found fewer studies than we anticipated, potentially due to the challenges of collecting spatial data in developing countries where most areas do not yet have municipal address systems or subdistrict postal code systems (15,43). Our search strategy was designed to be inclusive, and we did identify one previous systematic review of spatially targeted intervention for TB (44). However, for data synthesis we included only primary manuscript sources and ensured that studies included in the previous, more narrowly focused systematic review were also included here. Nevertheless, in recent years technologies for obtaining spatial data have improved, become cheaper and hence more widely available; consequently, the inclusion of spatial data within epidemiological and surveillance studies as a tool to identify disease hotspots has become more common (42). In theory, this should enable disease control programmes to collect spatial data for identifying and targeting hotspots (42)(43). Thus, in future, prioritising complete mapping of low income countries, with introduction of effective residential identification systems should be a major priority. This would allow some of the methods for geolocation of notified cases in the studies in this review, which used manual and labour intensive approaches that also had high potential for imprecision, to be replaced with more efficient approaches (33).

Investment in national geospatial and surveillance tools that allow rapid, accurate and scalable geolocation of cases from within health facilities in settings that lack postal codes would greatly

For peer review only - http://bmjopen.bmj.com/site/about/guidelines.xhtml

facilitate spatial mapping of areas of interest (45). Such tools would enable the identification of hotspots at finer scale and limit the use of arbitrary blocks (34). One important concern about targeting interventions on the basis of case-notifications alone, however, would be the potential for ascertainment bias to further disadvantage underserved populations from which cases are already under-notified due to limited access to health services (46). For populations where this is likely, the ideal approach would be to investigate disease burden using prevalence surveys, or developing models that include data on geographical distribution of measures of poverty and health service access that can be then allow routine notification data to be adjusted to account for likely under-ascertainment (46).

We report a wide variety of interventions in the studies included in this systematic review. For HIV (29), TB (24,29,31) and leprosy (25,32,33), studies tended to focus on community screening interventions in suspected geographic hotspots. Malaria studies (34–36), however, were more focused on complex interventions that combined community screening and mass drug administration, with environmental measures to address the vector, including larviciding of stagnant water bodies and indoor residual spraying.

Despite the above reservations, the studies that we have identified demonstrate the feasibility of the spatially targeted approach to direct resources towards community-based interventions. For case-finding interventions or community screening, spatially targeted interventions are a compromise between tracing known contacts of infectious cases and active case finding interventions across the entire general populations or sub-population (i.e. whole urban slums) (47). One limitation of contact tracing is that it misses out cases that are unknown to the index case, and so will fail to identify cases arising from transmission to casual contacts outside of the household (48,49). Spatially targeted interventions become increasingly important for diseases that are approaching elimination, since these conditions predispose the disease cases to cluster into disease hotspots (13–19) . Hence spatially targeted interventions can help the most successful control programmes to systematically identify and address residual disease hotspots (12,19,32,50).Among all of the studies reviewed here, only one (Bousema et al.) (36) was assessed as having low risk of bias. This was a malaria intervention that evaluated the impact of a spatially targeted intervention in pre-defined inner and outer regions of hotspots (evaluation zones). This study design allows the evaluation of how targeting the hotspots benefits the individuals that are in the hotspot as well as the individuals that are in the outer regions of the hotspot. This in turn allows assessment of the impact of the intervention on reducing transmission. For vector borne diseases, including malaria, consideration need to be given to the mobility of the vectors themselves when considering spatially targeted interventions (51) i.e. if

For peer review only

mosquitoes are able to travel over moderate distances the effect of a hotspot targeted intervention might end up being diluted.

However, this study had several limitations (36). It was underpowered to detect small changes due to the hotspot targeted intervention. In addition, the intervention was also done in one transmission season which might not have been enough to interrupt the epidemic (36). The area of study might also not have been suitable for a hotspot targeted intervention because even though malaria was heterogeneous there might have been widespread transmission of malaria going on in the non-hotspot areas; this might have meant that a non-targeted approach would have been more appropriate for this setting (52).

Overall, our systematic review's limitation was that the studies we identified were heterogeneous, and we were unable to do a meta-analysis as planned. Also, the studies' methodological quality had challenges due to the lack of validation of disease hotspots and the lack of random allocation of clusters to interventions. We also only included manuscripts in English due to resource limitations, and only four diseases were included. Despite these limitations, we were able to show the potential that spatially targeted interventions have towards improving disease epidemiology.

Recommendations

In general spatially-targeted interventions are only justifiable in settings where the epidemic is heterogenous and secondly where the non-hotspot areas cannot sustain sufficient transmission of the disease (36). The influence of the hotspots on the epidemic of the disease in the surrounding communities also depends on the effective contact rates between the residents of the hotspots and the surrounding areas; in the case of malaria this also depends on how far mosquitoes can travel between the hotspots and the surrounding communities. Careful attention needs also to be given to identifying hotspots to make sure that the hotspots capture the true burden of the disease by making sure that the hotspots are persistent in time and are adjusted for confounding (51–53).

Conclusion

The rapid increase in cheap, reliable, geolocation technology now provides such a significant tool in the fight to control and eradicate endemic and epidemic infectious diseases that support for rapid development of effective patient mapping systems should be considered essential for health systems in low income countries. Ideally, mapping should be combined with statistical modelling to identify hotspots with the most pressing need for intervention. In this systematic review we provide some evidence in support of the effectiveness of spatially targeted intervention, but with the major conclusion being that the current body of evidence is weak. There is an urgent need to define optimal

For peer review only

For peer review only - http://bmjopen.bmj.com/site/about/guidelines.xhtml

methodology, allowing recommendations to be made to support the design of more rigorous studies that allow clear evaluation of likely impact to be made for the major categories of infectious diseases, including vector-borne and those transmitted person-to-person. Recommendations should include strategies for timely identification of hotspots and the critical aspects needed to measure the effect of intervention strategies targeting those hotspots.

For peer review only

For peer review only - http://bmjopen.bmj.com/site/about/guidelines.xhtml

Declarations

Ethics approval and consent to participate

This review used published data, and ethical review was not required.

Consent for publication

Not applicable

Availability of data and materials

The search strategy and summary data tables for this systematic review have all been included in the main text and in the appendix section.

Competing interests

There are no conflicts of interest to declare.

Funding

This work was supported by Overseas Collaborative Site Staff-Studentship included as part of Professor Elizabeth Corbett's (ELC) Senior Research Fellowship in Clinical Science from Wellcome Trust ("Sustainable Community Action for Lung hEalth (SCALE): a cluster-randomised trial in Blantyre, Malawi." WT200901/Z/16/Z.). The funders had no role in study design; in the collection, analysis, and interpretation of data; in the writing of the report; and in the decision to submit the paper for publication. Peter MacPherson (PM) is funded by Wellcome Trust (206575)

Authors' contributions

MK, PM, ELC, and James Carpenter (JC) designed the study. MK, PM, JC and MN contributed to the data collection and management. MK, PM, JC and MN contributed to the data analysis. MK, PM, ELC, JC, Ted Cohen (TC) and MN contributed to the data interpretation. MK wrote the first draft. MK, PM, ELC, JC, TC and MN reviewed, contributed to, and approved the final draft. All authors read and approved the final manuscript

Acknowledgements

We would like to thank the LSHTM library staff for assisting us with the formulation of the search strategy.

For peer review only

References

1. United Nations Joint Programme on HIV/AIDS (UNAIDS). UNAIDS data 2018 [Internet]. Geneva; 2018. Available from: http://www.unaids.org/sites/default/files/media_asset/unaids-data-2018_en.pdf

2. World Health Organisation. Global tuberculosis report 2018. Geneva; 2018.

3. World Health Oganisation. Weekly leprosy epidemiological report 31AUG2018 [Internet]. Geneva; 2018 [cited 2019 Feb 1]. Available from: http://apps.who.int/iris/bitstream/hand

4. World Health Organisation. World malaria report 2018 [Internet]. Geneva; 2018 [cited 2019 Feb 1]. Available from: www.who.int/malaria

5. World Health Organisation. Global leprosy strategy 2016-2020. Geneva; 2015.

6. World Health Organisation. Frequently asked questions on leprosy [Internet]. Geneva; 2013. Available from: http://apps.searo.who.int/PDS_DOCS/B5044.pdf

7. Almeida ACG, Kuehn A, Castro AJM, Vitor-Silva S, Figueiredo EFG, Brasil LW, et al. High proportions of asymptomatic and submicroscopic Plasmodium vivax infections in a peri-urban area of low transmission in the Brazilian Amazon. Parasit Vectors [Internet]. 2018 Dec 20 [cited 2019 Feb 1];11(1):194. Available from: http://www.ncbi.nlm.nih.gov/pubmed/29558985

8. Ngwira LG, Dowdy DW, Khundi M, Barnes GL, Nkhoma A, Choko AT, et al. Delay in seeking care for tuberculosis symptoms among adults newly diagnosed with HIV in rural Malawi. INT J TUBERC LUNG DIS [Internet]. 2018 Mar 1 [cited 2019 Feb 1];22(3):280–6. Available from: http://dx.doi.org/10.5588/ijtld.17.0539

9. Fomundam HN, Tesfay AR, Mushipe SA, Mosina MB, Boshielo CT, Nyambi HT, et al. Prevalence and predictors of late presentation for HIV care in South Africa. S Afr Med J [Internet]. 2017 Nov 27 [cited 2019 Feb 1];107(12):1058–64. Available from: http://www.ncbi.nlm.nih.gov/pubmed/29262956

10. Sulat JS, Prabandari YS, Sanusi R, Hapsari ED, Santoso B. The impacts of community-based HIV testing and counselling on testing uptake. J Heal Res [Internet]. 2018;JHR-01-2018-015. Available from: http://www.emeraldinsight.com/doi/10.1108/JHR-01-2018-015

11. Ho J, Fox GJ, Marais BJ. Passive case finding for tuberculosis is not enough. Int J Mycobacteriology [Internet]. 2016 Dec 1 [cited 2019 Mar 7];5(4):374–8. Available from: https://www.sciencedirect.com/science/article/pii/S2212553116301595?via%3Dihub

12. World Health Organisation. A framework for malaria elimination global Malaria programme [Internet]. Geneva; 2017 [cited 2019 Mar 13]. Available from: https://apps.who.int/iris/bitstream/handle/10665/254761/9789241511988-

For peer review only - http://bmjopen.bmj.com/site/about/guidelines.xhtml

eng.pdf;jsessionid=FEBA2DD0A83E29D8833B238DAD060E96?sequence=1

13. Brian J. Coburn, Justin T. Okano SB, Coburn BJ, Okano JT, Blower S. Using geospatial mapping to design HIV elimination strategies for sub-Saharan Africa. Sci Transl Med [Internet]. 2017;9(383 PG-). Available from: NS -

14. Cuadros DF, Li J, Branscum AJ, Akullian A, Jia P, Mziray EN, et al. Mapping the spatial variability of HIV infection in Sub-Saharan Africa: Effective information for localized HIV prevention and control. Sci Rep. 2017;7(1):1–11.

15. Shaweno D, Karmakar M, Alene KA, Ragonnet R, Clements AC, Trauer JM, et al. Methods used in the spatial analysis of tuberculosis epidemiology: a systematic review. BMC Med [Internet]. 2018 [cited 2019 Feb 20];16(1):193. Available from: https://bmcmedicine.biomedcentral.com/articles/10.1186/s12916-018-1178-4

16. Monteiro LD, Martins-Melo FR, Brito AL, Alencar CH, Heukelbach J. Spatial patterns of leprosy in a hyperendemic state in Northern Brazil, 2001-2012. Rev Saude Publica. 2015;49.

17. Alencar CH, Ramos AN, dos Santos ES, Richter J, Heukelbach J. Clusters of leprosy transmission and of late diagnosis in a highly endemic area in Brazil: Focus on different spatial analysis approaches. Trop Med Int Heal. 2012;17(4):518–25.

18. Sluydts V, Heng S, Coosemans M, Van Roey K, Gryseels C, Canier L, et al. Spatial clustering and risk factors of malaria infections in Ratanakiri Province, Cambodia. Malar J [Internet]. 2014 Dec 30 [cited 2019 Feb 1];13(101139802):387. Available from: http://ovidsp.ovid.com/ovidweb.cgi?T=JS&PAGE=reference&D=med10&NEWS=N&AN=25269827

19. Shaweno D, Trauer JM, Denholm JT, McBryde ES. The role of geospatial hotspots in the spatial spread of tuberculosis in rural Ethiopia: a mathematical model. R Soc open Sci [Internet]. 2018;5(9):180887. Available from: http://ovidsp.ovid.com/ovidweb.cgi?T=JS&PAGE=reference&D=prem&NEWS=N&AN=30839742

20. Lessler J, Azman AS, McKay HS, Moore SM. What is a Hotspot Anyway? Am J Trop Med Hyg [Internet]. 2017 Jun [cited 2019 Feb 18];96(6):1270–3. Available from: http://www.ncbi.nlm.nih.gov/pubmed/28719289

21. Shrestha S, Chatterjee S, Rao KD, Dowdy DW. Potential impact of spatially targeted adult tuberculosis vaccine in Gujarat, India. J R Soc Interface [Internet]. 2016;13(116). Available from: http://ovidsp.ovid.com/ovidweb.cgi?T=JS&PAGE=reference&D=med12&NEWS=N&AN=27009179

For peer review only

For peer review only - http://bmjopen.bmj.com/site/about/guidelines.xhtml

22. Lonnroth, Knut. Systematic screening for active tuberculosis Principles and recommendations [Internet]. [cited 2019 May 27]. Available from: https://www.who.int/tb/publications/Final_TB_Screening_guidelines.pdf

23. United Nations Human Settlements Programme. SLUM ALMANAC 2015 2016 Tracking Improvement in the Lives of Slum Dweller [Internet]. Nairobi; 2015. Available from: https://unhabitat.org/slum-almanac-2015-2016/

24. Moonan PK, Oppong J, Sahbazian B, Singh KP, Sandhu R, Drewyer G, et al. What is the outcome of targeted tuberculosis screening based on universal genotyping and location? Am J Respir Crit Care Med [Internet]. 2006;174(5):599–604. Available from: http://ovidsp.ovid.com/ovidweb.cgi?T=JS&PAGE=reference&D=med5&NEWS=N&AN=16728707

25. Barreto JG, Bisanzio D, Frade MAC, Moraes TMP, Gobbo AR, Guimarães L de S, et al. Spatial epidemiology and serologic cohorts increase the early detection of leprosy. BMC Infect Dis [Internet]. 2015 Nov 16 [cited 2019 Mar 12];15(1):1–9. Available from: http://www.ncbi.nlm.nih.gov/pubmed/26573912

26. Wold Health Organisation. Systematic screening for active tuberculosis. Geneva; 2013.

27. Higgins JPT, Altman DG, Gøtzsche PC, Jüni P, Moher D, Oxman AD, et al. The Cochrane Collaboration's tool for assessing risk of bias in randomised trials. BMJ. 2011 Oct 29;343(7829).

28. Sterne JA, Hernán MA, Reeves BC, Savović J, Berkman ND, Viswanathan M, et al. ROBINS-I: A tool for assessing risk of bias in non-randomised studies of interventions. BMJ. 2016 Oct 12;355.

29. Goswami ND, Hecker EJ, Vickery C, Ahearn MA, Cox GM, Holland DP, et al. Geographic information system-based screening for TB, HIV, and syphilis (GIS-THIS): a cross-sectional study. Pai M, editor. PLoS One [Internet]. 2012 Oct 2 [cited 2019 Mar 29];7(10):e46029. Available from: http://ovidsp.ovid.com/ovidweb.cgi?T=JS&PAGE=reference&D=med8&NEWS=N&AN=23056227

30. Moonan PK, Bayona M, Quitugua TN, Oppong J, Dunbar D, Jost KCJ, et al. Using GIS technology to identify areas of tuberculosis transmission and incidence. Int J Health Geogr [Internet]. 2004;3(1):23. Available from: http://ovidsp.ovid.com/ovidweb.cgi?T=JS&PAGE=reference&D=medp&NEWS=N&AN=15479478

31. Cegielski JP, Griffith DE, McGaha PK, Wolfgang M, Robinson CB, Clark PA, et al. Eliminating

For peer review only

For peer review only - http://bmjopen.bmj.com/site/about/guidelines.xhtml

tuberculosis one neighborhood at a time. Am J Public Health [Internet]. 2013 Jul [cited 2019 Feb 5];103(7):1292–300. Available from: http://www.ncbi.nlm.nih.gov/pubmed/23078465

32. De Souza Dias MCF, Dias GH, Nobre ML, M.C.F. DSD, G.H. D, M.L. N. The use of Geographical Information System (GIS) to improve active leprosy case finding campaigns in the Municipality of Mossoro, Rio Grande do Norte State, Brazil. Lepr Rev [Internet]. 2007;78(3):261–9. Available from: http://www.lepra.org.uk/lr/Sept07/Lep261-269.pdf

33. Jim R, Johnson E, Pavlin BI. Role of GIS technology during leprosy elimination efforts in Pohnpei. Pac Health Dialog [Internet]. 2010;16(1):109–14. Available from: http://ovidsp.ovid.com/ovidweb.cgi?T=JS&PAGE=reference&D=med7&NEWS=N&AN=20968242

34. Srivastava A, Nagpal BN, Joshi PL, Paliwal JC, Dash AP, A. S, et al. Identification of malaria hot spots for focused intervention in tribal state of India: a GIS based approach. Int J Health Geogr [Internet]. 2009;8(101152198):30. Available from: http://ovidsp.ovid.com/ovidweb.cgi?T=JS&PAGE=reference&D=med6&NEWS=N&AN=19457227

35. Herdiana H, Fuad A, Asih PB, Zubaedah S, Arisanti RR, Syafruddin D, et al. Progress toward malaria elimination in sabang, aceh, indonesia. Am J Trop Med Hyg [Internet]. 2012;87(5 SUPPL. 1):47. Available from: http://www.ajtmh.org/content/87/5_Suppl_1/1.1.full.pdf+html

36. Bousema T, Stresman G, Baidjoe AY, Bradley J, Knight P, Stone W, et al. The Impact of Hotspot-Targeted Interventions on Malaria Transmission in Rachuonyo South District in the Western Kenyan Highlands: A Cluster-Randomized Controlled Trial. PLoS Med. 2016;13(4):e1001993–e1001993.

37. Tripepi G, Jager KJ, Dekker FW, Zoccali C. Selection bias and information bias in clinical research. Nephron - Clin Pract. 2010;115(2).

38. Morris U, Msellem MI, Mkali H, Islam A, Aydin-Schmidt B, Jovel I, et al. A cluster randomised controlled trial of two rounds of mass drug administration in Zanzibar, a malaria pre-elimination setting-high coverage and safety, but no significant impact on transmission. BMC Med [Internet]. 2018;16(1):215. Available from: http://ovidsp.ovid.com/ovidweb.cgi?T=JS&PAGE=reference&D=medl&NEWS=N&AN=30526588

39. Mosha JF, Sturrock HJW, Greenwood B, Sutherland CJ, Gadalla NB, Atwal S, et al. Hot spot or not: a comparison of spatial statistical methods to predict prospective malaria infections. Malar J [Internet]. 2014;13(101139802):53. Available from: http://ovidsp.ovid.com/ovidweb.cgi?T=JS&PAGE=reference&D=med10&NEWS=N&AN=24517

For peer review only

For peer review only - http://bmjopen.bmj.com/site/about/guidelines.xhtml

452

40. Hsiang MS, Ntshalintshali N, Kang Dufour M-S, Dlamini N, Nhlabathi N, Vilakati S, et al. Active case-finding for malaria: A three-year national evaluation of optimal approaches to detect infections and hotspots through reactive case detection in the low transmission setting of Eswatini. Clin Infect Dis [Internet]. 2019; Available from: http://ovidsp.ovid.com/ovidweb.cgi?T=JS&PAGE=reference&D=medp&NEWS=N&AN=31095677

41. Fatima R, Qadeer E, Yaqoob A, Haq MU, Majumdar SS, Shewade HD, et al. Extending "Contact Tracing" into the Community within a 50-Metre Radius of an Index Tuberculosis Patient Using Xpert MTB/RIF in Urban, Pakistan: Did It Increase Case Detection?. PLoS One [Internet]. 2016;11(11):e0165813. Available from: http://ovidsp.ovid.com/ovidweb.cgi?T=JS&PAGE=reference&D=med12&NEWS=N&AN=27898665

42. Elliott P, Wartenberg D. Spatial epidemiology: Current approaches and future challenges. Vol. 112, Environmental Health Perspectives. Public Health Services, US Dept of Health and Human Services; 2004. p. 998–1006.

43. Dowdy DW, Dye C, Cohen T. Data Needs for Evidence-Based Decisions: A TB Modeler's "Wish List" [cited 2019 Feb 25]; Available from: https://www.ncbi.nlm.nih.gov/pmc/articles/PMC4041555/pdf/nihms584157.pdf

44. Cudahy PGT, Andrews JR, Bilinski A, Dowdy DW, Mathema B, Menzies NA, et al. Spatially targeted screening to reduce tuberculosis transmission in high-incidence settings. Lancet Infect Dis [Internet]. 2018 Dec 13 [cited 2019 Feb 16];0(0). Available from: http://www.ncbi.nlm.nih.gov/pubmed/30554997

45. MacPherson P, Khundi M, Nliwasa M, Choko AT, Phiri VK, Webb EL, et al. Disparities in access to diagnosis and care in Blantyre, Malawi, identified through enhanced tuberculosis surveillance and spatial analysis. BMC Med [Internet]. 2019 Dec 29 [cited 2019 Feb 6];17(1):21. Available from: https://bmcmedicine.biomedcentral.com/articles/10.1186/s12916-019-1260-6

46. Borgdorff MW. New measurable indicator for tuberculosis case detection. Emerg Infect Dis [Internet]. 2004 Sep [cited 2019 Feb 6];10(9):1523–8. Available from: http://www.ncbi.nlm.nih.gov/pubmed/15498151

47. Department of HIV/AIDS, Stop TB Department, World Health Organization (WHO). Guidelines for intensified tuberculosis case-finding and isoniazid preventive therapy for people living with HIV in resource-constrained settings [Internet]. Geneva; 20111 [cited 2019 May 24].

For peer review only

Available from:
https://apps.who.int/iris/bitstream/handle/10665/44472/9789241500708_eng.pdf;jsessionid=21FF5A6C6174558D73F38D25777B56F0?sequence=1

48. Crampin AC, Glynn JR, Traore H, Yates MD, Mwaungulu L, Mwenebabu M, et al. Tuberculosis transmission attributable to close contacts and HIV status, Malawi. Emerg Infect Dis. 2006;12(5):729–35.

49. Chamie G, Kato-Maeda M, Emperador DM, Wandera B, Mugagga O, Crandall J, et al. Spatial overlap links seemingly unconnected genotype-matched TB cases in rural Uganda. 2018 [cited 2019 Jan 30]; Available from: https://doi.org/10.1371/journal.pone.0192666

50. Busgeeth K, Rivett U. The use of a spatial information system in the management of HIV/AIDS in South Africa. Int J Health Geogr [Internet]. 2004;3(13). Available from: http://www.ij-healthgeographics.com/content/3/1/13/abstract

51. Bousema T, Griffin JT, Sauerwein RW, Smith DL, Churcher TS, Takken W, et al. Hitting hotspots: spatial targeting of malaria for control and elimination. PLoS Med [Internet]. 2012 Jan [cited 2019 Mar 14];9(1):e1001165. Available from: http://www.ncbi.nlm.nih.gov/pubmed/22303287

52. Stresman G, Bousema T, Cook J. Malaria Hotspots: Is There Epidemiological Evidence for Fine-Scale Spatial Targeting of Interventions? Vol. 35, Trends in Parasitology. Elsevier Ltd; 2019. p. 822–34.

53. WHO. Module 2: Screening WHO operational handbook on tuberculosis Systematic screening for tuberculosis disease [Internet]. 2021 [cited 2021 Apr 6]. Available from: https://reliefweb.int/report/world/who-consolidated-guidelines-tuberculosis-module-2-screening-systematic-screening

For peer review only

For peer review only - http://bmjopen.bmj.com/site/about/guidelines.xhtml

Appendix 1

Figure S1: Research Overview

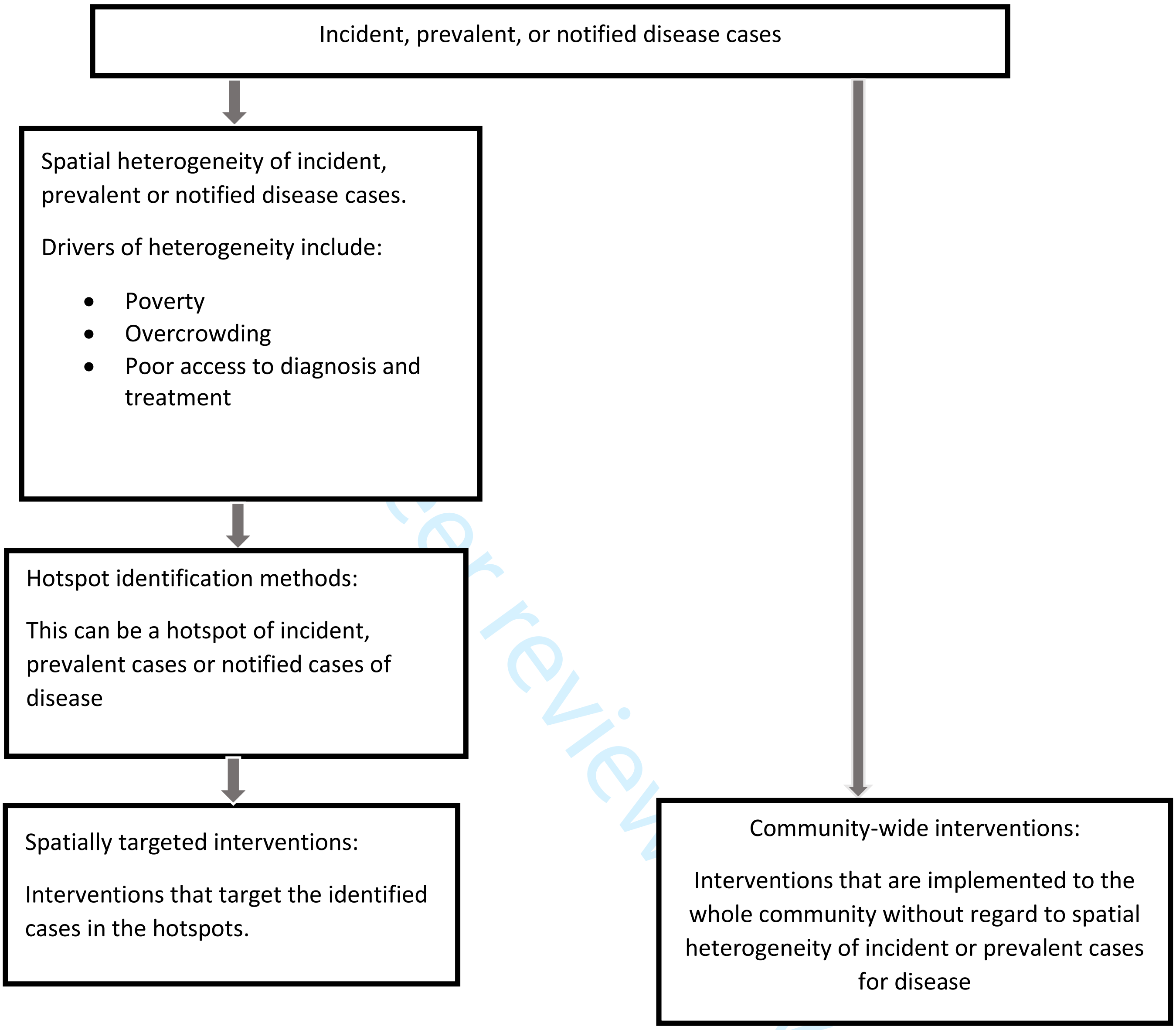

Figure S2: PRISMA flow diagram for the process of selecting included studies

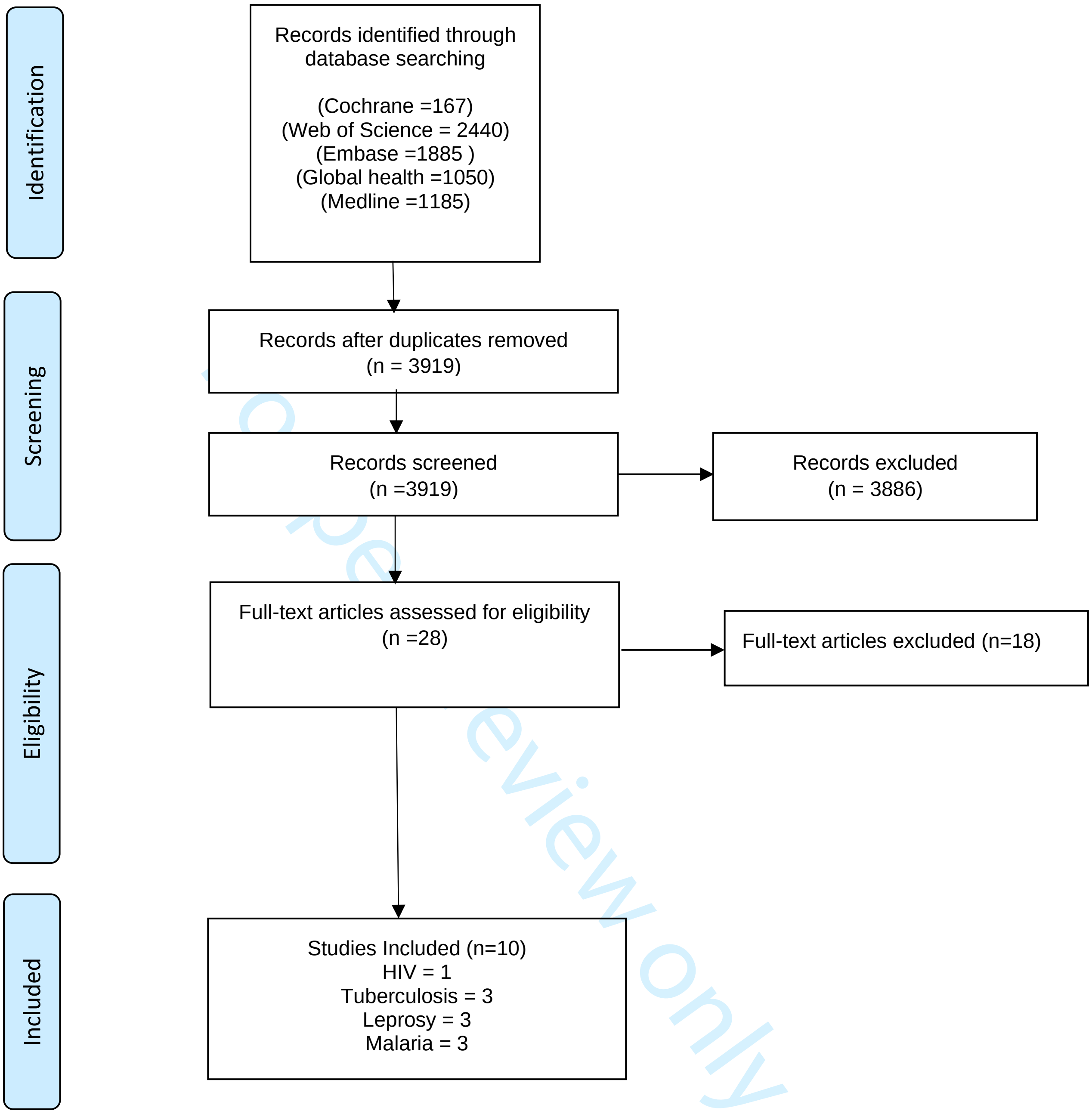

For peer review only - http://bmjopen.bmj.com/site/about/guidelines.xhtml

56 **Table S1: Search Strategy**

1	(HIV or human immunodeficiency virus or HIV-1 or HIV-2).mp.
2	exp HIV/
3	exp HIV Infections/
4	(aids or acquired immune deficiency syndrome).mp.
5	exp Acquired Immunodeficiency Syndrome/
6	(tuberculosis or TB).mp.
7	exp Tuberculosis/
8	exp Mycobacterium tuberculosis/
9	(malaria* or Plasmodium).mp.
10	exp Malaria/
11	exp Plasmodium/
12	(Leprosy or lepra or lepromatosis or hanseniase).mp.
13	exp Leprosy/
14	exp Mycobacterium leprae/
15	(spatial adj3 (analys?s or regression or temporal or autocorrelation* or auto-correlation* or statistics or epidemiology)).mp.
16	exp spatial analysis/
17	(geographic* adj3 (analys?s or regression or temporal or autocorrelation* or auto-correlation* or system* or epidemiology)).mp.
18	(GPS or global position* system* or GIS or global information system* or space?time or geospatial or hotspot* or hot-spot*).mp.
19	exp Geographic Information Systems/
20	(communit* or neighbo?rhood*).mp.
21	exp Residence Characteristics/
22	(intervention* or target*).mp.
23	((high-burden or highburden) adj3 (area* or region* or tract* or setting*)).mp.
24	(high-incidence adj3 (area* or region* or tract* or setting*)).mp.
25	(high-prevalence adj3 (area* or region* or tract* or setting*)).mp.
26	((hot-spot* or hotspot*) adj3 (area* or region* or tract*)).mp
27	1 or 2 or 3 or 4 or 5 or 6 or 7 or 8 or 9 or 10 or 11 or 12 or 13 or 14
28	15 or 16 or 17 or 18 or 19
29	20 or 21 or 22 or 23 or 24 or 25 or 26
30	27 and 28 and 29
31	limit 30 to english language
32	limit 31 to yr="1993 -Current"

57

58

For peer review only

Table S2: Reasons for exclusion of studies

Reference, year	Reason for excluding
**HIV (three studies)**	
Odek 2014 (1)	HIV hotspots not based on geolocated cases
Ikpeazu 2014 (2)	HIV hotspots not based on geolocated cases
Mburu 2017 (3)	HIV hotspots not based on geolocated cases
**Tuberculosis (four studies)**	
Clark 1999 (4)	Review
Wilkinson 1999 (5)	No hotspots based on geolocated TB cases.
Fatima 2016 (6)	Contact tracing study
Vo 2020 (7)	Hotspots were not based on TB cases
**Malaria (eleven studies)**	
Mnzava 2001 (8)	Cluster randomised trial with no standard of care arm.
Ghosh 2007 (9)	Review
Dongus 2007 (10)	Hotspot not based on geolocated malaria cases
Cook 2007 (11)	No spatially targeted intervention was evaluated
Shiff 2012 (12)	No spatially targeted intervention was evaluated
Marston 2014 (13)	Health economics study
Cook 2014 (14)	Hotspot not based on geolocated malaria cases
Hsiang 2019 (15)	Defining optimal strategies for contact tracing strategies
Ousmane 2019 (16)	No clear definition of hotspot and study evaluation not based on hotspots.
Ngo 2019 (17)	Feasibility study of acceptability of geographic information technology
Bhondoekhan 2020 (18)	Contact tracing study

For peer review only

Table S3: Full description of the characteristics of spatially targeted interventions of HIV, TB, Leprosy and Malaria

Reference, year	Setting	Study type	Intervention	Control
**HIV (one study)**				
Goswami2012(19)	Wake County, North Carolina USA	Implementation study	Community screening of HIV at sites in the hotspot areas by community nurses and disease intervention specialists from the HIV, syphilis and TB clinics at the county health department.	HIV screening of patients going to STD clinic outside of hotspot areas in the same county.
**Tuberculosis (three studies)**				
Moonan, 2006(20)	Tarrant County, north central Texas, USA	Implementation study	Community tuberculin skin testing and active TB screening by community organisations in hotspots (period 2002-2004)	No control or comparison group.
Goswami2012(19)	Wake County, North Carolina USA	Implementation study	Community screening of latent TB at sites in the hotspot areas by community nurses and disease intervention specialists from the HIV, Syphilis and TB clinics at the county health department.	Screening latent TB in patients going to a TB clinic outside of hotspot areas in the same county.
Cegielski2013 (21)	Smith County, Texas USA	Implementation study	Community screening of tuberculin skin testing and treatment of diagnosed in the hotspots (1996)	No control or comparison group
**Leprosy (three studies)**				

For peer review only

DE Souza Dias2007(22)	Municipality of Mossoro, Rio Grande do Norte, Brazil	Implementation study	Study team members moved door to door in the hotspots to identify people with symptoms of leprosy and referred them to the nearest primary health clinic for diagnosis (period 2005)	No control group
Jim2010(23)	state of Pohnpei in the Federated State of Micronesia.	Implementation study	Door to door community Screening of Leprosy cases in the hotspots (2007 to 2009)	No control group
Barreto2015(24)	Municipalities of Castanhal and Oriximinal in the state of Para, Brazil	Implementation study	Community leprosy case screening in two public schools located in the hotspot	Prevalence of leprosy in children from randomly selected schools not from the hotspot areas.
**Malaria (three studies)**				
Srivastava2009(25)	central Indian state of Madhya Predesh	Implementation study	Not specifically listed. (2007)	No control

For peer review only

Herdiana2013(26)	Island district of Sabang in Indonesia	Implementation study	Twice a month visit households. At each visit screen for malaria from household members with fever or recent history of fever. Treat all confirmed malaria cases.(May 2010 onwards)	Once a month to households in the hotspots. At each visit screen for malaria from household members with fever or recent history of fever. Treat all confirmed malaria cases. (May 2010 onwards)
Bousema2016(27)	Rachuonyo, a western district in Kenya	Cluster randomised trial	5 intervention cluster. Weekly larviciding of stagnant water bodies, long lasting treated nets, Indoor residual spraying and mass drug administration to households with confirmed malaria case.(2012)	5 cluster control areas. Annual residual spraying, distribution of long lasting treated nets at antenatal clinics. No drug mass administration

Abbreviations: HIV Human Immunodeficiency Virus, STD Sexually Transmitted Disease,TB Tuberculosis.

For peer review only

Reference

1. Odek WO, Githuka GN, Avery L, Njoroge PK, Kasonde L, Gorgens M, et al. Estimating the size of the female sex worker population in Kenya to inform HIV prevention programming. PLoS One. 2014 Mar 3;9(3).

2. Ikpeazu A, Momah-Haruna A, Madu Mari B, Thompson LH, Ogungbemi K, Daniel U, et al. An appraisal of female sex work in Nigeria--implications for designing and scaling up HIV prevention programmes. PLoS One [Internet]. 2014;9(8):e103619. Available from: http://ovidsp.ovid.com/ovidweb.cgi?T=JS&PAGE=reference&D=med10&NEWS=N&AN=25118691

3. Mburu G, Ngin C, Tuot S, Chhoun P, Pal K, Yi S. Patterns of HIV testing, drug use, and sexual behaviors in people who use drugs: findings from a community-based outreach program in Phnom Penh, Cambodia. Addict Sci Clin Pract [Internet]. 2017 Dec 19 [cited 2020 Mar 1];12(1):27. Available from: http://ovidsp.ovid.com/ovidweb.cgi?T=JS&PAGE=reference&D=med13&NEWS=N&AN=29202872

4. Clark PA, Cegielski JP, Hassell W. TB or not TB? Increasing door-to-door response to screening. Public Health Nurs [Internet]. 1997;14(5):268–71. Available from: http://ovidsp.ovid.com/ovidweb.cgi?T=JS&PAGE=reference&D=med4&NEWS=N&AN=9342917

5. Wilkinson D, Tanser F, D. W, F. T. GIS/GPS to document increased access to community-based treatment for tuberculosis in Africa. Lancet [Internet]. 1999;354(9176):394–5. Available from: http://ovidsp.ovid.com/ovidweb.cgi?T=JS&PAGE=reference&D=emed6&NEWS=N&AN=29355762

6. Fatima R, Qadeer E, Yaqoob A, Haq MU, Majumdar SS, Shewade HD, et al. Extending "Contact Tracing" into the Community within a 50-Metre Radius of an Index Tuberculosis Patient Using Xpert MTB/RIF in Urban, Pakistan: Did It Increase Case Detection?. PLoS One [Internet]. 2016;11(11):e0165813. Available from: http://ovidsp.ovid.com/ovidweb.cgi?T=JS&PAGE=reference&D=med12&NEWS=N&AN=27898665

7. Vo LNQ, Codlin AJ, Forse RJ, Nguyen NT, Vu TN, Le GT, et al. Evaluating the yield of systematic screening for tuberculosis among three priority groups in Ho Chi Minh City, Viet Nam. Infect Dis Poverty [Internet]. 2020 Dec 1 [cited 2021 Apr 2];9(1):166. Available from: https://idpjournal.biomedcentral.com/articles/10.1186/s40249-020-00766-4

8. Mnzava AE, Sharp BL, Mthembu DJ, le Sueur D, Dlamini SS, Gumede JK, et al. Malaria control–two years' use of insecticide-treated bednets compared with insecticide house spraying in KwaZulu-Natal. South African Med J [Internet]. 2001;91(11):978–83. Available from: https://www.cochranelibrary.com/central/doi/10.1002/central/CN-00377979/full

9. Ghosh SK, Dash AP. Larvivorous fish against malaria vectors: a new outlook. Trans R Soc Trop Med Hyg [Internet]. 2007;101(11):1063–4. Available from: http://ovidsp.ovid.com/ovidweb.cgi?T=JS&PAGE=reference&D=med5&NEWS=N&AN=17804029

10. Dongus S, Nyika D, Kannady K, Mtasiwa D, Mshinda H, Fillinger U, et al. Participatory mapping of target areas to enable operational larval source management to suppress malaria vector mosquitoes in Dar es Salaam, Tanzania. Int J Health Geogr [Internet]. 2007 Sep 4 [cited 2020 Mar 2];6:37. Available from: http://www.ncbi.nlm.nih.gov/pubmed/17784963

11. Cook J, Aydin-Schmidt B, Gonzalez IJ, Bell D, Edlund E, Nassor MH, et al. Loop-mediated isothermal amplification (LAMP) for point-of-care detection of asymptomatic low-density malaria parasite carriers in Zanzibar. Malar J [Internet]. 2015;14:43. Available from: http://ovidsp.ovid.com/ovidweb.cgi?T=JS&PAGE=reference&D=med11&NEWS=N&AN=25627037

12. Shiff C. Utilising malariometric data in Real Time: a strategy to roll back malaria and sustain local elimination. Malar J. 2012 Oct 15;11(S1):1–1.

13. Marston L, Kelly GC, Hale E, Clements ACA, Hodge A, Jimenez-Soto E. Cost analysis of the development and implementation of a spatial decision support system for malaria elimination in Solomon Islands. Malar J [Internet]. 2014;13(325). Available from: http://www.malariajournal.com/content/pdf/1475-2875-13-325.pdf

14. Cook J, Xu W, Msellem M, Vonk M, Bergstrom B, Gosling R, et al. Mass screening and treatment on the basis of results of a Plasmodium falciparum-specific rapid diagnostic test did not reduce malaria incidence in Zanzibar. J Infect Dis [Internet]. 2015;211(9):1476–83. Available from: http://ovidsp.ovid.com/ovidweb.cgi?T=JS&PAGE=reference&D=med11&NEWS=N&AN=25429102

15. Hsiang MS, Ntshalintshali N, Kang Dufour M-S, Dlamini N, Nhlabathi N, Vilakati S, et al. Active case-finding for malaria: A three-year national evaluation of optimal approaches to detect infections and hotspots through reactive case detection in the low transmission setting of Eswatini. Clin Infect Dis [Internet]. 2019; Available from: http://ovidsp.ovid.com/ovidweb.cgi?T=JS&PAGE=reference&D=medp&NEWS=N&AN=31095677

16. Sy O, Niang EHA, Diallo A, Ndiaye A, Konate L, Ba EHCC, et al. Evaluation of the effectiveness of a targeted community-based IRS approach for malaria elimination in an area of low malaria transmission of the central-western Senegal. Parasite Epidemiol Control [Internet]. 2019;6:e00109. Available from: http://ovidsp.ovid.com/ovidweb.cgi?T=JS&PAGE=reference&D=prem&NEWS=N&AN=31193475

17. Ngo TD, Canavati SE, Dinh HS, Ngo TD, Tran DT, Martin NJ, et al. Addressing operational challenges of combatting malaria in a remote forest area of Vietnam using spatial decision support system approaches. Geospat Health [Internet]. 2019 Nov 6 [cited 2021 Apr 2];14(2):195–203. Available from: https://geospatialhealth.net/index.php/gh/article/view/770

18. Bhondoekhan FRP, Searle KM, Hamapumbu H, Lubinda M, Matoba J, Musonda M, et al. Improving the efficiency of reactive case detection for malaria elimination in southern Zambia: A cross-sectional study. Malar J [Internet]. 2020 May 7 [cited 2021 Apr 2];19(1):175. Available from: https://malariajournal.biomedcentral.com/articles/10.1186/s12936-020-03245-1

19. Goswami ND, Hecker EJ, Vickery C, Ahearn MA, Cox GM, Holland DP, et al. Geographic information system-based screening for TB, HIV, and syphilis (GIS-THIS): a cross-sectional study. Pai M, editor. PLoS One [Internet]. 2012 Oct 2 [cited 2019 Mar 29];7(10):e46029. Available from: http://ovidsp.ovid.com/ovidweb.cgi?T=JS&PAGE=reference&D=med8&NEWS=N&AN=23056227

20. Moonan PK, Oppong J, Sahbazian B, Singh KP, Sandhu R, Drewyer G, et al. What is the

For peer review only

outcome of targeted tuberculosis screening based on universal genotyping and location? Am J Respir Crit Care Med [Internet]. 2006;174(5):599–604. Available from: http://ovidsp.ovid.com/ovidweb.cgi?T=JS&PAGE=reference&D=med5&NEWS=N&AN=16728707

21. Cegielski JP, Griffith DE, McGaha PK, Wolfgang M, Robinson CB, Clark PA, et al. Eliminating tuberculosis one neighborhood at a time. Am J Public Health [Internet]. 2013 Jul [cited 2019 Feb 5];103(7):1292–300. Available from: http://www.ncbi.nlm.nih.gov/pubmed/23078465

22. De Souza Dias MCF, Dias GH, Nobre ML, M.C.F. DSD, G.H. D, M.L. N. The use of Geographical Information System (GIS) to improve active leprosy case finding campaigns in the Municipality of Mossoro, Rio Grande do Norte State, Brazil. Lepr Rev [Internet]. 2007;78(3):261–9. Available from: http://www.lepra.org.uk/lr/Sept07/Lep261-269.pdf

23. Jim R, Johnson E, Pavlin BI. Role of GIS technology during leprosy elimination efforts in Pohnpei. Pac Health Dialog [Internet]. 2010;16(1):109–14. Available from: http://ovidsp.ovid.com/ovidweb.cgi?T=JS&PAGE=reference&D=med7&NEWS=N&AN=20968242

24. Barreto JG, Bisanzio D, Frade MAC, Moraes TMP, Gobbo AR, Guimarães L de S, et al. Spatial epidemiology and serologic cohorts increase the early detection of leprosy. BMC Infect Dis [Internet]. 2015 Nov 16 [cited 2019 Mar 12];15(1):1–9. Available from: http://www.ncbi.nlm.nih.gov/pubmed/26573912

25. Srivastava A, Nagpal BN, Joshi PL, Paliwal JC, Dash AP, A. S, et al. Identification of malaria hot spots for focused intervention in tribal state of India: a GIS based approach. Int J Health Geogr [Internet]. 2009;8(101152198):30. Available from: http://ovidsp.ovid.com/ovidweb.cgi?T=JS&PAGE=reference&D=med6&NEWS=N&AN=19457227

26. Herdiana H, Fuad A, Asih PB, Zubaedah S, Arisanti RR, Syafruddin D, et al. Progress toward malaria elimination in sabang, aceh, indonesia. Am J Trop Med Hyg [Internet]. 2012;87(5 SUPPL. 1):47. Available from: http://www.ajtmh.org/content/87/5_Suppl_1/1.1.full.pdf+html

27. Bousema T, Stresman G, Baidjoe AY, Bradley J, Knight P, Stone W, et al. The Impact of Hotspot-Targeted Interventions on Malaria Transmission in Rachuonyo South District in the Western Kenyan Highlands: A Cluster-Randomized Controlled Trial. PLoS Med. 2016;13(4):e1001993–e1001993.

For peer review only

Synthesis Without Meta-analysis (SWiM) reporting items

The citation for the Synthesis Without Meta-analysis explanation and elaboration article is: Campbell M, McKenzie JE, Sowden A, Katikireddi SV, Brennan SE, Ellis S, Hartmann-Boyce J, Ryan R, Shepperd S, Thomas J, Welch V, Thomson H. Synthesis without meta-analysis (SWiM) in systematic reviews: reporting guideline BMJ 2020;368:l6890 http://dx.doi.org/10.1136/bmj.l6890

**SWiM is intended to complement and be used as an extension to PRISMA**			
**SWiM reporting item**	**Item description**	**Page in manuscript where item is reported**	**Other***
Methods			
**1** Grouping studies for synthesis	1a) Provide a description of, and rationale for, the groups used in the synthesis (e.g., groupings of populations, interventions, outcomes, study design)	6	
	1b) Detail and provide rationale for any changes made subsequent to the protocol in the groups used in the synthesis	Not applicable	
**2** Describe the standardised metric and transformation methods used	Describe the standardised metric for each outcome. Explain why the metric(s) was chosen, and describe any methods used to transform the intervention effects, as reported in the study, to the standardised metric, citing any methodological guidance consulted	7	
**3** Describe the synthesis methods	Describe and justify the methods used to synthesise the effects for each outcome when it was not possible to undertake a meta-analysis of effect estimates	7	
**4** Criteria used to prioritise results for summary and synthesis	Where applicable, provide the criteria used, with supporting justification, to select the particular studies, or a particular study, for the main synthesis or to draw conclusions from the synthesis (e.g., based on study design, risk of bias assessments, directness in relation to the review question)	6 and 7	

For peer review only

Synthesis Without Meta-analysis (SWiM) reporting items

SWiM reporting item	Item description	Page in manuscript where item is reported	Other*
**5** Investigation of heterogeneity in reported effects	State the method(s) used to examine heterogeneity in reported effects when it was not possible to undertake a meta-analysis of effect estimates and its extensions to investigate heterogeneity	7	
**6** Certainty of evidence	Describe the methods used to assess certainty of the synthesis findings	7	
**7** Data presentation methods	Describe the graphical and tabular methods used to present the effects (e.g., tables, forest plots, harvest plots).<br><br>Specify key study characteristics (e.g., study design, risk of bias) used to order the studies, in the text and any tables or graphs, clearly referencing the studies included	6,7,12	
Results			
**8** Reporting results	For each comparison and outcome, provide a description of the synthesised findings, and the certainty of the findings. Describe the result in language that is consistent with the question the synthesis addresses, and indicate which studies contribute to the synthesis	8-17	
Discussion			
**9** Limitations of the synthesis	Report the limitations of the synthesis methods used and/or the groupings used in the synthesis, and how these affect the conclusions that can be drawn in relation to the original review question	18-21	

PRISMA=Preferred Reporting Items for Systematic Reviews and Meta-Analyses.
*If the information is not provided in the systematic review, give details of where this information is available (e.g., protocol, other published papers (provide citation details), or website (provide the URL)).

For peer review only - http://bmjopen.bmj.com/site/about/guidelines.xhtml
